# Transcriptome Analysis of Genes Associated with the Artemisinin Biosynthesis by Jasmonic Acid Treatment under the Light in *Artemisia annua*

**DOI:** 10.3389/fpls.2017.00971

**Published:** 2017-06-08

**Authors:** Xiaolong Hao, Yijun Zhong, Xueqing Fu, Zongyou Lv, Qian Shen, Tingxiang Yan, Pu Shi, Yanan Ma, Minghui Chen, Xueying Lv, Zhangkuanyu Wu, Jingya Zhao, Xiaofen Sun, Ling Li, Kexuan Tang

**Affiliations:** Key Laboratory of Urban Agriculture (South) Ministry of Agriculture, Plant Biotechnology Research Center, School of Agriculture and Biology, Shanghai Jiao Tong UniversityShanghai, China

**Keywords:** *Artemisia annua*, transcriptome sequencing, jasmonic acid, light, artemisinin biosynthesis, transcriptional regulation

## Abstract

Artemisinin is a sesquiterpene lactone endoperoxide extracted from a traditional Chinese medicinal plant *Artemisia annua*. Artemisinin-based combination therapies (ACTs) are recommended as the best treatment of malaria by the World Health Organization (WHO). Both the phytohormone jasmonic acid (JA) and light promote artemisinin biosynthesis in *A. annua*. Interestingly, we found that the increase of artemisinin biosynthesis by JA was dependent on light. However, the relationship between the two signal pathways mediated by JA and light remains unclear. Here, we collected the *A. annua* seedlings of 24 h continuous light (Light), 24 h dark treatment (Dark), 4 h MeJA treatment under the continuous light conditions (Light-MeJA-4h) and 4 h MeJA treatment under the dark conditions (Dark-MeJA-4h) and performed the transcriptome sequencing using Illumina HiSeq 4000 System. A total of 266.7 million clean data were produced and assembled into 185,653 unigenes, with an average length of 537 bp. Among them, 59,490 unigenes were annotated and classified based on the public information. Differential expression analyses were performed between Light and Dark, Light and Light-MeJA-4h, Dark and Dark-MeJA-4h, Light-MeJA-4h, and Dark-MeJA-4h, respectively. Furthermore, transcription factor (TF) analysis revealed that 1588 TFs were identified and divided into 55 TF families, with 284 TFs down-regulated in the Dark relative to Light and 96 TFs up-regulated in the Light-MeJA-4h relative to Light. 8 TFs were selected as candidates for regulating the artemisinin biosynthesis and one of them was validated to be involved in artemisinin transcriptional regulation by Dual-Luciferase (Dual-LUC) assay. The transcriptome data shown in our study offered a comprehensive transcriptional expression pattern influenced by the MeJA and light in *A. annua* seedling, which will serve as a valuable resource for further studies on transcriptional regulation mechanisms underlying artemisinin biosynthesis.

## Introduction

Malaria is a parasite infection and nearly half of the population in the world is under the risk of malaria, especially in the African region. An estimated 212 million new cases of malaria and 429,000 deaths worldwide were caused in 2015 alone according to the Malaria World Report 2016 by World Health Organization (WHO). Artemisinin is a sesquiterpene lactone endoperoxide (isolated from a traditional Chinese medicinal plant *Artemisia annua*) and artemisinin-based combination therapies (ACTs) are recommended as the best treatment of malaria by the WHO (White, 2008). In fact, the discoveries concerning artemisinin against malaria were awarded the 2015 Nobel Prize in Physiology or Medicine. Although it is viable to produce semi-synthetic artemisinin via artemisinic acid (the immediate precursor of artemisinin) in yeast (Ro et al., 2006; Paddon et al., 2013), the *A. annua* plants remain the natural and main source of artemisinin. Therefore, to improve the content of artemisinin in plants, it is necessary and urgent to study the transcriptional regulation mechanisms of artemisinin biosynthesis.

Jasmonic acid (JA) can regulate the secondary metabolism in several plant species (De Geyter et al., 2012; Zhou and Memelink, 2016), including some traditional Chinese medicinal plants, like *Scutellaria baicalensis* (Zhao et al., 2016), *Salvia miltiorrhiza* (Hao et al., 2015), and *Catharanthus roseus* (van der Fits and Memelink, 2000). It is reported that the JA treatment could simultaneously stimulate artemisinin biosynthesis and promote the formation of glandular trichomes in *A. annua* (Maes et al., 2011). However, only a few transcription factors (TFs) have been reported to be involved in the regulation of artemisinin biosynthesis in JA signaling. AaWRKY1 was the first isolated TF in *A. annua* (Ma et al., 2009). The expression of *AaWRKY1* was induced rapidly by MeJA treatment and *AaADS* was identified as the target gene of AaWRKY1 in the regulation of artemisinin biosynthesis in *A. annua* (Ma et al., 2009). Furthermore, JA-responsive AaERF1 and AaERF2 could positively regulate the transcription of *AaADS* and *AaCYP71AV1*, and increased the accumulation of artemisinin and artemisinic acid in the overexpressed transgenic *A. annua* plants (Yu et al., 2012; Lu et al., 2013a). In addition, overexpression of JA-responsive AaMYC2 in *A. annua* significantly activated the transcription of *AaCYP71AV1* and *AaDBR2*, and resulted in the increase of artemisinin content (Shen et al., 2016).

It is well-known that light is an essential environmental factor which affects the production of some secondary metabolites (Ramakrishna and Ravishankar, 2011). The regulation mechanisms of light on secondary metabolite biosynthesis like anthocyanin were studied in recent years. In eggplant (*Solanum melongena* L.), light regulated anthocyanin accumulation by way that SmCRY interaction with SmCOP1 prevented E3 ligase activity of SmCOP1 for ubiquitinating and degrading SmHY5 and SmMYB1 (Jiang et al., 2016). Consequently, SmHY5 and SmMYB1 accumulated and combined with the promoters of downstream anthocyanin synthesis genes (*SmCHS* and *SmDFR*) in light (Jiang et al., 2016). Similarly, nuclear depletion of the MdCOP1s protein in apple (*Malus domestica*) prevented the MdMYB1 protein from being ubiquitinated and degraded in light (Li et al., 2012). As a result, MdMYB1 bound to the promoters of anthocyanin biosynthetic genes, such as *MdUFGT* and *MdDFR*, to activate their expression and facilitate light-induced anthocyanin biosynthesis and fruit coloration (Takos et al., 2006; Li et al., 2012). In *Arabidopsis*, the production of anthocyanin was promoted by JA in light rather than dark (Li et al., 2014). Li et al. reported that MeJA promoted the expression of anthocyanin biosynthesis genes, such as *DFR, UF3GT*, and *LDOX* under far-red light (Li et al., 2014). And this promotion was dependent on phyA signaling pathway including phyA, COP1, and MYB75 (Li et al., 2014).

In our study, we found that light is essential for the expression of artemisinin biosynthetic genes in *A. annua* plant, and JA promotes artemisinin biosynthesis in light but not in dark. In order to explore whether the effect of JA on the artemisinin biosynthesis is dependent on light, we comprehensively analyzed the differential gene expression profiles of 24 h continuous light (Light), 24 h dark treatment (Dark), 4 h MeJA (jasmonic acid analog) treatment under the continuous light conditions (Light-MeJA-4h) and 4 h MeJA treatment under the dark conditions (Dark-MeJA-4h) using the Illumina transcriptome sequencing. As a result, we identified some TFs in light signaling pathway that can respond to MeJA, which would aid understanding of the potential transcriptional regulation mechanisms of the increase of the artemisinin biosynthesis by JA under the light in *A. annua*.

## Materials and methods

### Plant materials

*Artemisia annua* used in this study was “Huhao 1,” which obtained from Chongqing, China and developed in Shanghai after years of selection (Shen et al., 2016). *A. annua* seeds were sown in pots filled with compost and placed in a growth chamber under the growth condition described previously (Zhang et al., 2015). *Nicotiana benthamiana* used for Dual-Luciferase (Dual-LUC) assay was also grown in pots under the same growth conditions as for *A. annua*.

### MeJA treatment under light and dark conditions

**Figure 1:**
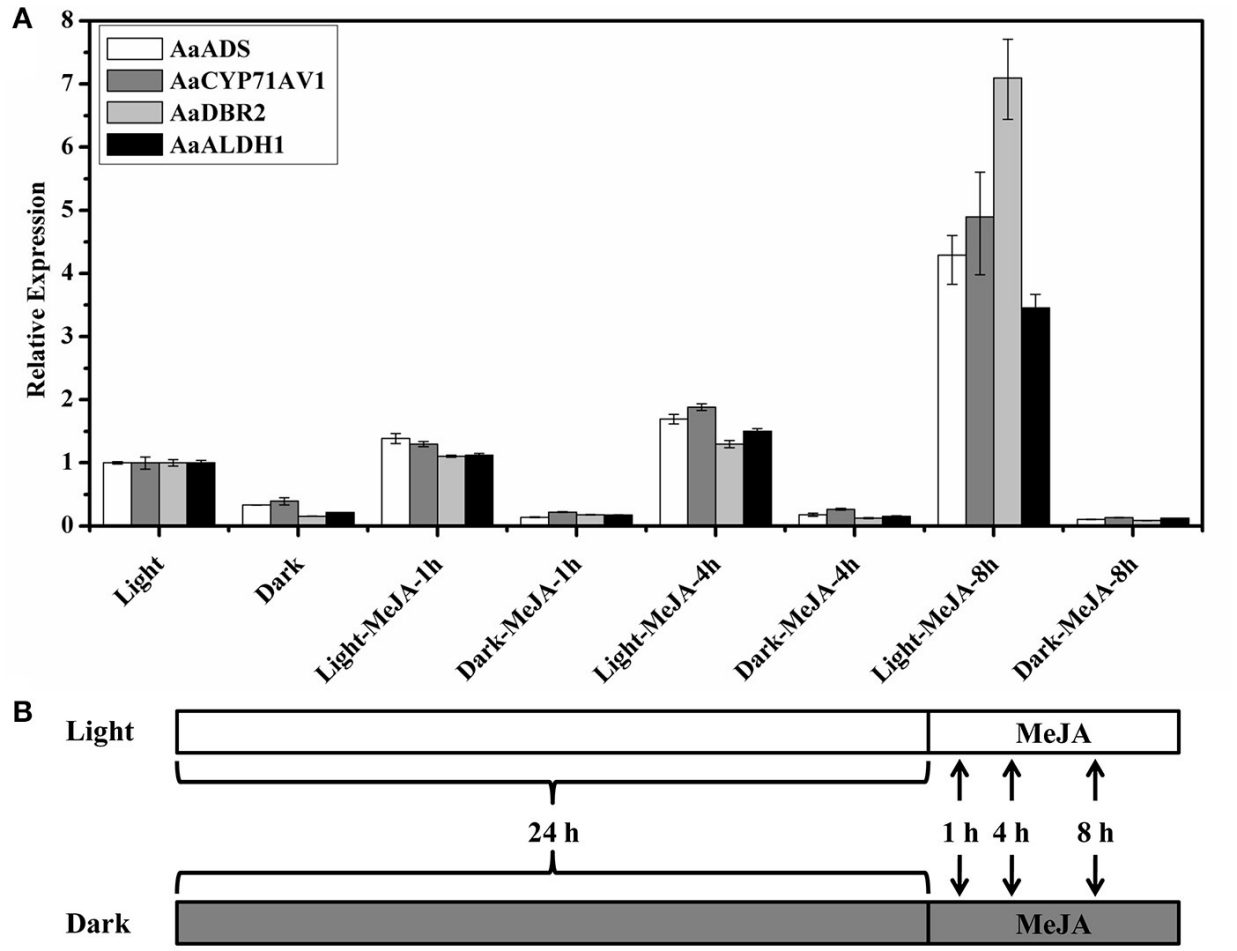
Relative expression of artemisinin biosynthetic genes in response to methyl jasmonate treatment after 8 h in the light and dark. **(A)** AaADS, amorpha-4,11-dienesynthase; AaCYP71AV1, cytochrome P450 monooxygenase; AaDBR2, artemisinic aldehyde Δ11(13) reductase; AaALDH1, aldehyde dehydrogenase 1. β*-actin* was used as the internal standard for each gene. Error bars indicate ±SD of three technical replicates. **(B)** The schematic of the experimental designs. *A. annua* seedlings were sprayed with 100 μM MeJA after pretreatment under light and dark conditions respectively for 24 h. The samples were collected after 24 h light and 24 h dark conditions and MeJA treatment for 1, 4, and 8 h under light and dark conditions.

When the *A. annua* seedlings grew in continuous white light for 2 weeks, we transferred half of them to the completely dark conditions without any light. After the independent pretreatment for 24 h, we sprayed with 100 μM MeJA (Sigma-Aldrich) under light and dark conditions (Figure 1B). So the seedlings kept under the continuous light for 24 h (Light) could be used as the control of the dark treatment for 24 h (Dark), the MeJA treatment under the light conditions (Light-MeJA) and the MeJA treatment under the dark conditions (Dark-MeJA). The leaves were collected at the continuous light with none MeJA treatment, 24 h dark treatment with none MeJA treatment, MeJA treatment for 1, 4, and 8 h under light and dark conditions (Figure 1B). All 8 samples were separately collected from three randomly selected seedlings, mixed with equivalent fresh weight (0.1 g), immediately frozen in liquid nitrogen and stored at −80°C for following RNA extraction. Three biological replicates were used for RNA extraction and quantitative real-time polymerase chain reaction (qRT-PCR) analysis.

### RNA extraction and transcriptome sequencing

The total RNA was isolated from each sample with the RNAprep Pure Plant Kit following the manufacturer's instructions (Tiangen, Beijing, China). After we analyzed the expression levels of four artemisinin biosynthetic genes (*AaADS, AaCYP71AV1, AaDBR2*, and *AaALDH1*) using qRT-PCR, we selected Light, Dark, 4 h MeJA treatment under light (Light-MeJA-4h) and 4 h MeJA treatment under the dark conditions (Dark-MeJA-4h) for the transcriptome sequencing. The cDNA libraries were constructed from total RNA of these four samples using the Illumina TruSeq™ RNA sample prep Kit (Illumina, San Diego, CA, USA). Illumina deep transcriptome sequencing was generated using Illumina HiSeq 4,000 System (2 × 151 bp read length) at Shanghai Majorbio Bio-pharm Biotechnology Co., Ltd. (Shanghai, China) (Erlich et al., 2008; Cock et al., 2010).

### Data filtering and *de novo* transcriptome assembly

We obtained the raw data of these four samples after transcriptome sequencing and we filtered the raw data before *de novo* assembly. Firstly, the raw data were conducted quality control analyses including the base percentage composition along reads, the distribution of base qualities and the distribution of base mean error ratio. Clean reads were obtained by removing the adapter sequences, low quality sequences (*Q* < 20), sequences containing more than 10% ambiguous N bases, and sequences of <20 bases in length using the SeqPrep (https://github.com/jstjohn/SeqPrep) and Sickle (https://github.com/najoshi/sickle). The remaining high-quality clean reads of these four samples were *de novo* assembled into candidate transcripts and unigenes using the Trinity *de novo* transcriptome assembly software (http://trinityrnaseq.sourceforge.net/) in absence of public reference genome for *A. annua* (Grabherr et al., 2011).

### Functional annotation and classification of unigenes

All *de novo* assembled unigenes were annotated against five public protein databases, including the NCBI non-redundant protein database (NR, http://www.ncbi.nlm.nih.gov), the Pfam (http://pfam.sanger.ac.uk/), the Swiss-Prot (http://www.expasy.ch/sprot), the Search Tool for the Retrieval of Interacting Genes (String, http://string-db.org/) and the Kyoto Encyclopedia of Genes and Genomes (KEGG, http://www.genome.jp/kegg) using BLASTX alignment with a *E*-value cut-off of 10^−5^ (Camacho et al., 2009; Grabherr et al., 2011). Blast2go (http://www.blast2go.com/b2ghome) was carried out to perform Gene Ontology (GO) classification according to molecular function (MF), biological process (BP), and cellular component (CC) base on the GO database (http://www.geneontology.org). Clusters of Orthologous Groups (COG) classification was performed using the COG database (http://www.ncbi.nlm.nih.gov/COG/) (Conesa et al., 2005). The TF families were identified and annotated using the Plant Transcription Factor Database (PlantTFDB, http://planttfdb.cbi.pku.edu.cn/).

### Determination of unigene expression level

After assembling the transcriptome, the clean reads from four transcriptome sequencing libraries were mapped back to the assembled unigenes using Bowtie (http://bowtie-bio.sourceforge.net/index.shtml) (Langmead et al., 2009). The expression levels of each unigene were normalized and calculated as the value of Fragments Per Kilobase of exon model per Million mapped reads (FPKM) using RSEM (http://deweylab.biostat.wisc.edu/rsem/), which eliminates the influence of different gene lengths and sequencing discrepancies (Li and Dewey, 2011).

### Differential expression analysis of unigenes

The differential gene expression analyses of two assigned libraries were performed using the edgeR (http://www.bioconductor.org/packages/2.12/bioc/html/edgeR.html) package (Robinson et al., 2010). The differentially expressed genes (DEGs) were screened with the threshold false discovery rate (FDR) ≤ 0.05 and the absolute value of log_2_ Fold Change ≥ 1. Subsequently, GO classifications analysis and KEGG pathway functional enrichment analysis of the DEGs were performed using GO database and KOBAS program (http://kobas.cbi.pku.edu.cn/index.php), respectively; Young et al., 2010; Xie et al., 2011).

### Gene transcript level analysis by qRT-PCR

The expression patterns of all genes were analyzed via qRT-PCR. The cDNA of all samples were prepared using PrimeScript™ RT Master Mix (Takara, Shiga, Japan). The expression levels of four artemisinin biosynthetic genes were analyzed in the all different samples before transcriptome sequencing. Eight TFs (c119965_g1, c117361_g1, c122024_g1, c95172_g1, c64067_g1, c117542_g1, c109401_g1, c113821_g1) were selected for verification of the sequencing and computational results from four sequenced samples after transcriptome sequencing. All reactions were carried out in 96-well plates on the Roche LightCycler 96 Real-Time PCR System (Roche, Basel, Switzerland) and used the SYBR Green qPCR Master Mix (Tiangen, Beijing, China) according to the manufacturer's instructions with three replicates. The cycling conditions used for qRT-PCR were: 95°C for 2 min, followed by 40 cycles of denaturation at 95°C for 20 s, annealing at 55°C for 20 s and extension at 72°C for 20 s. The relative expression levels of the genes were normalized to the internal control gene *A. annua* β*-Actin*, and determined by the relative 2^−ΔΔCt^ method. All primers used in qRT-PCR are listed in Table S1.

### Dual-LUC assay

The full-length cDNAs of the c119965_g1 was obtained according to the transcriptome sequencing results and then constructed into the pHB-YFP (yellow fluorescent protein) vector. The plasmid pHB-c119965_g1-YFP was transformed into *Agrobacterium tumefaciens* strain GV3101 to act as effector. Four reporter constructs (*pAaADS*-LUC, *pAaCYP71AV1*-LUC, *pAaDBR2*-LUC, and *pAaALDH1*-LUC) were produced by inserting the promoter of *AaADS, AaCYP71AV1, AaDBR2*, and *AaALDH1* into the pGreenII 0800-LUC vector, respectively and subsequently co-transformed with the helper plasmid pSoup19 into GV3101 to act as the reporter. pHB-YFP construct was used as negative control. All these strains were constructed and stored in our laboratory. Infiltration and detection were performed as previously described (Luo et al., 2014) with minor modifications. All overnight *A. tumefaciens* cultures were collected by centrifugation, then resuspended in MS medium to OD600 = 0.6, and incubated at room temperature for 3 h. The reporter strain harboring *pAaADS*-LUC, *pAaCYP71AV1*-LUC, *pAaDBR2*-LUC or *pAaALDH1*-LUC was mixed with the effectors strain harboring pHB-c119965_g1-YFP or pHB-YFP at the ratio of 1:1. The mixture of *A. tumefaciens* suspension was infiltrated into tobacco leaves and the negative control was infiltrated into the opposite position on the same leaves. The leaves were collected after 48 h cultivation in dark conditions and Dual-LUC assays were performed using Dual-Luciferase Reporter Assay System according to the instructions (Promega, Madison, WI, USA). Three biological repeats were measured for each sample.

## Results

### The increase of artemisinin biosynthesis by JA is dependent on light

Artemisinin is a sesquiterpene lactone endoperoxide and specially biosynthesized at the *A. annua* glandular trichomes. Four glandular trichomes specifically expressed genes (*AaADS, AaCYP71AV1, AaDBR2*, and *AaALDH1*) are involved in the biosynthesis from farnesyl diphosphate (FPP) to artemisinin (Tang et al., 2014). Interestingly, the expression of artemisinin biosynthetic genes (*AaADS, AaCYP71AV1, AaDBR2*, and *AaALDH1*) was significantly decreased after treatment under dark conditions for 24 h, with an expression about 0.1–0.4 folds of the level in the light (Figure 1). Expression of TFs and artemisinin biosynthetic genes increased rapidly after MeJA treatment in *A. annua* (Ma et al., 2009; Maes et al., 2011; Yu et al., 2012). So we focused on the early changes, from 0 to 8 h after MeJA induction. The expression of four artemisinin biosynthetic genes significantly increased after MeJA treatment under the light, which was consistent with previous reports. After the MeJA treatment under the light, the expression level of the *AaDBR2* gradually increased and reached 7.1 folds of the control at 8 h. Similarly, the expression of *AaADS, AaCYP71AV1* and *AaALDH1* also increased and reached 3.5–4.9 folds of the control at 8 h after the MeJA treatment under the light (Figure 1). However, there is no further improvement, even after treatment with MeJA in the darkness (Figure 1). Thus, the expression of artemisinin biosynthetic genes also influenced by light and the increase of artemisinin in response to MeJA treatment might dependent on light. Therefore, there might be a crosstalk between MeJA and light signaling on the regulation on artemisinin biosynthesis.

### Sequence analysis and *de novo* transcriptome assembly

**Figure 2:**
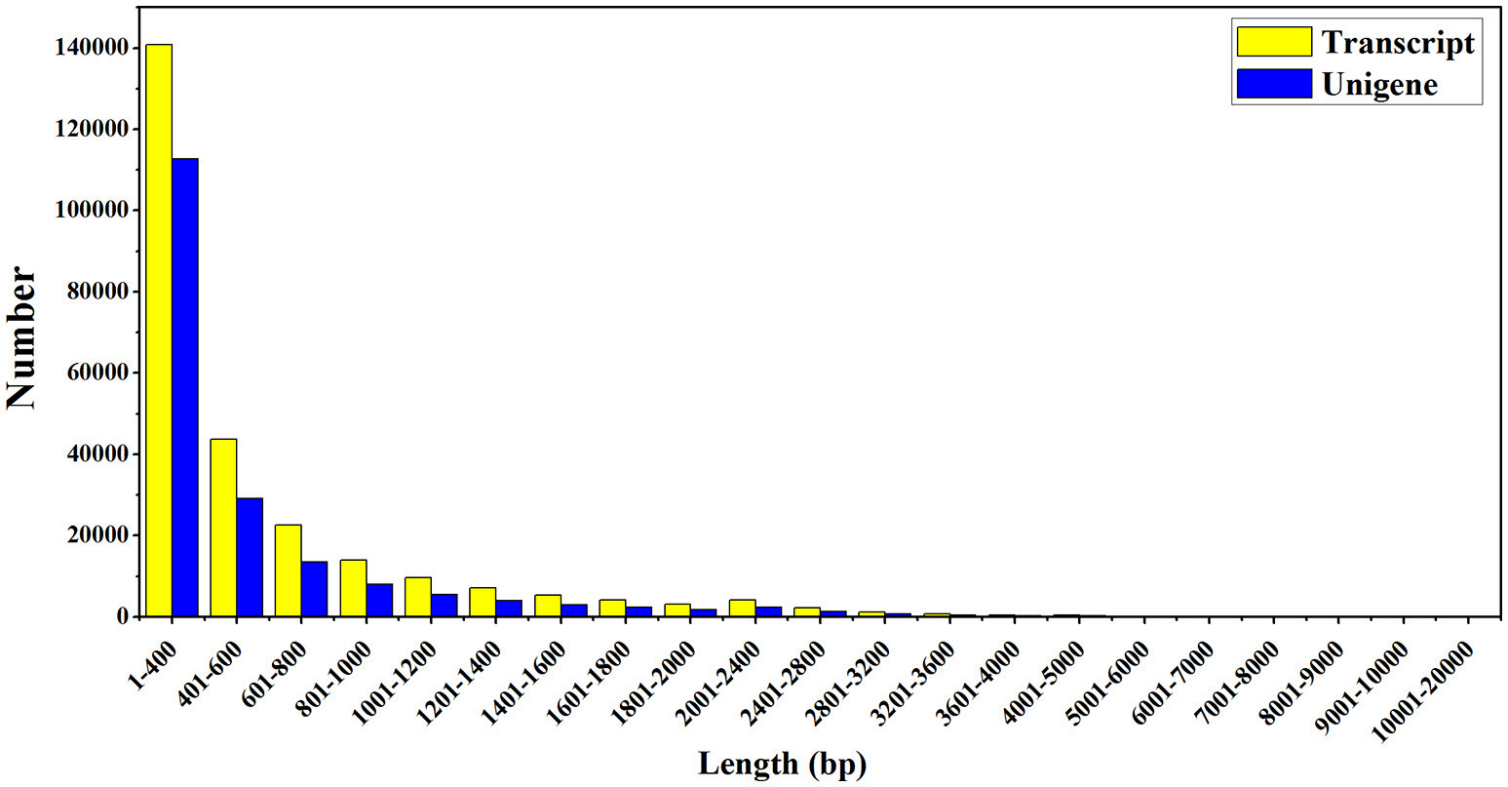
The sequence length distribution of assembled transcripts and unigenes. The horizontal and vertical axes respectively represent the different sequence length of base pairs and the number of assembled transcripts and unigenes relative to the sequence length.

To further understand the transcriptional regulatory mechanism of MeJA and light on artemisinin biosynthesis in *A. annua*, four samples were selected from seedlings under light conditions for 24 h (Light), dark conditions for 24 h (Dark), MeJA treatment for 4 h after 24 h light (Light-MeJA-4h) or 24 h dark (Dark-MeJA-4h) for transcriptome sequencing. cDNA libraries were constructed from these four samples and then sequenced using Illumina paired-end (2 × 151 bp read length) sequencing technology. In total, approximately 276.7 million Illumina raw data were generated from the four different samples (Table 1). All raw data generated in this study have been deposited in the National Center for Biotechnology Information (NCBI) and can be accessed in the Short Read Archive (SRA) Sequence Data base under accession number SRP092562. After filtering the raw data by removing the adapter sequences, low quality sequences, ambiguous sequences, and sequences of <20 bases, approximately 72.4, 67.4, 65.7, and 61.2 million clean data were left for the Light, Dark, Light-MeJA-4h and Dark-MeJA-4h transcriptomes, respectively. The summary of the raw data and clean data was shown in Table 1. All clean reads were subsequently subjected to *de novo* assembly with the Trinity program and produced 260,093 transcripts and 185,653 unigenes. The average length of transcripts and unigenes were 598 and 537 bp, respectively (Table 2). The longest sequence of transcripts and unigenes was 15,147 bp and the shortest sequence was 201 bp (Table 2). The GC content of the transcripts and unigenes distributed within 38–39% (Table 2). The length distribution of the transcripts and unigenes was indicated in Figure 2, with 11.87 and 15.02% of all transcripts and unigenes showing lengths longer than 1 kb, respectively.

**Table 1:** Summary of Illumina transcriptome sequencing analysis.

**Item**	**Sample**	**Number (n)**	**Total nucleotides (bp)**	**Error (%)**	**Q20 (%)**	**Q30 (%)**	**GC (%)**
Raw Data	Light	75,051,456	11,257,718,400	0.0113	97.12	93.75	45.06
	Dark	69,922,406	10,488,360,900	0.0113	97.1	93.77	43.51
	Light-MeJA-4h	68,206,360	10,230,954,000	0.0114	97.02	93.59	44.31
	Dark-MeJA-4h	63,489,712	9,523,456,800	0.0115	96.96	93.5	43.54
Clean Data	Light	72,388,584	10,581,333,579	0.01	98.43	95.64	44.92
	Dark	67,420,128	9,849,913,811	0.0099	98.44	95.7	43.36
	Light-MeJA-4h	65,700,898	9,608,585,030	0.01	98.4	95.57	44.17
	Dark-MeJA-4h	61,178,286	8,934,336,171	0.0101	98.38	95.53	43.38

**Table 2:** Summary of the sequence assembly results.

**Type**	**Transcripts**	**Unigenes**
Total number (n)	260,093	185,653
Total nucleotides (bp)	155,608,821	99,607,118
GC (%)	38.34	38.23
Largest length (bp)	15,147	15,147
Smallest length (bp)	201	201
Average length (bp)	598	537
N50 (bp)	837	703
N90 (bp)	258	242

### Functional annotation of unigenes

**Figure 3:**
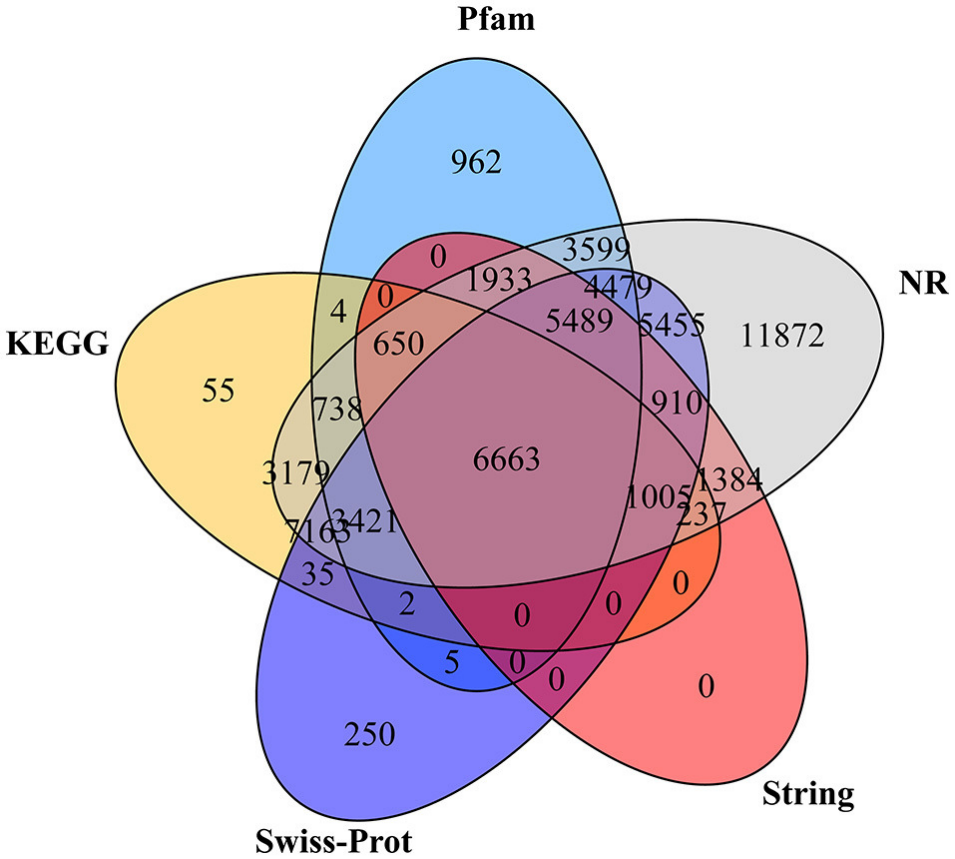
The Venn diagram shows the number of assembled unigenes annotated in five different public protein databases. The numbers in the circles indicate the number of unigenes annotated by single or multiple databases.

A total of 59,490 unigenes (32.04%) were annotated based on the information available from five public protein databases including the NR, Pfam, Swiss-Prot, String, and the KEGG using BLAST with a *E*-value cut-off of 1.0 e^−5^ (Table 3). A total of 58,177 unigenes (31.34% of the total assembled unigenes) had a match in the NR database, and 27,945 (15.05%), 34,877 (18.79%), 18,271 (9.84%), and 23,152 (12.47%) unigenes showed significant similarity to sequences in the Pfam, Swiss-Prot, String, and KEGG databases, respectively (Table 3). Among them, 6,663 unigenes showed significant matches to all five databases (Figure 3). Unigenes that were annotated as unique in public databases are as follows: 11,872 unigenes in the NR database, 962 unigenes in the Pfam database, 250 unigenes in the Swiss-Prot database, no unigenes in the String database, and 55 unigenes in the KEGG database (Figure 3). Furthermore, about 126,163 unigenes (67.96%) unaligned to any known genes.

**Table 3:** Summary of functional annotation for assembled unigenes.

**Database**	**Total unigenes**	**Annotated uingene**	**Percentage (%)**
NR	185,653	58,177	31.34
Pfam	185,653	27,945	15.05
Swiss-Prot	185,653	34,877	18.79
String	185,653	18,271	9.84
KEGG	185,653	23,152	12.47
All annotated unigenes	185,653	59,490	32.04

For the NR annotation, a total of 58,177 unigenes had significant match to known proteins in the NR database. Our results revealed that 9,482 unigenes showed closest matches with sequences from *Malus domestica*, followed by *Vitis vinifera* (4,548), *Coffea canephora* (3,613), *Solanum tuberosum* (2,497). The *e*-value distribution in the NR database revealed that 61.44% of the mapped unigenes showed significant homology (*e* < 10^−30^). Furthermore, 55.66% unigenes had high similarity (ranging from 80 to 100% similarity), whilst 36.77% of matches were of similarity ranging from 60 to 80% (Figure S1).

### Functional classification of unigenes

**Figure 4:**
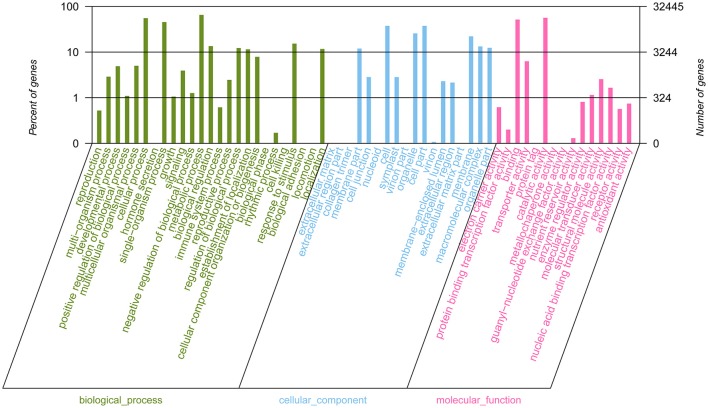
Gene Ontology (GO) functional classification of assembled unigenes. A total of 32,445 unigenes were assigned to at least one GO term and were grouped into three main GO categories and 58 groups, 25 groups in “biological process” domain, 18 in “cellular component” domain, and 15 in “molecular function” domain, respectively. The right-hand and the left-hand y-axis respectively represent the number of genes in a sub-category and the percentage of a specific sub-category of genes in each main category.

GO classification was used to classify unigene functions based on the NR annotation. Of the 185,653 assembled unigenes, 32,445 (17.48%) unigenes were successfully assigned to one or more GO terms and these unigenes were classified into three main GO categories and 58 groups (Figure 4). Within the “biological process” (BP) domain, the most evident matches were the terms “metabolic process” (21,131), “cellular process” (17,942), and “single-organism process” (14,700). In the “cellular component” (CC) domain, the assignments were mostly enriched in the terms “cell” (12,150), “cell part” (12,150), “organelle” (8,325), and “membrane” (7,172). For the “molecular function” (MF) domain, the terms “catalytic activity” (18,258), and “binding” (16,729) were mostly assigned. However, within these three categories, few unigenes (<5) were assigned to subcategories of “hormone secretion” (1, BP), “collagen trimer” (2, CC), “biological phase” (3, BP) and “protein tag” (4, MF).

**Figure 5:**
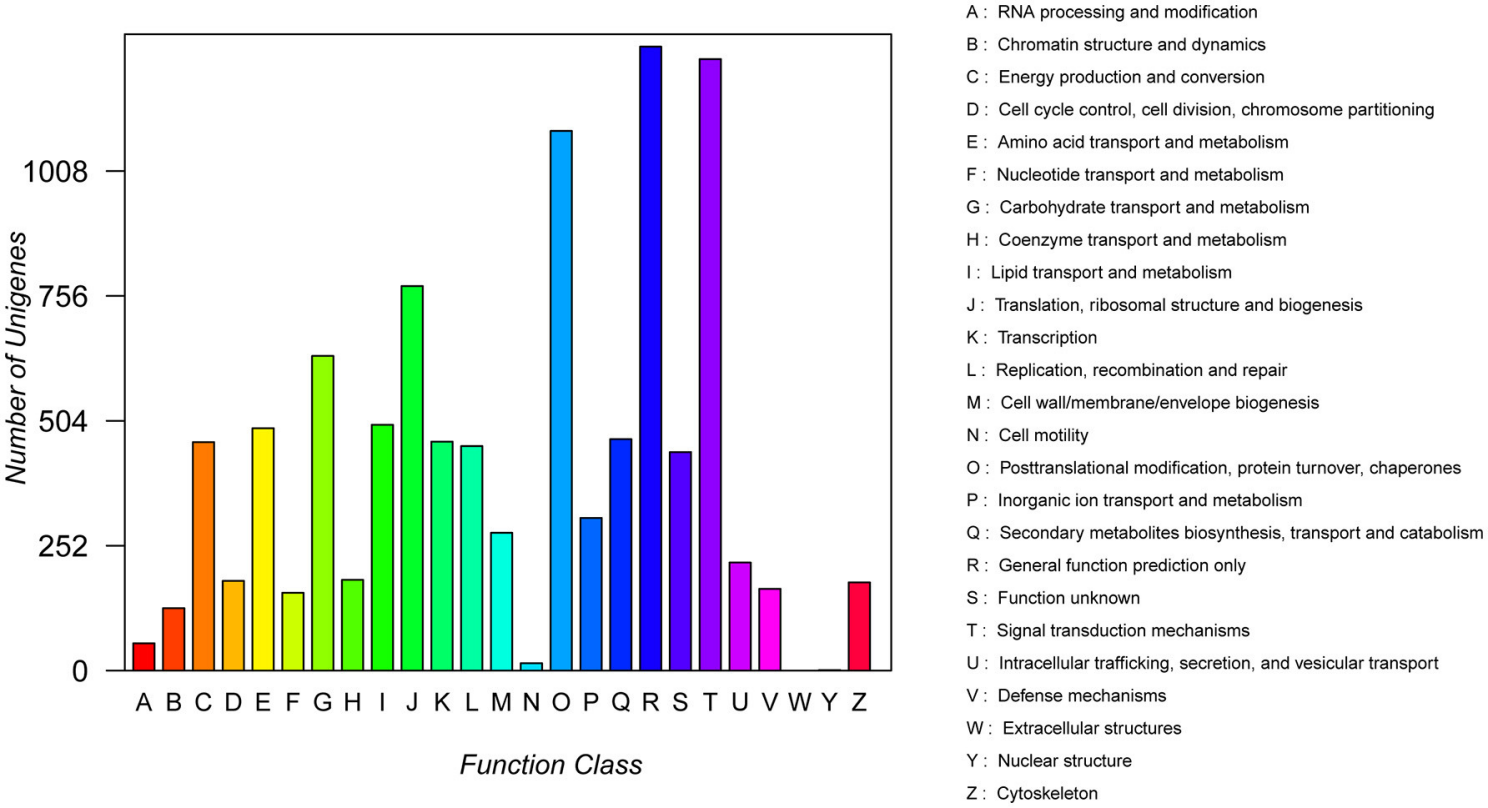
Clusters of Orthologous Groups (COG) functional classification of assembled unigenes. All 10,127 unigenes were aligned to 25 COG groups. The letters on the x-axis indicate the COG categories as listed on the right of the histogram, and the y-axis indicates the number of unigenes.

In addition, all unigenes were subjected to a search against the COG database for functional prediction and classification. 10,127 (5.46%) unigenes were assigned a COG functional classification and divided into 25 COG categories (Figure 5). Among them, the most common group was the “general function prediction only” (1,259), followed by “signal transduction” (1,234), “post-translational modification, protein turnover, chaperon” (1,089), “translation, ribosomal structure and biogenesis” (776), and “carbohydrate transport and metabolism” (635). Only a few unigenes were assigned to “extracellular structures” (0) and “nuclear structure” (1).

**Figure 6:**
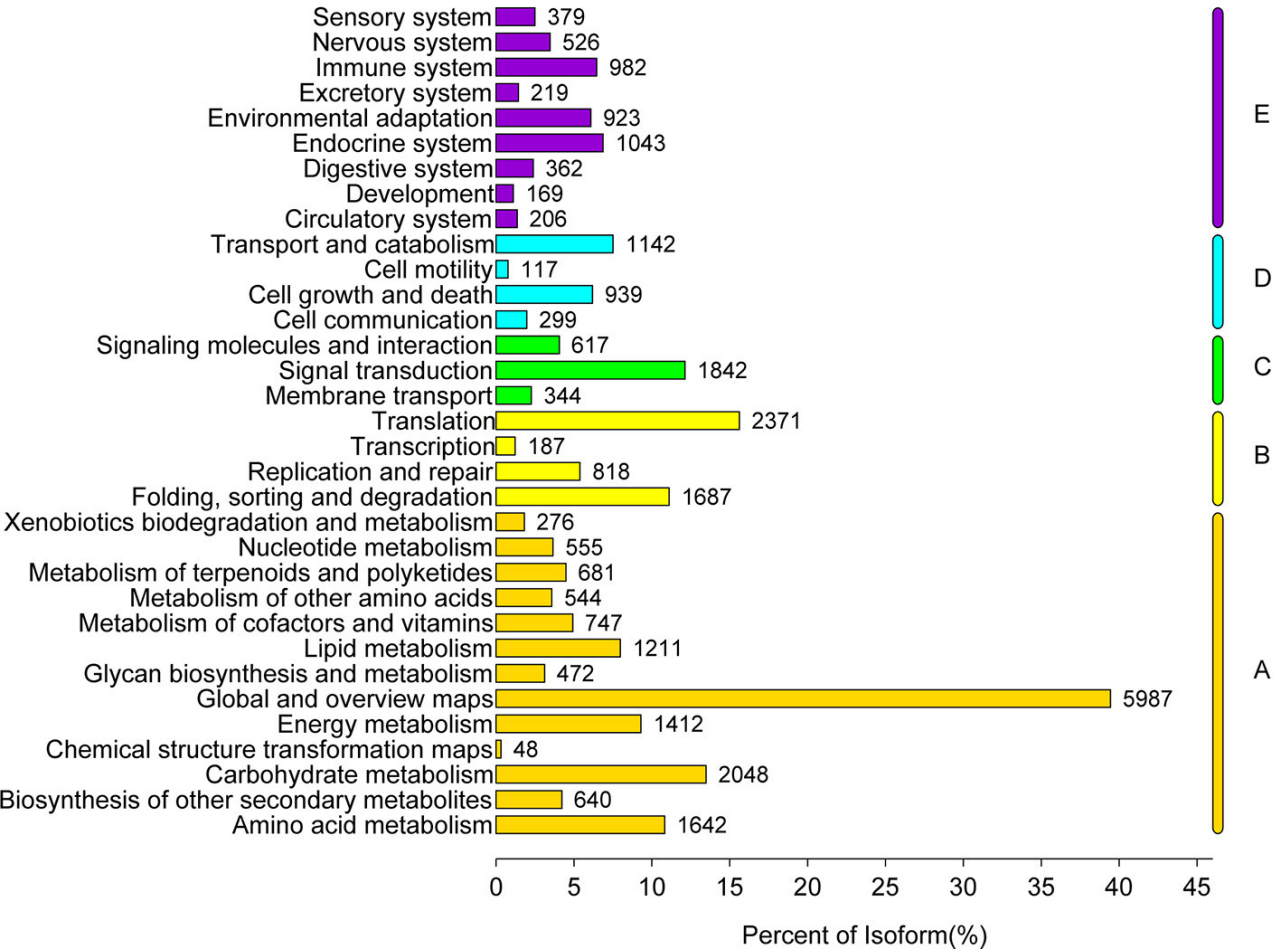
Functional classification and pathway assignment of assembled unigenes by Kyoto Encyclopedia of Genes and Genomes (KEGG). A total of 23,152 unigenes were classified to the five main KEGG metabolic pathways, they are the metabolism (A), genetic information processing (B), environmental information processing (C), cellular processes (D), and organismal systems pathways (E). The y-axis represents the name of KEGG metabolic pathway. The x-axis indicates the number of unigenes annotated to the KEGG metabolic pathway and the ratio of their number to the total number of annotated unigenes.

In a further analysis, we mapped the unigenes onto the KEGG database for categorization of gene function and identification of biochemical pathways. A total of 23,152 (12.47%) unigenes were annotated and assigned to 356 KEGG pathways. A summary of the findings is presented in Table S2. The three most highly represented pathways are “Metabolic pathways” (ko01100; 5,470), “Biosynthesis of secondary metabolites” (ko01110; 2,813) and “Microbial metabolism in diverse environments” (ko01120; 1,183). After removing the unigenes involved in the Human Diseases, the annotated unigenes were classified to five main KEGG biochemical pathways, which were associated with the metabolism, genetic information processing, environmental information processing, cellular processes and organismal systems pathways as presented in Figure 6.

### High expression and differential expression analysis of unigenes

To identify different expression levels of unigenes between these samples, we calculated the FPKM values of assembled unigenes. We identified 154 unigenes in the Light, 49 in Dark, 125 in Light-MeJA-4h and 57 in Dark-MeJA-4h with an FPKM value >1,000, respectively. Among them, 19 were found in all the four samples (Tables S3–S6). The expression level of ribulose bisphosphate carboxylase (c108280_g1) involved in photosynthesis was highest in all four samples.

Differential expression analyses were performed between Light and Dark, Light and Light-MeJA-4h, Dark and Dark-MeJA-4h, Light-MeJA-4h, and Dark-MeJA-4h of *A. annua*. DEGs [|log_2_(fold change)| ≥ 1 and FDR ≤ 0.05] were defined as unigenes that were significantly enriched or depleted in one sample relative to the other. Totally, we found 13,891 DEGs between Light and Dark, 2,145 between Light and Light-MeJA-4h, 5,456 between Dark and Dark-MeJA-4h and 10,717 between Light-MeJA-4h and Dark-MeJA-4h, respectively. A volcano plot was constructed to illustrate the distribution of DEGs in these four comparisons (Figure S2).

To further understanding of the biological functions of the DEGs, they were annotated with GO (Figures S3–S6) and enrichment analyses based on KEGG pathways were performed (Tables S7–S10; Figures S7–S10). In GO analysis, there were 1573 up-regulated DEGs and 2940 down-regulated DEGs between Dark and Light. Furthermore, there were 303 up-regulated DEGs and 182 down-regulated DEGs between Light-MeJA-4h and Light, 820 up-regulated DEGs and 236 down-regulated DEGs between Dark-MeJA-4h and Dark and 1464 up-regulated DEGs and 2341 down-regulated DEGs between Light-MeJA-4h and Dark-MeJA-4h (Figures S3–S6). When all the DEGs were checked against the KEGG pathways database, the DEGs between Light and Dark, Light and Light-MeJA-4h, Dark and Dark-MeJA-4h, Light-MeJA-4h, and Dark-MeJA-4h were linked to 251, 170, 200, 254 KEGG pathways, respectively and the pathway of “Ribosome” (ko03010), “Oxidative phosphorylation” (ko00190), “Plant-pathogen interaction” (ko04626), “Biosynthesis of amino acids” (ko01230) contain the largest number of DEGs between Light and Dark, Light and Light-MeJA-4h, Dark and Dark-MeJA-4h, Light-MeJA-4h and Dark-MeJA-4h, respectively (Tables S7–S10). In the metabolism class of KEGG pathways enrichment analysis, the metabolism pathway of “Photosynthesis-antenna proteins” (ko00196), “Diterpenoid biosynthesis” (ko00904), “Biosynthesis of ansamycins” (ko01051), “Biosynthesis of ansamycins” (ko01051) contain the most percentage of DEGs between Light and Dark, Light and Light-MeJA-4h, Dark and Dark-MeJA-4h, Light-MeJA-4h and Dark-MeJA-4h, respectively (Figures S7–S10).

### Identification of TF families and validation of DEGs by qRT-PCR

**Figure 7:**
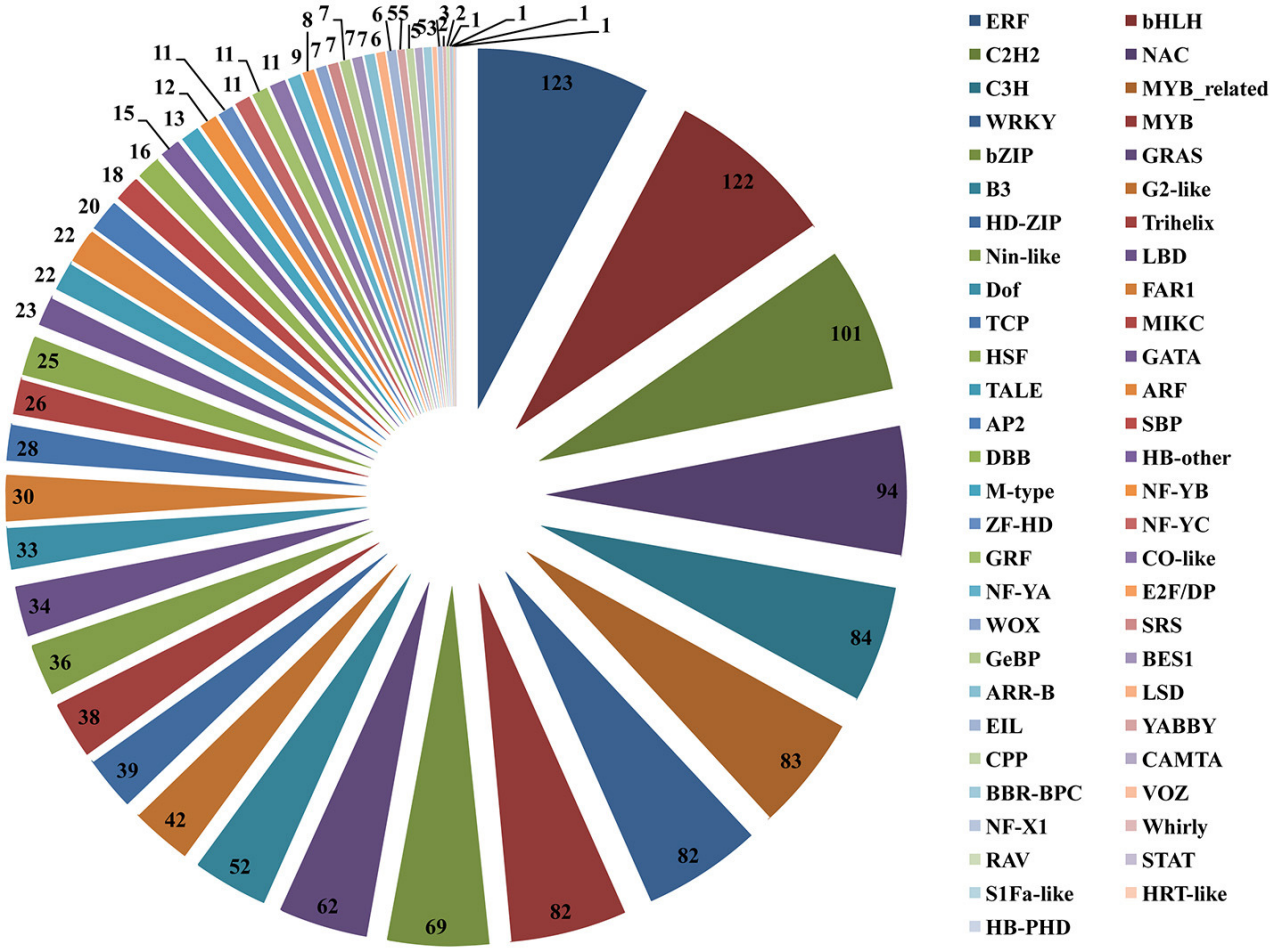
Distribution of transcription factor (TF) families. The right of the pie chart is the name of the all 55 annotated TF families. The number in the pie chart is the members of unigenes divided into each TF families.

To better survey the transcriptional regulation mechanism of artemisinin by the crosstalk between the light and JA signal, it is important to identify the differentially expressed TFs between two different samples. Firstly, a total of 1588 TFs were identified and divided into 55 TF families when aligning the annotated *A. annua* unigenes to the PlantTFDB database (Figure 7). Members of the ERF, bHLH, C2H2, NAC, C3H, MYB-related, WRKY, MYB, bZIP, and GRAS families were the top 10 classes, each with more than 62 unigenes and the ERF TF family was the largest family containing 123 members. However, members of the NF-X1, VOZ, RAV, Whirly, HB-PHD, HRT-like, S1Fa-like and STAT families have <4 unigenes.

**Figure 8:**
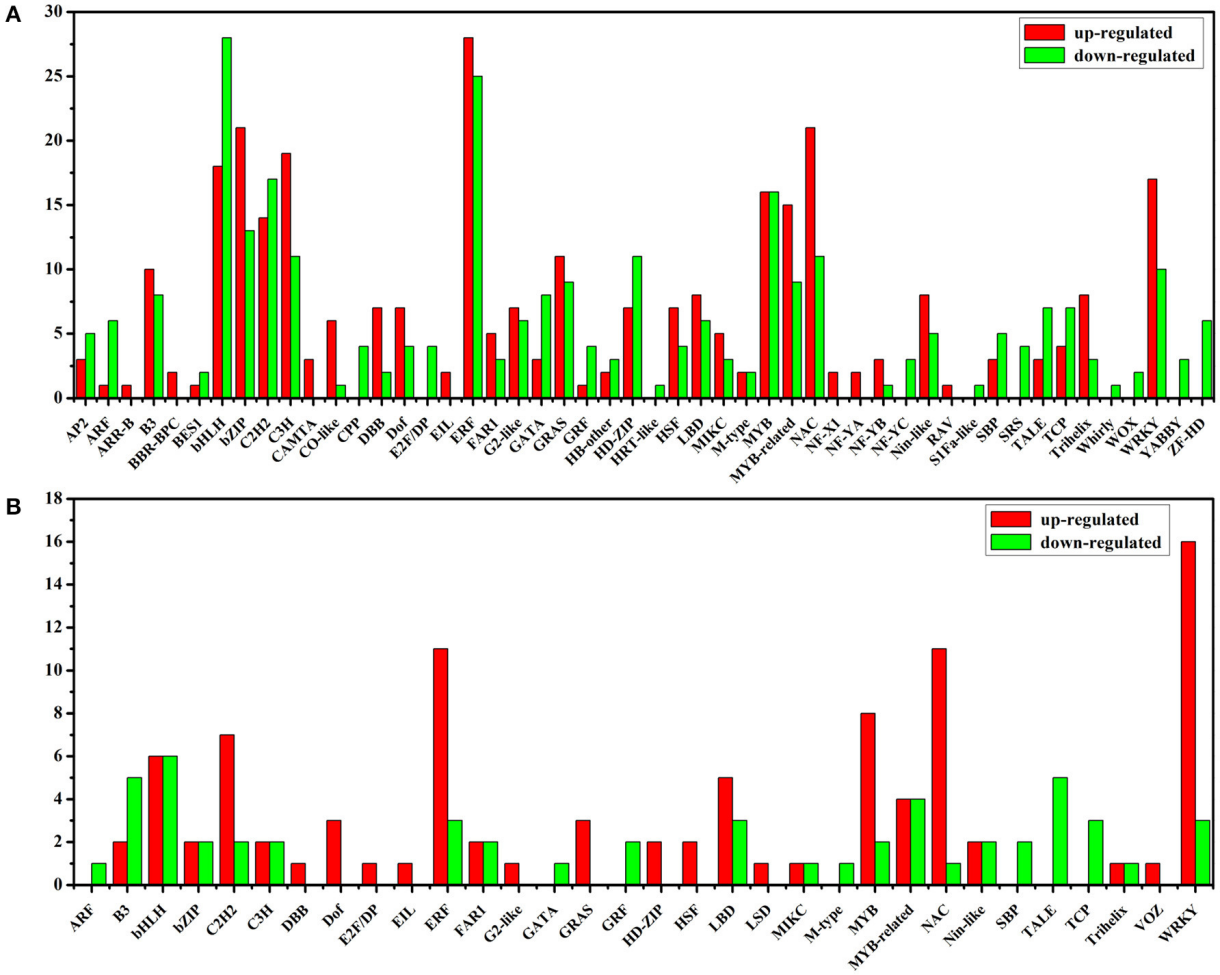
Up-regulated and down-regulated TFs between the Dark relative to Light **(A)** and Light-MeJA-4h relative to Light **(B)**. The x-axis represents the name of TF families which contain differentially expressed TFs.

After sorting all the TFs, we need to further analyze differentially expressed TFs between Light and Dark, Light and Light-MeJA-4h, Dark and Dark-MeJA-4h, Light-MeJA-4h, and Dark-MeJA-4h. In our study, we found that totally 588 TFs were influenced by the light, 284 TFs were down-regulated and 304 TFs were up-regulated in the Dark relative to Light, mainly containing bHLH, ERF, C2H2, MYB, and bZIP families. A total of 96 TFs were up-regulated and 54 TFs were down-regulated in the Light-MeJA-4h relative to Light, mainly from the WRKY, ERF, NAC, MYB and C2H2 families, thus total 150 TFs were affected under the MeJA treatment (Figure 8). We also found 298 TFs affected by the MeJA but independent of the light by comparing the Dark and Dark-MeJA-4h, which contain 242 up-regulated TFs and 56 down-regulated TFs (Figure S11). After comparing the Light-MeJA-4h and Dark-MeJA-4h, we found that totally 598 TFs were influenced by the MeJA but dependent of the light, with 367 TFs up-regulated and 231TFs down-regulated (Figure S12).

**Figure 9:**
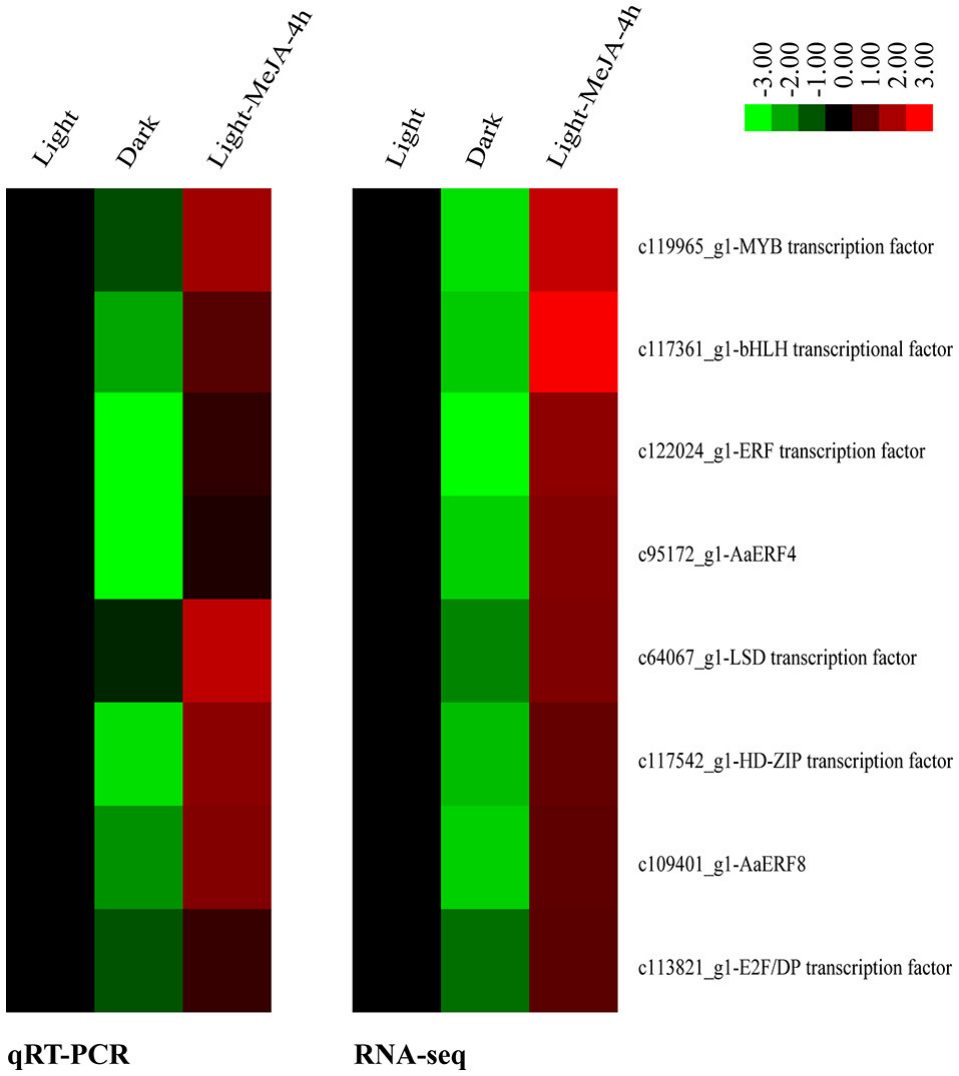
Relative heatmaps of qRT-PCR and RNA-sequencing (RNA-seq). Technical validation of 8 selected differentially expressed TFs detected by RNA-seq using qRT-PCR. Rows represent differentially expressed TFs, columns represent contrast groups. Green and red boxes represent TFs showing lower and higher expression level, respectively. Color saturation reflects magnitude of log_2_ expression ratio for each unigenes.

As described above, the expression of all the artemisinin biosynthetic genes detected were increased under the MeJA treatment in the light and decreased in the dark relative to the light. Thus, we explored down-regulated TFs between Dark relative to Light and up-regulated TFs between Light-MeJA-4h relative to Light. After crossing these two comparisons, we selected 9 TFs which increased under the MeJA treatment and decreased in the dark relative to the light. Then we confirmed these 9 TFs in the comparison of the Dark-MeJA-4h relative to Light-MeJA-4h and we found 8 of them were down-regulated, thus these 8 TFs were potentially candidate TFs which could connect the MeJA signaling with the artemisinin biosynthesis by a way of light-dependent. The selected 8 candidate TFs contained three of ERF TF families, one MYB, bHLH, HD-ZIP, LSD, and E2F/DP TF family (Figure 9).

To verify the expression of 8 selected differentially expressed TFs identified from the transcriptome sequencing, we performed qRT-PCR analyses using three samples as that used for transcriptome sequencing, namely, Light, Dark and Light-MeJA-4h. The expression patterns of these 8 selected TFs showed the same trends as in the transcriptome sequencing (Figure 9). We also analyzed the correlations of these genes and found a significant positive correlation between them, with the relatively high correlation coefficient (*R*^2^ > 0.8). These results suggested that the expression levels of these selected TFs were basically consistent with transcriptome sequencing results.

### Validation of candidate TFs involved in artemisinin metabolism regulation

**Figure 10:**
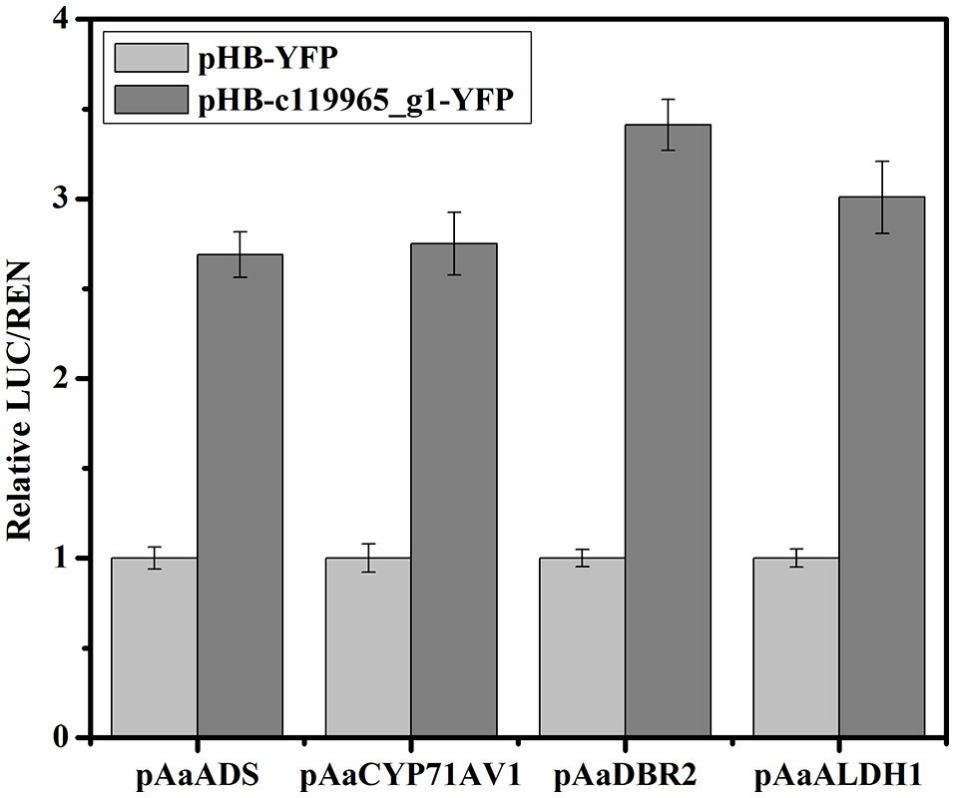
Validation of the activation of artemisinin biosynthetic genes by candidate TF using transient Dual-Luciferase (Dual-LUC) assay in *Nicotiana benthamiana*. The pHB-c119965_g1-YFP was used as effector and the pHB-YFP was used as negative controls. *pAaADS, pAaCYP71AV1, pAaDBR2* and *pAaALDH1* were fused to the luciferase reporter to get the reporter constructs and 35S promoter-driven renilla was used as the internal reference. Data are given as means ± SD (*n* = 3)

To verify if these selected TFs could activate the expression of artemisinin biosynthetic genes *AaADS, AaCYP71AV1, AaDBR2*, and *AaALDH1*, we chose the MYB TF (c119965_g1) and performed the Dual-LUC assay in tobacco leaf. Based on the transcriptome analysis, we obtained the full-length cDNAs of c119965_g1 and inserted it into pHB-YFP construct. Four reporter constructs were produced by inserting the promoter of *AaADS, AaCYP71AV1, AaDBR2* and *AaALDH1* into the pGreenII 0800-LUC vector, respectively. Sets of effector and reporter constructs were co-infiltrated into tobacco leaves. As shown in the Figure 10, c119965_g1 activated the expression of *AaADS, AaCYP71AV1, AaDBR2*, and *AaALDH1* promoters and showed the higher value of LUC/REN than the YFP control (about 2.7–3.4 folds). Together, these results showed that c119965_g1 is a strong candidate TF with ability to transactivate the *AaADS, AaCYP71AV1, AaDBR2*, and *AaALDH1* genes *in vivo*.

## Discussion

### The roles of phytohormone on the artemisinin is dependent on the light

Some phytohormones, such as JA (Wang et al., 2010; Maes et al., 2011), abscisic acid (ABA) (Jing et al., 2009), salicylic acid (SA) (Pu et al., 2009), and gibberellins (GA_3_) (Zhang et al., 2005) are key factors that are involved in positively affecting artemisinin production in *A. annua*. In these phytohormones, JA could significantly promote the glandular trichome formation and activate the expressions of the artemisinin biosynthetic genes. The consequence is that JA could increase the accumulation of the artemisinin in both the high-artemisinin producer and low-artemisinin producer cultivars (Maes et al., 2011). In our study, we found that expression of the related genes increased under the JA treatment in the light. But the transcriptional regulation mechanism is not clear. In recent years, there have been characterized several TFs which could directly bind to the promoters of the aretmisinin biosynthetic genes and regulate the artemisinin content in *A. annua* (Ma et al., 2009; Yu et al., 2012; Lu et al., 2013a; Shen et al., 2016). Furthermore, a novel TF, AaHD1 (HOMEODOMAIN PROTEIN 1), which belongs to the homeodomain-leucine zipper TF family was identified by our lab (Yan et al., 2016). The AaHD1 played an important role in promoting the JA-mediated glandular trichome initiation and thus improved the artemisinin content in *A. annua* (Yan et al., 2016). For the ABA signaling to artemisinin accumulation in *A. annua*, the AabZIP1, a basic leucine zipper TF, was identified to activate the expression of *AaADS* and *AaCYP71AV1* and promote artemisinin biosynthesis (Zhang et al., 2015). Besides, the AabHLH1 was strongly induced by ABA and could bind to the promoters of the *AaADS* and *AaCYP71AV1* which positively regulated the biosynthesis of artemisinin (Ji et al., 2014). Thus, there are only a few identified and characterized TFs which connect the phytohormone and the artemisinin accumulation in *A. annua*. So we need to find other TFs involved in the phytohormone signaling to the artemisinin biosynthesis using transcriptome sequencing technology.

In addition to planthormones, some other environmental factors can also affect artemisinin content in the *A. annua*, like the light, low temperature, salinity, drought and heavy metals (Wang et al., 2001; Guo et al., 2003; Qureshi et al., 2005; Qian et al., 2007). Previous reports have also shown that ultraviolet (UV) light significantly induced the expression of key genes in artemisinin biosynthesis and led to an increase in the concentration of artemisinin (Rai et al., 2011; Pandey and Pandey-Rai, 2014; Pan et al., 2014). In 2014, Pan et al. reported that UV-B treatment not only stimulated the biosynthesis of artemisinin, but also induced the generation of reactive oxygen species (ROS), enhanced peroxidase activity and endogenous content of abscisic acid (ABA) in the seedlings (Pan et al., 2014). Thus, they detected the transcriptomic changes during UV-B radiation (wavelength range 280–320 nm) in *A. annua* using an Agilent GeneChip with 43,692 probe sets and they found that the expression of *AaADS, AaCYP71AV1*, and related WRKY TFs was up-regulated significantly for artemisinin biosynthesis (Pan et al., 2014). In our study, we also found that the expression of artemisinin biosynthetic genes was influenced by the light and they all reduced in the dark for 24 h relative to the expression level under the light. Although it is reported that totally 358 transcripts were identified as DEGs under UV-B stress, of which 172 transcripts increased and 186 transcripts decreased in abundance, but we think that it is essential to detect other EDGs which may be involved in the artemisinin biosynthesis under the light (contain the UV-B) treatment using the transcriptome sequencing technology.

As we had found that the expression of the artemisinin biosynthetic genes could be affected by the light and the JA treatment, we still wanted to know if there is a crosstalk of these two treatments. After treating the *A. annua* seedlings using the MeJA in the light and dark, we found that the increasing of the JA treatment was dependent on the light. It meant that the expressions of the artemisinin biosynthetic genes were increased under the MeJA treatment in the light rather than in the dark. As some other plant hormones also could affect the artemisinin in the light, so we concluded that they might have the same regulation mechanism with the JA. Therefore, we explored the relationship between the artemisinin biosynthesis and the MeJA treatment under the light using the transcriptome sequencing technology.

### Illumina sequencing of *A. annua*

Transcriptome sequencing is a powerful tool for comprehensive analyses of non-model plants in transcriptional levels currently. *A. annua* is a famous traditional Chinese medicinal plant which is the main source of the antimalarial drug artemisinin. There are some reports on transcript profiling and transcriptome sequencing of the *A. annua* before (Wang et al., 2009; Graham et al., 2010; Maes et al., 2011; Soetaert et al., 2013; Pan et al., 2014). In our study, we found that the improvement of the MeJA to the artemisinin biosynthesis is dependent on the light. So we describe a global view of gene expression dynamics under the MeJA and light treatment and it is the attempt using Illumina sequencing technology to gain an insight into the transcriptome of different treatment in *A. annua*. After transcriptome sequencing of selected four samples, we obtained about 267 million clean data which were *de novo* assembled into 260,093 transcripts and 185,653 unigenes. Among the assembled unigenes, 59,490 unigenes (32.04%) could be functionally annotated against five public protein databases (NR, Pfam, Swiss-Prot, String and KEGG). Furthermore, about 126,163 unigenes (67.96%) were not annotated to any pubic protein database. As these non-functional annotated unigenes could match to proteins of unknown function or could not be matched to any proteins, these unigenes may contain unique genes for *A. annua* and have value of future research. Thus, our transcriptome data could meet the initial information requirements for following differential expression analysis and transcriptional regulation analysis of different treatment in *A. annua*.

After we performed the transcriptome assembly, functional annotation and classification of unigenes, we further analyze the expression levels of unigenes in the Light, Dark, Light-MeJA-4h, and Dark-MeJA-4h of *A. annua* seedling to profile global gene expression of these four samples. Firstly, we analyzed the highly expressed unigenes and identified 154 unigenes in the Light, 49, 125, and 57 in the Dark, Light-MeJA-4h and Dark-MeJA-4h with an FPKM value >1,000, respectively. The highly expressed unigenes in the Light and Light-MeJA-4h are apparently more than the Dark and Dark-MeJA-4h, so it is reasonable that the light is an important environment factors and it could play a crucial role in the process of plant growth and development. Then, differential expression analysis was performed between Light and Dark, Light and Light-MeJA-4h, Dark and Dark-MeJA-4h, Light-MeJA-4h, and Dark-MeJA-4h. Totally, we found 13,891 DEGs between Light and Dark, 2,145 between Light and Light-MeJA-4h, 5,456 between Dark and Dark-MeJA-4h, and 10,717 between Light-MeJA-4h and Dark-MeJA-4h, respectively. More DEGs between Light and Dark were discovered than in other three comparisons, and that is reasonable when taking into account that light affects large amounts of progresses in plants. Thus, we have got massive differential expression data of different samples and these data are valuable for us to further analyze the differential expression of TFs.

To identify the TFs that could be involved in the artemisinin biosynthesis, we further analyze the TFs and their differential expression profile. In our study, a total of 1588 TFs were identified and divided into 55 TF families (Figure 7). After analyzing differential expression profile, we found that 284 TFs were down-regulated in the Dark relative to Light and 96 TFs up-regulated in the Light-MeJA-4h relative to Light (Figure 8). After taking the intersection of TFs in these two comparisons and verifying in the comparison of the Dark-MeJA-4h relative to Light-MeJA-4h, we found 8 TFs that belonged to the bHLH, MYB, ERF, LSD, HD-ZIP, E2F/DP family TFs. Thus, we screened 8 TFs which have the potential to regulate the artemisinin biosynthesis and we need to further verify their function.

### Identification of TFs in the regulation of the artemisinin biosynthesis

JA could promote artemisinin accumulation in the light. However, the regulation mechanisms of artemisinin are rarely understood. TFs play an important role in regulating plant growth and development (Yang et al., 2012). Transcriptome sequencing have been used to analyze and identify various TF families in medicinal plants in recent years, such as *Atractylodes lancea* (Huang et al., 2016), *Bupleurum chinense* (Sui et al., 2015), and *Salvia miltiorrhiza* (Gao et al., 2014). After analyzing the differential expression of TFs in our transcriptome, we have selected 8 TFs which have the potential to regulate the artemisinin biosynthesis. Firstly, we confirmed their expression using qRT-PCR and the expression was consistent with the transcriptome sequencing data. Then, we selected one of them and verified the activation to the artemisinin biosynthetic genes using Dual-LUC assay, observing that the selected MYB TF could positively increase the expression of the artemisinin biosynthetic genes. So the screening method using the transcriptome sequencing is of value and these selected 8 TFs mentioned above may be the candidates for the crosstalk regulation of artemisinin biosynthesis by the light and JA signaling.

The MYB TFs are one of the largest TF families in plants and have function in diverse biological processes, such as the regulation of primary and secondary metabolism, hormone signal transduction, defense, and stress response (Feller et al., 2011). The common feature of all MYB TFs is the presence of one to four or more imperfect MYB repeats (R), which can function synergistically or individually in DNA binding and protein–protein interactions, respectively. The functions of R2R3-MYB proteins are primarily related to regulation of plant formation and plant metabolism. After validation of the candidate MYB TF (R2R3-MYB TF) which was involved in artemisinin metabolism regulation by Dual-LUC assay, we observed that the MYB TF (c119965_g1) could improve the expression of all the artemisinin biosynthetic genes (Figure 10). The transcriptional regulation mechanism of the crosstalk between light and JA signaling mediated by this R2R3-MYB TF will be further studied.

In addition to the MYB TFs, we also selected three ERF TFs. APETALA2/Ethylene Responsive Factor (AP2/ERF) superfamily composed of ERF family, AP2 family, RAV family and Soloist is conservatively widespread in plants (Licausi et al., 2013). As a part of the AP2/ERF superfamily, the ERF family is a large gene family and involved in the regulation of metabolites biosynthesis, developmental processes and responses to environmental stimuli in plants (Nakano et al., 2006). As reported before, there are four ERF TFs could regulate the artemisinin biosynthesis, namely AaERF1, AaERF2, AaORA, and AaTAR1 (Yu et al., 2012; Lu et al., 2013a,b; Tan et al., 2015). In our study, we have found 123 ERF TFs which were the largest TF family in our transcriptome. Furthermore, the expression level of the selected three ERF TFs increased after the MeJA treatment under the light and decreased after 24 h dark treatment, the same with the expression level of the artemisinin biosynthetic genes. So these three ERF TFs have the potential to regulate the artemisinin biosynthesis.

Similar with the ERF family, the bHLH family was also a largest family, only behind the ERF family in our study. After screened using the transcriptome sequencing, we also found a bHLH TF involved in the regulation of artemisinin biosynthesis. The bHLH TFs are reportedly involved in the developmental process, metabolic regulation, light signaling and various abiotic stress (Toledo-Ortiz, 2003). As reported before, two bHLH TFs, the AabHLH1 and AaMYC2, could positively regulate the accumulation of artemisinin (Ji et al., 2014; Shen et al., 2016). So we need to further analyze the selected bHLH TF.

Furthermore, other three selected TFs respectively belong to the HD-ZIP, LSD, and E2F/DP TF families. The HD-ZIP family is unique to the plants and could participate in organ development and response to environmental conditions (Ariel et al., 2007). The HD-ZIP TFs contain a homedomain with a leucine zipper motif and can be classified into four subfamilies (Ariel et al., 2007). AaHD1 belongs to the HD-ZIP IV subfamily and could promote glandular trichome initiation and improve the artemisinin content (Yan et al., 2016). In our study, the family of HD-ZIP, LSD and E2F/DP contains 39, 6 and 8 proteins, respectively, it is necessary to further analyze their function in *A. annua*. So these selected eight TFs have the potential to regulate gene expression in the artemisinin biosynthesis and need to be further studied.

## Conclusions

In this study, 266.7 million clean data were assembled into 185,653 unigenes, including 59,490 annotated unigenes. Differential expression analyses were performed and 13,891, 2,145, 5,456, 10,717 DEGs were identified between Light and Dark, Light and Light-MeJA-4h, Dark and Dark-MeJA-4h, Light-MeJA-4h and Dark-MeJA-4h, respectively. In addition, TF analyses revealed that 1588 TFs were identified and 8 TFs were selected as candidates for regulating the artemisinin biosynthesis. The transcriptome sequencing data reported in this study will facilitate future research on transcriptional regulation mechanisms in *A. annua* and other traditional Chinese medicinal plants.

## Author contributions

XH, LL, and KT conceived and designed the project. XH, YZ, XF, ZL, and PS performed the experiments. XH, QS, TY, YM and MC analyzed the data. XH, XL, and ZW wrote the manuscript. JZ, XS, LL, and KT revised the manuscript. All authors read and approved the final manuscript.

### Conflict of interest statement

The authors declare that the research was conducted in the absence of any commercial or financial relationships that could be construed as a potential conflict of interest.
