# Supplementary material for: Transcriptome Analysis of Genes Associated with the Artemisinin Biosynthesis by Jasmonic Acid Treatment under the Light in Artemisia annua
Source: Front Plant Sci. 2017 Jun 8;8:971. doi: 10.3389/fpls.2017.00971 (PMC5463050; doi:10.3389/fpls.2017.00971)
Supplement: Supplementary file 1 [file Table1.PDF]

Table S1 Primers of genes used for qRT-PCR analysis in this article.

Primer	Sequence(5'-3')
Actin-qRT-F	CCAGGCTGTTCAGTCTCTGTAT
Actin-qRT-R	CGCTCGGTAAGGATCTTCATCA
ADS-qRT-F	AATGGGCAAATGAGGGACAC
ADS-qRT-R	TTTCAAGGCTCGATGAACTATG
CYP71AV1-qRT-F	CACCCTCCACTACCCTTG
CYP71AV1-qRT-R	GACACATCCTTCTCCCAGC
DBR2-qRT-F	CTTGGGTTACAAGCTGTGGCTCAAG
DBR2-qRT-R	ATATAATCAAAACTAGAGGAGTGACC
ALDH1-qRT-F	CAGTTTCTGACCCAAATCCAGGTTGA
ALDH1-qRT-R	TCGGAGTAGTTGGTCACAT
c119965_g1-qRT-F	TATCTACGACCCGGTATCAAGAG
c119965_g1-qRT-R	TGAGGTGGGCGTTCCAATAGTT
c117361_g1-qRT-F	TTGGAACAACAGACAAGGTGGAAG
c117361_g1-qRT-R	AGGGCGGGCTTTGGAGGTATGAG
c122024_g1-qRT-F	CCGAATACTAAAGCGGGTAAAC
c122024_g1-qRT-R	TCGGATTTCGGATACCCATTTGC
c95172_g1-qRT-F	TGGCTAGGCACATTTGATACCGC
c95172_g1-qRT-R	CAGCCTTCTCAGTATTCGTCCTT
c64067_g1-qRT-F	TCCAACTGTAATATAACATTAAC
c64067_g1-qRT-R	CGCCTGCTGGCTGAAACCTGACT
c117542_g1-qRT-F	ATCAAACGGTCACTGTAAGGTCAC
c117542_g1-qRT-R	AGGAACATTTGTTGCGAGGACG
c109401_g1-qRT-F	ACAACAACAACGACGACAACAAC
c109401_g1-qRT-R	CATCATAAGCAGCAGCAGCCATCG
c113821_g1-qRT-F	TTGGTATTCAGGGTTCGGCTTAT
c113821_g1-qRT-R	CTTGTGAGCCATAACCAACGATT
