# Supplementary material for: Transcriptome Analysis of Genes Associated with the Artemisinin Biosynthesis by Jasmonic Acid Treatment under the Light in Artemisia annua
Source: Front Plant Sci. 2017 Jun 8;8:971. doi: 10.3389/fpls.2017.00971 (PMC5463050; doi:10.3389/fpls.2017.00971)
Supplement: Supplementary file 2 [file Table2.PDF]

Table S2 Summary of KEGG pathways involved in the *A. annua* transcriptome.

NO.	Pathway	Pathway Definition	Number of Sequences	Percent (%)
1	ko01100	Metabolic pathways	5470	23.626%
2	ko01110	Biosynthesis of secondary metabolites	2813	12.150%
3	ko01120	Microbial metabolism in diverse environments	1183	5.110%
4	ko01200	Carbon metabolism	895	3.866%
5	ko01230	Biosynthesis of amino acids	800	3.455%
6	ko04141	Protein processing in endoplasmic reticulum	769	3.322%
7	ko03010	Ribosome	752	3.248%
8	ko05169	Epstein-Barr virus infection	659	2.846%
9	ko03040	Spliceosome	622	2.687%
10	ko04075	Plant hormone signal transduction	597	2.579%
11	ko00500	Starch and sucrose metabolism	594	2.566%
12	ko04626	Plant-pathogen interaction	572	2.471%
13	ko04144	Endocytosis	523	2.259%
14	ko05145	Toxoplasmosis	509	2.199%
15	ko03013	RNA transport	507	2.190%
16	ko05164	Influenza A	487	2.103%
17	ko05152	Tuberculosis	445	1.922%
18	ko05162	Measles	440	1.900%
19	ko00190	Oxidative phosphorylation	427	1.844%
20	ko05016	Huntington's disease	426	1.840%
21	ko00010	Glycolysis / Gluconeogenesis	421	1.818%
22	ko00940	Phenylpropanoid biosynthesis	417	1.801%
23	ko05203	Viral carcinogenesis	399	1.723%
24	ko04120	Ubiquitin mediated proteolysis	393	1.697%
25	ko00230	Purine metabolism	388	1.676%
26	ko04722	Neurotrophin signaling pathway	368	1.589%
27	ko01212	Fatty acid metabolism	363	1.568%
28	ko05134	Legionellosis	357	1.542%
29	ko05010	Alzheimer's disease	347	1.499%
30	ko04151	PI3K-Akt signaling pathway	341	1.473%
31	ko00620	Pyruvate metabolism	337	1.456%
32	ko04612	Antigen processing and presentation	325	1.404%
33	ko03018	RNA degradation	324	1.399%
34	ko00970	Aminoacyl-tRNA biosynthesis	316	1.365%
35	ko00520	Amino sugar and nucleotide sugar metabolism	314	1.356%
36	ko03015	mRNA surveillance pathway	314	1.356%
37	ko04915	Estrogen signaling pathway	313	1.352%
38	ko04110	Cell cycle	305	1.317%
39	ko04152	AMPK signaling pathway	299	1.291%
40	ko05142	Chagas disease (American trypanosomiasis)	298	1.287%

41	ko04114	Oocyte meiosis	297	1.283%
42	ko05133	Pertussis	294	1.270%
43	ko00270	Cysteine and methionine metabolism	285	1.231%
44	ko00240	Pyrimidine metabolism	280	1.209%
45	ko05012	Parkinson's disease	275	1.188%
46	ko04010	MAPK signaling pathway	275	1.188%
47	ko04620	Toll-like receptor signaling pathway	273	1.179%
48	ko05166	HTLV-I infection	270	1.166%
49	ko00710	Carbon fixation in photosynthetic organisms	268	1.158%
50	ko04910	Insulin signaling pathway	268	1.158%
51	ko00260	Glycine, serine and threonine metabolism	263	1.136%
52	ko04145	Phagosome	257	1.110%
53	ko03030	DNA replication	256	1.106%
54	ko05140	Leishmaniasis	254	1.097%
55	ko00360	Phenylalanine metabolism	248	1.071%
56	ko00564	Glycerophospholipid metabolism	246	1.063%
57	ko00680	Methane metabolism	241	1.041%
58	ko05200	Pathways in cancer	241	1.041%
59	ko04111	Cell cycle - yeast	237	1.024%
60	ko03008	Ribosome biogenesis in eukaryotes	237	1.024%
61	ko04146	Peroxisome	235	1.015%
62	ko04932	Non-alcoholic fatty liver disease (NAFLD)	231	0.998%
63	ko04210	Apoptosis	230	0.993%
64	ko00630	Glyoxylate and dicarboxylate metabolism	229	0.989%
65	ko05168	Herpes simplex infection	227	0.980%
66	ko04064	NF-kappa B signaling pathway	227	0.980%
67	ko00480	Glutathione metabolism	227	0.980%
68	ko00330	Arginine and proline metabolism	219	0.946%
69	ko02010	ABC transporters	217	0.937%
70	ko00040	Pentose and glucuronate interconversions	215	0.929%
71	ko04068	FoxO signaling pathway	215	0.929%
72	ko00052	Galactose metabolism	211	0.911%
73	ko00561	Glycerolipid metabolism	211	0.911%
74	ko04024	cAMP signaling pathway	211	0.911%
75	ko04666	Fc gamma R-mediated phagocytosis	210	0.907%
76	ko05034	Alcoholism	208	0.898%
77	ko05206	MicroRNAs in cancer	205	0.885%
78	ko04810	Regulation of actin cytoskeleton	205	0.885%
79	ko04142	Lysosome	203	0.877%
80	ko04066	HIF-1 signaling pathway	200	0.864%
81	ko00020	Citrate cycle (TCA cycle)	197	0.851%
82	ko04921	Oxytocin signaling pathway	192	0.829%
83	ko03050	Proteasome	189	0.816%
84	ko04721	Synaptic vesicle cycle	186	0.803%

85	ko04919	Thyroid hormone signaling pathway	183	0.790%
86	ko00051	Fructose and mannose metabolism	182	0.786%
87	ko04310	Wnt signaling pathway	181	0.782%
88	ko01210	2-Oxocarboxylic acid metabolism	180	0.777%
89	ko04113	Meiosis - yeast	179	0.773%
90	ko00061	Fatty acid biosynthesis	178	0.769%
91	ko00071	Fatty acid degradation	176	0.760%
92	ko00860	Porphyrin and chlorophyll metabolism	173	0.747%
93	ko05205	Proteoglycans in cancer	172	0.743%
94	ko05215	Prostate cancer	171	0.739%
95	ko03320	PPAR signaling pathway	170	0.734%
96	ko00900	Terpenoid backbone biosynthesis	169	0.730%
97	ko01040	Biosynthesis of unsaturated fatty acids	167	0.721%
98	ko04014	Ras signaling pathway	167	0.721%
99	ko00030	Pentose phosphate pathway	165	0.713%
100	ko04022	cGMP-PKG signaling pathway	163	0.704%
101	ko00460	Cyanoamino acid metabolism	162	0.700%
102	ko00720	Carbon fixation pathways in prokaryotes	162	0.700%
103	ko00592	alpha-Linolenic acid metabolism	157	0.678%
104	ko00562	Inositol phosphate metabolism	155	0.669%
105	ko04070	Phosphatidylinositol signaling system	154	0.665%
106	ko04261	Adrenergic signaling in cardiomyocytes	153	0.661%
107	ko00280	Valine, leucine and isoleucine degradation	153	0.661%
108	ko04724	Glutamatergic synapse	150	0.648%
109	ko04350	TGF-beta signaling pathway	147	0.635%
110	ko03420	Nucleotide excision repair	147	0.635%
111	ko04712	Circadian rhythm - plant	145	0.626%
112	ko00250	Alanine, aspartate and glutamate metabolism	142	0.613%
113	ko00941	Flavonoid biosynthesis	138	0.596%
114	ko00982	Drug metabolism - cytochrome P450	137	0.592%
115	ko00400	Phenylalanine, tyrosine and tryptophan biosynthesis	136	0.587%
116	ko04728	Dopaminergic synapse	135	0.583%
117	ko04914	Progesterone-mediated oocyte maturation	135	0.583%
118	ko04720	Long-term potentiation	134	0.579%
119	ko03060	Protein export	132	0.570%
120	ko04150	mTOR signaling pathway	132	0.570%
121	ko04912	GnRH signaling pathway	131	0.566%
122	ko00980	Metabolism of xenobiotics by cytochrome P450	128	0.553%
123	ko00410	beta-Alanine metabolism	127	0.549%
124	ko00053	Ascorbate and aldarate metabolism	126	0.544%
125	ko00195	Photosynthesis	125	0.540%
126	ko04510	Focal adhesion	125	0.540%
127	ko04976	Bile secretion	124	0.536%
128	ko00380	Tryptophan metabolism	123	0.531%

129	ko05110	Vibrio cholerae infection	123	0.531%
130	ko05161	Hepatitis B	123	0.531%
131	ko00350	Tyrosine metabolism	123	0.531%
132	ko03460	Fanconi anemia pathway	122	0.527%
133	ko05160	Hepatitis C	120	0.518%
134	ko05204	Chemical carcinogenesis	120	0.518%
135	ko03440	Homologous recombination	118	0.510%
136	ko04390	Hippo signaling pathway	118	0.510%
137	ko04360	Axon guidance	117	0.505%
138	ko02020	Two-component system	116	0.501%
139	ko04020	Calcium signaling pathway	115	0.497%
140	ko04540	Gap junction	114	0.492%
141	ko04930	Type II diabetes mellitus	111	0.479%
142	ko04520	Adherens junction	109	0.471%
143	ko05202	Transcriptional misregulation in cancer	108	0.466%
144	ko04015	Rap1 signaling pathway	107	0.462%
145	ko04916	Melanogenesis	107	0.462%
146	ko03020	RNA polymerase	106	0.458%
147	ko05132	Salmonella infection	104	0.449%
148	ko04662	B cell receptor signaling pathway	104	0.449%
149	ko05210	Colorectal cancer	104	0.449%
150	ko04621	NOD-like receptor signaling pathway	103	0.445%
151	ko05131	Shigellosis	102	0.441%
152	ko00910	Nitrogen metabolism	101	0.436%
153	ko00130	Ubiquinone and other terpenoid-quinone biosynthesis	100	0.432%
154	ko05130	Pathogenic Escherichia coli infection	100	0.432%
155	ko00909	Sesquiterpenoid and triterpenoid biosynthesis	99	0.428%
156	ko04920	Adipocytokine signaling pathway	99	0.428%
157	ko00640	Propanoate metabolism	99	0.428%
158	ko03430	Mismatch repair	97	0.419%
159	ko04710	Circadian rhythm	96	0.415%
160	ko00830	Retinol metabolism	95	0.410%
161	ko00510	N-Glycan biosynthesis	95	0.410%
162	ko00310	Lysine degradation	94	0.406%
163	ko00600	Sphingolipid metabolism	93	0.402%
164	ko04660	T cell receptor signaling pathway	92	0.397%
165	ko05214	Glioma	91	0.393%
166	ko04062	Chemokine signaling pathway	91	0.393%
167	ko05120	Epithelial cell signaling in Helicobacter pylori infection	91	0.393%
168	ko05211	Renal cell carcinoma	91	0.393%
169	ko00945	Stilbenoid, diarylheptanoid and gingerol biosynthesis	90	0.389%
170	ko04270	Vascular smooth muscle contraction	90	0.389%
171	ko00565	Ether lipid metabolism	89	0.384%
172	ko00196	Photosynthesis - antenna proteins	89	0.384%

173	ko04391	Hippo signaling pathway - fly	88	0.380%
174	ko04370	VEGF signaling pathway	88	0.380%
175	ko04140	Regulation of autophagy	88	0.380%
176	ko00920	Sulfur metabolism	88	0.380%
177	ko03410	Base excision repair	87	0.376%
178	ko04380	Osteoclast differentiation	87	0.376%
179	ko00780	Biotin metabolism	87	0.376%
180	ko00062	Fatty acid elongation	87	0.376%
181	ko04650	Natural killer cell mediated cytotoxicity	86	0.371%
182	ko04727	GABAergic synapse	85	0.367%
183	ko05213	Endometrial cancer	84	0.363%
184	ko05323	Rheumatoid arthritis	83	0.359%
185	ko05020	Prion diseases	82	0.354%
186	ko04012	ErbB signaling pathway	82	0.354%
187	ko00650	Butanoate metabolism	81	0.350%
188	ko03022	Basal transcription factors	81	0.350%
189	ko04115	p53 signaling pathway	81	0.350%
190	ko05322	Systemic lupus erythematosus	80	0.346%
191	ko04961	Endocrine and other factor-regulated calcium reabsorption	80	0.346%
192	ko04130	SNARE interactions in vesicular transport	78	0.337%
193	ko05100	Bacterial invasion of epithelial cells	77	0.333%
194	ko05014	Amyotrophic lateral sclerosis (ALS)	76	0.328%
195	ko00290	Valine, leucine and isoleucine biosynthesis	76	0.328%
196	ko00450	Selenocompound metabolism	75	0.324%
197	ko04730	Long-term depression	75	0.324%
198	ko04713	Circadian entrainment	75	0.324%
199	ko04723	Retrograde endocannabinoid signaling	74	0.320%
200	ko04530	Tight junction	74	0.320%
201	ko00100	Steroid biosynthesis	74	0.320%
202	ko04664	Fc epsilon RI signaling pathway	73	0.315%
203	ko00591	Linoleic acid metabolism	73	0.315%
204	ko05220	Chronic myeloid leukemia	73	0.315%
205	ko05212	Pancreatic cancer	73	0.315%
206	ko04918	Thyroid hormone synthesis	72	0.311%
207	ko00906	Carotenoid biosynthesis	72	0.311%
208	ko04917	Prolactin signaling pathway	70	0.302%
209	ko00770	Pantothenate and CoA biosynthesis	70	0.302%
210	ko05031	Amphetamine addiction	69	0.298%
211	ko00670	One carbon pool by folate	69	0.298%
212	ko03070	Bacterial secretion system	68	0.294%
213	ko04611	Platelet activation	67	0.289%
214	ko00340	Histidine metabolism	65	0.281%
215	ko05223	Non-small cell lung cancer	64	0.276%

216	ko04962	Vasopressin-regulated water reabsorption	64	0.276%
217	ko00960	Tropane, piperidine and pyridine alkaloid biosynthesis	64	0.276%
218	ko04966	Collecting duct acid secretion	63	0.272%
219	ko00950	Isoquinoline alkaloid biosynthesis	63	0.272%
220	ko05221	Acute myeloid leukemia	63	0.272%
221	ko04972	Pancreatic secretion	62	0.268%
222	ko05218	Melanoma	59	0.255%
223	ko04668	TNF signaling pathway	59	0.255%
224	ko00511	Other glycan degradation	59	0.255%
225	ko04112	Cell cycle - Caulobacter	58	0.251%
226	ko00625	Chloroalkane and chloroalkene degradation	58	0.251%
227	ko00513	Various types of N-glycan biosynthesis	57	0.246%
228	ko04725	Cholinergic synapse	56	0.242%
229	ko04726	Serotonergic synapse	54	0.233%
230	ko00073	Cutin, suberine and wax biosynthesis	54	0.233%
231	ko05219	Bladder cancer	54	0.233%
232	ko05146	Amoebiasis	53	0.229%
233	ko04260	Cardiac muscle contraction	52	0.225%
234	ko04320	Dorso-ventral axis formation	52	0.225%
235	ko00902	Monoterpenoid biosynthesis	52	0.225%
236	ko04330	Notch signaling pathway	52	0.225%
237	ko05216	Thyroid cancer	51	0.220%
238	ko04623	Cytosolic DNA-sensing pathway	50	0.216%
239	ko00760	Nicotinate and nicotinamide metabolism	49	0.212%
240	ko04013	MAPK signaling pathway - fly	49	0.212%
241	ko04940	Type I diabetes mellitus	48	0.207%
242	ko00983	Drug metabolism - other enzymes	48	0.207%
243	ko04960	Aldosterone-regulated sodium reabsorption	48	0.207%
244	ko00531	Glycosaminoglycan degradation	47	0.203%
245	ko01220	Degradation of aromatic compounds	47	0.203%
246	ko00904	Diterpenoid biosynthesis	46	0.199%
247	ko05410	Hypertrophic cardiomyopathy (HCM)	46	0.199%
248	ko05416	Viral myocarditis	46	0.199%
249	ko00730	Thiamine metabolism	45	0.194%
250	ko00300	Lysine biosynthesis	43	0.186%
251	ko00908	Zeatin biosynthesis	43	0.186%
252	ko04978	Mineral absorption	43	0.186%
253	ko04750	Inflammatory mediator regulation of TRP channels	42	0.181%
254	ko00790	Folate biosynthesis	41	0.177%
255	ko00590	Arachidonic acid metabolism	41	0.177%
256	ko00903	Limonene and pinene degradation	38	0.164%
257	ko00521	Streptomycin biosynthesis	38	0.164%
258	ko04340	Hedgehog signaling pathway	35	0.151%
259	ko04973	Carbohydrate digestion and absorption	35	0.151%

260	ko00563	Glycosylphosphatidylinositol(GPI)-anchor biosynthesis	34	0.147%
261	ko00740	Riboflavin metabolism	34	0.147%
262	ko00362	Benzoate degradation	33	0.143%
263	ko00660	C5-Branched dibasic acid metabolism	33	0.143%
264	ko05033	Nicotine addiction	32	0.138%
265	ko05032	Morphine addiction	31	0.134%
266	ko05222	Small cell lung cancer	30	0.130%
267	ko04974	Protein digestion and absorption	30	0.130%
268	ko04622	RIG-I-like receptor signaling pathway	30	0.130%
269	ko00626	Naphthalene degradation	30	0.130%
270	ko04122	Sulfur relay system	30	0.130%
271	ko04745	Phototransduction - fly	28	0.121%
272	ko00140	Steroid hormone biosynthesis	28	0.121%
273	ko04970	Salivary secretion	27	0.117%
274	ko00401	Novobiocin biosynthesis	27	0.117%
275	ko04630	Jak-STAT signaling pathway	26	0.112%
276	ko04744	Phototransduction	26	0.112%
277	ko04670	Leukocyte transendothelial migration	25	0.108%
278	ko00905	Brassinosteroid biosynthesis	25	0.108%
279	ko02030	Bacterial chemotaxis	24	0.104%
280	ko00603	Glycosphingolipid biosynthesis - globo series	24	0.104%
281	ko00072	Synthesis and degradation of ketone bodies	23	0.099%
282	ko00430	Taurine and hypotaurine metabolism	23	0.099%
283	ko04740	Olfactory transduction	22	0.095%
284	ko04011	MAPK signaling pathway - yeast	22	0.095%
285	ko04971	Gastric acid secretion	22	0.095%
286	ko00440	Phosphonate and phosphinate metabolism	21	0.091%
287	ko00627	Aminobenzoate degradation	21	0.091%
288	ko05217	Basal cell carcinoma	20	0.086%
289	ko00750	Vitamin B6 metabolism	20	0.086%
290	ko04711	Circadian rhythm - fly	19	0.082%
291	ko00524	Butirosin and neomycin biosynthesis	18	0.078%
292	ko00643	Styrene degradation	17	0.073%
293	ko04964	Proximal tubule bicarbonate reclamation	17	0.073%
294	ko04080	Neuroactive ligand-receptor interaction	17	0.073%
295	ko00624	Polycyclic aromatic hydrocarbon degradation	16	0.069%
296	ko00966	Glucosinolate biosynthesis	16	0.069%
297	ko00361	Chlorocyclohexane and chlorobenzene degradation	16	0.069%
298	ko00253	Tetracycline biosynthesis	14	0.060%
299	ko00514	Other types of O-glycan biosynthesis	13	0.056%
300	ko00550	Peptidoglycan biosynthesis	13	0.056%
301	ko00604	Glycosphingolipid biosynthesis - ganglio series	13	0.056%
302	ko00623	Toluene degradation	12	0.052%
303	ko00540	Lipopolysaccharide biosynthesis	12	0.052%

304	ko04913	Ovarian steroidogenesis	12	0.052%
305	ko00312	beta-Lactam resistance	12	0.052%
306	ko00281	Geraniol degradation	12	0.052%
307	ko00785	Lipoic acid metabolism	12	0.052%
308	ko00471	D-Glutamine and D-glutamate metabolism	11	0.048%
309	ko00363	Bisphenol degradation	11	0.048%
310	ko05143	African trypanosomiasis	11	0.048%
311	ko00523	Polyketide sugar unit biosynthesis	10	0.043%
312	ko01051	Biosynthesis of ansamycins	10	0.043%
313	ko03450	Non-homologous end-joining	10	0.043%
314	ko00534	Glycosaminoglycan biosynthesis - heparan sulfate / heparin	10	0.043%
315	ko00942	Anthocyanin biosynthesis	9	0.039%
316	ko00532	Glycosaminoglycan biosynthesis - chondroitin sulfate / dermatan sulfate	9	0.039%
317	ko04975	Fat digestion and absorption	9	0.039%
318	ko04911	Insulin secretion	8	0.035%
319	ko05030	Cocaine addiction	8	0.035%
320	ko00943	Isoflavonoid biosynthesis	8	0.035%
321	ko00944	Flavone and flavonol biosynthesis	8	0.035%
322	ko04977	Vitamin digestion and absorption	7	0.030%
323	ko02040	Flagellar assembly	7	0.030%
324	ko00351	DDT degradation	7	0.030%
325	ko00364	Fluorobenzoate degradation	7	0.030%
326	ko00232	Caffeine metabolism	7	0.030%
327	ko00930	Caprolactam degradation	6	0.026%
328	ko00622	Xylene degradation	6	0.026%
329	ko00254	Aflatoxin biosynthesis	6	0.026%
330	ko05414	Dilated cardiomyopathy	6	0.026%
331	ko02060	Phosphotransferase system (PTS)	5	0.022%
332	ko04742	Taste transduction	5	0.022%
333	ko05150	Staphylococcus aureus infection	4	0.017%
334	ko05412	Arrhythmogenic right ventricular cardiomyopathy (ARVC)	4	0.017%
335	ko05340	Primary immunodeficiency	3	0.013%
336	ko00901	Indole alkaloid biosynthesis	3	0.013%
337	ko01053	Biosynthesis of siderophore group nonribosomal peptides	3	0.013%
338	ko04060	Cytokine-cytokine receptor interaction	2	0.009%
339	ko04640	Hematopoietic cell lineage	2	0.009%
340	ko01055	Biosynthesis of vancomycin group antibiotics	2	0.009%
341	ko00965	Betalain biosynthesis	2	0.009%
342	ko00473	D-Alanine metabolism	1	0.004%
343	ko04672	Intestinal immune network for IgA production	1	0.004%

344	ko00472	D-Arginine and D-ornithine metabolism	1	0.004%
345	ko05144	Malaria	1	0.004%
346	ko00633	Nitrotoluene degradation	1	0.004%
347	ko04512	ECM-receptor interaction	1	0.004%
348	ko05310	Asthma	1	0.004%
349	ko00642	Ethylbenzene degradation	1	0.004%
350	ko05320	Autoimmune thyroid disease	1	0.004%
351	ko05321	Inflammatory bowel disease (IBD)	1	0.004%
352	ko04514	Cell adhesion molecules (CAMs)	1	0.004%
353	ko00311	Penicillin and cephalosporin biosynthesis	1	0.004%
354	ko04614	Renin-angiotensin system	1	0.004%
355	ko05330	Allograft rejection	1	0.004%
356	ko00621	Dioxin degradation	1	0.004%
