# Supplementary material for: Transcriptome Analysis of Genes Associated with the Artemisinin Biosynthesis by Jasmonic Acid Treatment under the Light in Artemisia annua
Source: Front Plant Sci. 2017 Jun 8;8:971. doi: 10.3389/fpls.2017.00971 (PMC5463050; doi:10.3389/fpls.2017.00971)
Supplement: Supplementary file 3 [file Table3.PDF]

**Table S3** List of genes with more than 1000 FPKM in Light.

| NO. | unigenes | Light | Dark | Light-MJ-4h | Dark-MJ-4h | NR_Description |
|---|---|---|---|---|---|---|
| 1 | c108280_g1 | 38144.96 | 21733.13 | 42627.84 | 13722.35 | ribulose-1,5-bisphosphate carboxylase small subunit [Chrysanthemum x morifolium] |
| 2 | c115931_g3 | 22718.1 | 2068.002 | 16928.1 | 2332.637 | light-harvesting chlorophyll a/b-binding protein (LHCP) precursor [Lactuca sativa] |
| 3 | c110526_g2 | 10133.46 | 1740.587 | 6497.15 | 846.933 | Histone superfamily protein [Theobroma cacao] |
| 4 | c121797_g1 | 9368.375 | 1237.903 | 5305.474 | 764.455 | Histone H3 [Medicago truncatula] |
| 5 | c123054_g1 | 8293.453 | 1254.836 | 5210.556 | 1209.987 | Histone superfamily protein isoform 2, partial [Theobroma cacao] |
| 6 | c123437_g2 | 7946.143 | 571.076 | 4388.36 | 539.519 | unknown [Picea sitchensis] |
| 7 | c99554_g1 | 7329.776 | 101.524 | 4761.711 | 88.657 | light-harvesting chlorophyll a/b-binding protein (LHCP) precursor [Lactuca sativa] |
| 8 | c122677_g1 | 6573.476 | 1184.813 | 3475.362 | 452.671 | PREDICTED: histone H2A.1-like [Malus domestica] |
| 9 | c101392_g1 | 6548.711 | 1099.437 | 775.116 | 1174.585 | hypothetical protein MTR_5g051150 [Medicago truncatula] |
| 10 | c115931_g4 | 5609.594 | 1274.279 | 3314.64 | 870.208 | chlorophyll a/b-binding protein [Tagetes erecta] |
| 11 | c107806_g1 | 5128.086 | 273.645 | 2873.271 | 164.846 | hypothetical protein POPTR_0168s00200g [Populus trichocarpa] |
| 12 | c119518_g1 | 4530.945 | 1420.627 | 3608.76 | 1495.281 | elongation factor 1-alpha [Chrysanthemum seticuspe f. boreale] |
| 13 | c116381_g1 | 4329.402 | 594.913 | 2530.213 | 547.739 | Histone H2A [Medicago H2A3_MEDTR RecName: Full=Probable histone H2A.3 [Medicago truncatula] |
| 14 | c106491_g1 | 3942.784 | 330.702 | 4054.279 | 157.818 | PREDICTED: auxin-binding protein ABP19a-like [Cucumis melo] |
| 15 | c109176_g1 | 3840.372 | 173.883 | 3142.538 | 137.517 | PREDICTED: chlorophyll a-b binding protein 13, chloroplastic-like [Vitis vinifera] |
| 16 | c116381_g2 | 3839.768 | 224.531 | 2225.825 | 221.667 | hypothetical protein SORBIDRAFT_01g039250 [Sorghum bicolor] |
| 17 | c99752_g1 | 3819.344 | 1271.785 | 3695.657 | 1961.603 | PREDICTED: uncharacterized protein LOC102601302 [Solanum tuberosum] |
| 18 | c79423_g1 | 3664.574 | 833.569 | 3868.794 | 715.337 | PREDICTED: photosystem I reaction center subunit II, chloroplastic isoform 1 [Vitis PREDICTED: photosystem I reaction cente |
| 19 | c106303_g1 | 3348.561 | 282.757 | 1582.505 | 204.266 | unnamed protein product [Coffea canephora] |
| 20 | c113946_g1 | 3346.145 | 333.728 | 2223.188 | 216.938 | RecName: Full=Histone H2B.3; AltName: Full=LeH2B-3 histone H2B-3 [Solanum lycopersicum] |

| | | | | | | |
|---|---|---|---|---|---|---|
| 21 | c117256_g1 | 3253.727 | 484.27 | 2774.304 | 463.875 | RecName: Full=Photosystem I reaction center subunit III, chloroplastic; AltName: Full=Light-harvesting complex I 17 kDa protein; AltName: Full=PSI-F; Flags: Precurso |
| 22 | c55334_g1 | 3239.23 | 1191.013 | 3140.814 | 1117.422 | PREDICTED: oxygen-evolving enhancer protein 3-2, chloroplastic-like [Fragaria vesca subsp. vesca] |
| 23 | c112038_g1 | 3177.858 | 130.279 | 1953.265 | 75.154 | hypothetical protein SORBIDRAFT_01g039240 [Sorghum bicolor] |
| 24 | c117757_g2 | 3057.153 | 686.742 | 2233 | 576.999 | hypothetical protein JCGZ_13842 [Jatropha curcas] |
| 25 | c114728_g1 | 2761.255 | 215.969 | 1260.184 | 149.893 | histone H1 [Lens culinaris] |
| 26 | c104773_g1 | 2710.357 | 776.12 | 2814.61 | 418.424 | PREDICTED: chlorophyll a-b binding protein CP29.2, chloroplastic-like [Solanum lycopersicum] |
| 27 | c108802_g2 | 2691.335 | 682.644 | 1610.674 | 663.07 | chlorophyll a/b binding protein [Solanum tuberosum] |
| 28 | c105420_g1 | 2664.849 | 2249.706 | 2584.847 | 1811.978 | RecName: Full=Oxygen-evolving enhancer protein 2, chloroplastic; Short=OEE2; AltName: Full=23 kDa subunit of oxygen evolving system of photosystem II; AltName: Ful |
| 29 | c115211_g1 | 2540.998 | 295.6 | 2404.324 | 204.414 | hypothetical protein PHAVU_004G128200g [Phaseolus vulgaris] |
| 30 | c112337_g2 | 2511.366 | 389.59 | 2824.956 | 304.866 | Photosystem I reaction center subunit V [Morus notabilis] |
| 31 | c115931_g1 | 2477.689 | 63.378 | 1412.706 | 48.6 | light-harvesting chlorophyll a/b-binding protein (LHCP) precursor [Lactuca sativa] |
| 32 | c72844_g1 | 2472.822 | 1905.786 | 3283.791 | 1862.759 | unnamed protein product [Solanum tuberosum] |
| 33 | c113198_g1 | 2464.958 | 229.64 | 1369.208 | 92.056 | hypothetical protein CARUB_v10002094mg, partial [Capsella rubella] |
| 34 | c65617_g1 | 2436.044 | 244.768 | 2890.192 | 259.249 | GAPDH [Pericallis cruenta] |
| 35 | c91717_g1 | 2296.763 | 383.783 | 2588.808 | 299.204 | hypothetical protein PRUPE_ppa005158mg [Prunus persica] |
| 36 | c118638_g4 | 2294.586 | 336.614 | 1849.102 | 275.606 | PREDICTED: chlorophyll a-b binding protein CP24 10A, chloroplastic-like [Cicer arietinum] |
| 37 | c118061_g3 | 2265.945 | 205.253 | 1649.266 | 202.585 | PREDICTED: LOW QUALITY PROTEIN: ubiquitin-60S ribosomal protein L40 [Vitis vinifera] |
| 38 | c104506_g1 | 2248.44 | 695.757 | 1614.278 | 721.442 | ubiquitin extension protein [Capsicum annuum] |
| 39 | c108740_g1 | 2238.069 | 495.962 | 1998.955 | 423.541 | RecName: Full=Chlorophyll a-b binding protein, chloroplastic; AltName: Full=LHCI type |

| | | | | | | |
|---|---|---|---|---|---|---|
| | | | | | | II CAB; Flags: Precursor chlorophyll binding protein pr |
| 40 | c101477_g1 | 2225.68 | 353.886 | 2371.049 | 345.218 | ferredoxin [Helianthus annuus] |
| 41 | c100744_g1 | 2217.714 | 523.008 | 2159.576 | 464.355 | photosystem I psaH protein [Nicotiana sylvestris] |
| 42 | c74610_g1 | 2208.972 | 500.644 | 1757.633 | 527.438 | hypothetical protein POPTR_0018s12630g [Populus trichocarpa] |
| 43 | c104707_g1 | 2177.061 | 494.933 | 2081.835 | 448.737 | PREDICTED: photosystem II reaction center W protein, chloroplastic-like [Cicer arietinum] |
| 44 | c95586_g1 | 2169.778 | 314.546 | 1663.283 | 321.204 | 60s acidic ribosomal protein [Hyacinthus orientalis] |
| 45 | c36363_g1 | 2167.1 | 59.263 | 237.372 | 73.418 | hypothetical protein (mitochondrion) [Capsicum annuum] |
| 46 | c91739_g1 | 2159.612 | 2294.208 | 2264.728 | 2846.913 | unnamed protein product [Coffea canephora] |
| 47 | c109630_g1 | 2107.414 | 620.486 | 2392.898 | 313.289 | PREDICTED: plastocyanin, chloroplastic isoform 1 [Vitis vinifera] |
| 48 | c91772_g1 | 2100.679 | 366.476 | 1927.733 | 211.544 | unnamed protein product [Coffea canephora] |
| 49 | c118890_g1 | 2086.683 | 1254.853 | 1672.784 | 870.957 | hypothetical protein JCGZ_20960 [Jatropha curcas] |
| 50 | c107860_g1 | 2081.908 | 171.267 | 1375.159 | 128.373 | PREDICTED: chlorophyll a-b binding protein 6A, chloroplastic-like [Fragaria vesca subsp. vesca] |
| 51 | c86761_g1 | 2068.596 | 1639.203 | 1811.222 | 1241.584 | putative 16kDa membrane protein [Nicotiana tabacum] |
| 52 | c123358_g2 | 2053.963 | 1442.215 | 1564.205 | 1529.085 | histone H1 [Solanum histone H1 [Solanum lycopersicum] |
| 53 | c106130_g1 | 2037.711 | 794.203 | 1557.975 | 815.78 | unnamed protein product [Vitis vinifera] |
| 54 | c98029_g1 | 2036.617 | 239.894 | 1991.189 | 198.992 | fructose-bisphosphate aldolase 3 [Camellia oleifera] |
| 55 | c117393_g1 | 1988.647 | 1528.925 | 2296.523 | 1778.147 | heat shock protein 70 [Chrysanthemum indicum] |
| 56 | c111775_g1 | 1971.347 | 985.367 | 1484.773 | 764.741 | PREDICTED: 60S ribosomal protein L12 [Vitis vinifera] |
| 57 | c107860_g3 | 1969.557 | 114.393 | 1363.601 | 88.214 | PREDICTED: chlorophyll a-b binding protein 6A, chloroplastic isoform X1 [Cucumis melo] |
| 58 | c115931_g2 | 1895.443 | 36.82 | 1140.102 | 30.258 | hypothetical protein PRUPE_ppa009987mg [Prunus persica] |
| 59 | c102613_g1 | 1836.179 | 434.135 | 1803.857 | 336.767 | unnamed protein product [Coffea canephora] |
| 60 | c86046_g1 | 1810.73 | 577.667 | 1981.633 | 250.391 | ribulose-1,5-bisphosphate carboxylase/oxygenase small subunit [Lactuca sativa] |
| 61 | c106991_g1 | 1805.499 | 2319.624 | 1257.336 | 2062.776 | Histone H3 [Medicago truncatula] |

| | | | | | | |
|---|---|---|---|---|---|---|
| 62 | c102627_g1 | 1788.495 | 891.995 | 1456.204 | 968.943 | 60S ribosomal protein L32-1 [Morus notabilis] |
| 63 | c123131_g1 | 1783.161 | 383.164 | 1026.472 | 457.465 | PREDICTED: histone H1-like [Solanum lycopersicum] |
| 64 | c117322_g1 | 1770.168 | 2297.033 | 2049.851 | 2381.727 | tonoplast intrinsic protein [Jatropha curcas] |
| 65 | c111775_g2 | 1752.856 | 481.907 | 1352.075 | 472.954 | PREDICTED: 60S ribosomal protein L12 [Vitis vinifera] |
| 66 | c122260_g1 | 1742.702 | 328.644 | 1117.273 | 234.533 | PREDICTED: tubulin alpha-4 chain-like [Cucumis sativus] |
| 67 | c117293_g1 | 1725.287 | 531.343 | 1245.488 | 473.979 | PREDICTED: 60S ribosomal protein L5-like [Solanum tuberosum] |
| 68 | c108813_g2 | 1718.62 | 1082.174 | 2113.319 | 1740.943 | Multidrug resistance protein ABC transporter family [Medicago truncatula] |
| 69 | c101494_g3 | 1705.821 | 438.469 | 1119.565 | 382.449 | Os07g0139600 [Oryza sativa Japonica Group] |
| 70 | c102558_g1 | 1694.31 | 419.958 | 1233.061 | 429.406 | 60S ribosomal protein L36 isoform 1 [Theobroma cacao] |
| 71 | c101225_g1 | 1643.56 | 413.698 | 1012.621 | 428.971 | PREDICTED: 40S ribosomal protein SA-like isoform X1 [Solanum tuberosum] |
| 72 | c99805_g1 | 1636.71 | 372.667 | 1681.973 | 361.104 | photosystem I reaction center subunit X psaK [Nicotiana tabacum] |
| 73 | c116622_g3 | 1575.874 | 389.669 | 1103.634 | 359.544 | beta-tubulin [Cichorium intybus] |
| 74 | c112193_g1 | 1552.316 | 898.273 | 1549.62 | 620.288 | hypothetical protein EUGRSUZ_K023621 [Eucalyptus grandis] |
| 75 | c113461_g1 | 1547.256 | 1507.52 | 1410.437 | 2220.002 | aquaporin PIP1 [Chrysanthemum x morifolium] |
| 76 | c2057_g1 | 1529.978 | 423.289 | 1802.378 | 399.628 | Photosystem II core complex proteins psbY [Populus trichocarpa] |
| 77 | c111275_g2 | 1506.216 | 631.734 | 1612.376 | 518.368 | vacuolar-type H+-pyrophosphatase [Solanum lycopersicum] |
| 78 | c110537_g1 | 1505.486 | 534.587 | 1159.425 | 441.773 | PREDICTED: 40S ribosomal protein S3-3-like [Cucumis sativus] |
| 79 | c107096_g2 | 1503.526 | 368.752 | 858.542 | 384.25 | PREDICTED: probable histone H2A.1-like [Solanum lycopersicum] |
| 80 | c94914_g1 | 1485.804 | 583.404 | 1547.328 | 538.272 | hypothetical protein JCGZ_17594 [Jatropha curcas] |
| 81 | c113946_g2 | 1470.099 | 210.433 | 803.518 | 142.061 | histone HTB9 [Arabidopsis thaliana] |
| 82 | c95167_g1 | 1469.655 | 111.333 | 1710.875 | 92.056 | putative photosystem II protein [Gossypioides kirkii] |
| 83 | c73597_g1 | 1466.315 | 230.015 | 823.142 | 127.865 | RecName: Full=Amorpha-4,11-diene synthase AF138959_1 amorpha-4,11-diene synthase [Artemisia annua] |
| 84 | c107156_g1 | 1456.32 | 568.913 | 1330.66 | 730.715 | putative S-adenosylmethionine synthetase [Capsicum annuum] |
| 85 | c82709_g2 | 1451.1 | 150.786 | 2383.453 | 120.143 | chloroplast ribulose 1,5-bisphosphate carboxylase/oxygenase activase [Flaveria bidentis] |
| 86 | c107125_g1 | 1446.838 | 553.681 | 1001.608 | 564.604 | hypothetical protein JCGZ_05652 [Jatropha curcas] |

| | | | | | | |
|---|---|---|---|---|---|---|
| 87 | c94594_g1 | 1441.481 | 48.913 | 1772.897 | 96.425 | germin-like protein 6 precursor [Vitis vinifera] |
| 88 | c117656_g2 | 1440.228 | 269.774 | 981.438 | 248.415 | PREDICTED: 60S ribosomal protein L35-like [Cicer arietinum] |
| 89 | c118123_g1 | 1437.766 | 267.071 | 942.379 | 263.266 | PREDICTED: 60S ribosomal protein L26-1 [Malus domestica] |
| 90 | c61713_g1 | 1435.726 | 164.039 | 2135.235 | 126.202 | chloroplast ribulose 1,5-bisphosphate carboxylase/oxygenase activase [Flaveria bidentis] |
| 91 | c55227_g1 | 1411.154 | 332.359 | 1221.425 | 187.937 | photosystem I subunit XI [Nicotiana attenuata] |
| 92 | c105801_g2 | 1398.025 | 472.438 | 1295.216 | 442.558 | PREDICTED: pollen-specific protein C13 [Prunus mume] |
| 93 | c118061_g2 | 1397.717 | 1636.893 | 1751.826 | 2507.818 | hypothetical protein CARUB_v10001095mg, partial [Capsella rubella] |
| 94 | c104703_g1 | 1392.999 | 516.321 | 991.818 | 507.829 | 60S ribosomal protein L18a-1 [Populus trichocarpa] |
| 95 | c95612_g1 | 1382.092 | 2409.351 | 1291.111 | 2059.968 | RecName: Full=Oxygen-evolving enhancer protein 1, chloroplastic; Short=OEE1; AltName: Full=33 kDa subunit of oxygen evolving system of photosystem II; AltName: Ful |
| 96 | c118638_g3 | 1375.858 | 285.73 | 1346.034 | 274.516 | hypothetical protein MIMGU_mgv1a011622mg [Erythranthe guttata] |
| 97 | c34369_g1 | 1368.974 | 660.995 | 1359.573 | 809.342 | hypothetical protein POPTR_0001s05690g [Populus trichocarpa] |
| 98 | c123878_g2 | 1362.9 | 268.205 | 907.413 | 309.29 | hypothetical protein MIMGU_mgv1a012493mg [Erythranthe guttata] |
| 99 | c116408_g1 | 1340.026 | 322.907 | 940.888 | 280.621 | 60S ribosomal protein L3 [Morus notabilis] |
| 100 | c104772_g1 | 1330.35 | 34.431 | 2124.733 | 956.317 | extracellular jacalin-like lectin [Helianthus annuus] |
| 101 | c106308_g1 | 1323.079 | 173.551 | 742.776 | 155.278 | unnamed protein product [Coffea canephora] |
| 102 | c115166_g2 | 1309.14 | 359.981 | 843.857 | 377.914 | 40S ribosomal protein S4 [Populus trichocarpa] |
| 103 | c72019_g2 | 1309.129 | 227.687 | 1106.004 | 384.389 | hypothetical protein MIMGU_mgv1a015002mg [Erythranthe guttata] |
| 104 | c71638_g1 | 1282.586 | 119.284 | 791.659 | 79.976 | non-specific lipid-transfer protein [Helianthus annuus] |
| 105 | c123126_g1 | 1270.938 | 232.779 | 1253.442 | 298.391 | S-adenosyl-L-homocysteine hydrolase [Chrysanthemum x morifolium] |
| 106 | c108997_g2 | 1249.341 | 575.819 | 1201.533 | 578.209 | aquaporin PIP2;2 [Vitis plasma membrane 2;2 aquaporin [Vitis vinifera] |
| 107 | c102551_g1 | 1244.098 | 276.339 | 1099.696 | 225.361 | Photosystem I subunit E-2 [Theobroma cacao] |
| 108 | c99284_g1 | 1243.517 | 480.477 | 911.607 | 411.46 | Ribosomal protein L37 [Medicago unknown [Medicago truncatula] |
| 109 | c110129_g2 | 1241.203 | 266.897 | 800.97 | 254.446 | beta tubulin 3 [Hordeum vulgare subsp. vulgare] |
| 110 | c82695_g1 | 1232.758 | 153.149 | 984.142 | 188.952 | PREDICTED: elongation factor 1-alpha-like [Brachypodium distachyon] |

| | | | | | | |
|---|---|---|---|---|---|---|
| 111 | c113501_g1 | 1221.179 | 397.036 | 1492.093 | 330.736 | hypothetical protein JCGZ_14304 [Jatropha curcas] |
| 112 | c112855_g1 | 1220.689 | 1013.267 | 1408.145 | 1389.158 | cyclophilin 2 [Tagetes patula] |
| 113 | c105863_g1 | 1217.202 | 336.919 | 888.401 | 288.749 | hypothetical protein PHAVU_001G200900g [Phaseolus vulgaris] |
| 114 | c52032_g1 | 1200.687 | 4.926 | 521.402 | 0 | putative chloroplast chlorophyll a/b-binding protein [Carya cathayensis] |
| 115 | c99901_g1 | 1187.809 | 222.5 | 786.652 | 242.226 | PREDICTED: 60S ribosomal protein L27a-3-like [Citrus sinensis] |
| 116 | c124396_g5 | 1187.296 | 288.651 | 1336.256 | 226.627 | chloroplast phosphoglycerate kinase 3 [Helianthus annuus] |
| 117 | c123008_g4 | 1174.93 | 237.252 | 691.957 | 223.874 | beta tubulin [Oryza sativa Japonica Group] |
| 118 | c114509_g1 | 1174.52 | 208.671 | 1174.055 | 159.729 | Plastid-specific ribosomal protein 4 [Theobroma cacao] |
| 119 | c9840_g1 | 1173.312 | 550.211 | 1220.29 | 563.662 | hybrid proline-rich protein [Gossypium hirsutum] |
| 120 | c103326_g2 | 1172.15 | 294.117 | 791.325 | 272.586 | 60S ribosomal protein L37a-2 [Medicago truncatula] |
| 121 | c110826_g1 | 1168.651 | 104.793 | 1144.852 | 111.969 | unknown [Lotus japonicus] |
| 122 | c116379_g1 | 1167.306 | 660.062 | 854.436 | 530.495 | RecName: Full=29 kDa ribonucleoprotein A, chloroplastic; AltName: Full=CP29A; Flags: 29kD A ribonucleoprotein [Nicotiana sylvestr |
| 123 | c34559_g1 | 1146.7 | 171.651 | 833.155 | 159.018 | putative 40S ribosomal protein S8-like protein [Picrorhiza kurrooa] |
| 124 | c124396_g4 | 1143.965 | 274.395 | 1328.468 | 224.281 | chloroplast phosphoglycerate kinase 3 [Helianthus annuus] |
| 125 | c100512_g3 | 1138.392 | 416.593 | 828.226 | 414.305 | PREDICTED: 40S ribosomal protein S25-4-like [Fragaria vesca subsp. vesca] |
| 126 | c84686_g1 | 1138.221 | 396.565 | 833.833 | 423.448 | 60S ribosomal protein [Phaseolus vulgaris] |
| 127 | c118425_g1 | 1125.422 | 140.838 | 1218.488 | 122.24 | peroxiredoxin [Phaseolus vulgaris] |
| 128 | c92293_g1 | 1123.074 | 352.099 | 1092.754 | 491.888 | cyclophilin [Gerbera hybrid cultivar] |
| 129 | c100200_g1 | 1119.553 | 285.634 | 997.825 | 264.088 | glyceraldehyde-3-phosphate dehydrogenase [Eleutherococcus senticosus] |
| 130 | c106775_g1 | 1116.59 | 431.589 | 892.662 | 430.366 | 60S ribosomal protein L19-2 [Morus notabilis] |
| 131 | c106881_g1 | 1112.304 | 406.06 | 881.537 | 471.31 | putative 40S ribosomal protein S9 [Artemisia annua] |
| 132 | c67042_g1 | 1112.213 | 158.642 | 733.631 | 166.943 | hypothetical protein MIMGU_mgv1a015450mg [Erythranthe guttata] |
| 133 | c91717_g2 | 1096.246 | 124.969 | 1658.833 | 95.585 | chloroplast ribulose 1,5-bisphosphate carboxylase/oxygenase activase [Flaveria bidentis] |
| 134 | c100090_g1 | 1081.795 | 363.573 | 930.531 | 415.764 | 40S ribosomal protein S15 [Morus notabilis] |
| 135 | c104493_g1 | 1077.168 | 306.943 | 819.994 | 308.948 | hypothetical protein MIMGU_mgv1a012042mg [Erythranthe guttata] |

| | | | | | | |
|---|---|---|---|---|---|---|
| 136 | c94194_g1 | 1074.626 | 119.842 | 773.191 | 115.498 | hypothetical protein PHAVU_002G233500g, partial [Phaseolus vulgaris] |
| 137 | c110461_g1 | 1069.703 | 171.093 | 772.657 | 201.892 | PREDICTED: 40S ribosomal protein S16-like isoform 1 [Solanum lycopersicum] |
| 138 | c115931_g5 | 1069.338 | 467.189 | 744.968 | 240.139 | chlorophyll a/b binding protein of LHCII type I [Lilium longiflorum] |
| 139 | c95537_g1 | 1067.788 | 248.099 | 711.092 | 254.418 | PREDICTED: 60S ribosomal protein L7a isoform X2 [Prunus mume] |
| 140 | c74192_g1 | 1062.329 | 183.273 | 793.105 | 214.638 | 60S ribosomal protein L23 [Morus notabilis] |
| 141 | c99409_g1 | 1060.722 | 316.647 | 778.787 | 317.953 | PREDICTED: 40S ribosomal protein S23-like [Zea mays] |
| 142 | c116015_g1 | 1058.34 | 254.917 | 823.476 | 267.986 | PREDICTED: 60S ribosomal protein L4-like [Citrus sinensis] |
| 143 | c157660_g1 | 1050.123 | 239.816 | 746.336 | 279.263 | PREDICTED: 40S ribosomal protein S14-2-like [Solanum tuberosum] |
| 144 | c105449_g1 | 1048.163 | 189.376 | 751.142 | 217.705 | hypothetical protein PRUPE_ppa013661mg [Prunus persica] |
| 145 | c98023_g1 | 1042.031 | 205.323 | 735.166 | 221.334 | unknown [Lotus japonicus] |
| 146 | c125418_g1 | 1041.609 | 242.51 | 1110.031 | 232.565 | PREDICTED: elongation factor Tu, chloroplastic-like [Vitis vinifera] |
| 147 | c104104_g1 | 1031.968 | 367.182 | 773.914 | 384.5 | unnamed protein product [Vitis vinifera] |
| 148 | c101641_g2 | 1031.455 | 154.309 | 993.286 | 191.206 | PREDICTED: acyl carrier protein 4, chloroplastic [Vitis vinifera] |
| 149 | c96828_g1 | 1028.389 | 355.168 | 670.308 | 307.452 | PREDICTED: 60S ribosomal protein L13-2-like [Solanum lycopersicum] |
| 150 | c109857_g1 | 1027.283 | 342.961 | 724.82 | 366.009 | hypothetical protein PRUPE_ppa011659mg [Prunus persica] |
| 151 | c72819_g1 | 1009.219 | 341.261 | 808.012 | 301.144 | PREDICTED: 60S ribosomal protein L27 [Vitis vinifera] |
| 152 | c104369_g1 | 1008.615 | 252.449 | 684.181 | 261.724 | Ubiquitin [Medicago truncatula] |
| 153 | c104521_g1 | 1007.396 | 172.985 | 719.213 | 172.235 | unnamed protein product [Vitis vinifera] |
| 154 | c110621_g1 | 1003.897 | 310.056 | 780.167 | 364.937 | unnamed protein product [Vitis vinifera] |
