# Supplementary material for: Transcriptome Analysis of Genes Associated with the Artemisinin Biosynthesis by Jasmonic Acid Treatment under the Light in Artemisia annua
Source: Front Plant Sci. 2017 Jun 8;8:971. doi: 10.3389/fpls.2017.00971 (PMC5463050; doi:10.3389/fpls.2017.00971)
Supplement: Supplementary file 5 [file Table5.PDF]

Table S5 List of genes with more than 1000 FPKM in Light-MJ-4h.

NO.	unigenes	Light	Dark	Light-MJ-4h	Dark-MJ-4h	NR_Description
1	c108280_g1	38144.96	21733.13	42627.84	13722.35	ribulose-1,5-bisphosphate carboxylase small subunit [Chrysanthemum x morifolium]
2	c115931_g3	22718.1	2068.002	16928.1	2332.637	light-harvesting chlorophyll a/b-binding protein (LHCP) precursor [Lactuca sativa]
3	c110526_g2	10133.46	1740.587	6497.15	846.933	Histone superfamily protein [Theobroma cacao]
4	c121797_g1	9368.375	1237.903	5305.474	764.455	Histone H3 [Medicago truncatula]
5	c123054_g1	8293.453	1254.836	5210.556	1209.987	Histone superfamily protein isoform 2, partial [Theobroma cacao]
6	c121106_g1	970.914	646.573	4899.46	2101.18	aurone synthase [Coreopsis grandiflora]
7	c99554_g1	7329.776	101.524	4761.711	88.657	light-harvesting chlorophyll a/b-binding protein (LHCP) precursor [Lactuca sativa]
8	c123437_g2	7946.143	571.076	4388.36	539.519	unknown [Picea sitchensis]
9	c110279_g1	830.915	22.591	4268.6	506.112	aurone synthase [Coreopsis grandiflora]
10	c106491_g1	3942.784	330.702	4054.279	157.818	PREDICTED: auxin-binding protein ABP19a-like [Cucumis melo]
11	c79423_g1	3664.574	833.569	3868.794	715.337	PREDICTED: photosystem I reaction center subunit II, chloroplastic isoform 1 [Vitis PREDICTED: photosystem I reaction cente
12	c99752_g1	3819.344	1271.785	3695.657	1961.603	PREDICTED: uncharacterized protein LOC102601302 [Solanum tuberosum]
13	c119518_g1	4530.945	1420.627	3608.76	1495.281	elongation factor 1-alpha [Chrysanthemum seticuspe f. boreale]
14	c122677_g1	6573.476	1184.813	3475.362	452.671	PREDICTED: histone H2A.1-like [Malus domestica]
15	c115931_g4	5609.594	1274.279	3314.64	870.208	chlorophyll a/b-binding protein [Tagetes erecta]
16	c72844_g1	2472.822	1905.786	3283.791	1862.759	unnamed protein product [Solanum tuberosum]
17	c109176_g1	3840.372	173.883	3142.538	137.517	PREDICTED: chlorophyll a-b binding protein 13, chloroplastic-like [Vitis vinifera]
18	c55334_g1	3239.23	1191.013	3140.814	1117.422	PREDICTED: oxygen-evolving enhancer protein 3-2, chloroplastic-like [Fragaria vesca subsp. vesca]
19	c65617_g1	2436.044	244.768	2890.192	259.249	GAPDH [Pericallis cruenta]
20	c107806_g1	5128.086	273.645	2873.271	164.846	hypothetical protein POPTR_0168s00200g [Populus trichocarpa]
21	c112337_g2	2511.366	389.59	2824.956	304.866	Photosystem I reaction center subunit V [Morus notabilis]

22	c104773_g1	2710.357	776.12	2814.61	418.424	PREDICTED: chlorophyll a-b binding protein CP29.2, chloroplastic-like [Solanum lycopersicum]
23	c117256_g1	3253.727	484.27	2774.304	463.875	RecName: Full=Photosystem I reaction center subunit III, chloroplastic; AltName: Full=Light-harvesting complex I 17 kDa protein; AltName: Full=PSI-F; Flags: Precurso
24	c91717_g1	2296.763	383.783	2588.808	299.204	hypothetical protein PRUPE_ppa005158mg [Prunus persica]
25	c105420_g1	2664.849	2249.706	2584.847	1811.978	RecName: Full=Oxygen-evolving enhancer protein 2, chloroplastic; Short=OEE2; AltName: Full=23 kDa subunit of oxygen evolving system of photosystem II; AltName: Ful
26	c116381_g1	4329.402	594.913	2530.213	547.739	Histone H2A [Medicago H2A3_MEDTR RecName: Full=Probable histone H2A.3 [Medicago truncatula]
27	c115211_g1	2540.998	295.6	2404.324	204.414	hypothetical protein PHAVU_004G128200g [Phaseolus vulgaris]
28	c109630_g1	2107.414	620.486	2392.898	313.289	PREDICTED: plastocyanin, chloroplastic isoform 1 [Vitis vinifera]
29	c82709_g2	1451.1	150.786	2383.453	120.143	chloroplast ribulose 1,5-bisphosphate carboxylase/oxygenase activase [Flaveria bidentis]
30	c101477_g1	2225.68	353.886	2371.049	345.218	ferredoxin [Helianthus annuus]
31	c117393_g1	1988.647	1528.925	2296.523	1778.147	heat shock protein 70 [Chrysanthemum indicum]
32	c91739_g1	2159.612	2294.208	2264.728	2846.913	unnamed protein product [Coffea canephora]
33	c117757_g2	3057.153	686.742	2233	576.999	hypothetical protein JCGZ_13842 [Jatropha curcas]
34	c116381_g2	3839.768	224.531	2225.825	221.667	hypothetical protein SORBIDRAFT_01g039250 [Sorghum bicolor]
35	c113946_g1	3346.145	333.728	2223.188	216.938	RecName: Full=Histone H2B.3; AltName: Full=LeH2B-3 histone H2B-3 [Solanum lycopersicum]
36	c123158_g2	691.109	2321.917	2176.842	3804.061	phenylalanine ammonia lyase [Chrysanthemum x morifolium]
37	c100744_g1	2217.714	523.008	2159.576	464.355	photosystem I psaH protein [Nicotiana sylvestris]
38	c61713_g1	1435.726	164.039	2135.235	126.202	chloroplast ribulose 1,5-bisphosphate carboxylase/oxygenase activase [Flaveria bidentis]
39	c104772_g1	1330.35	34.431	2124.733	956.317	extracellular jacalin-like lectin [Helianthus annuus]
40	c108813_g2	1718.62	1082.174	2113.319	1740.943	Multidrug resistance protein ABC transporter family [Medicago truncatula]
41	c104707_g1	2177.061	494.933	2081.835	448.737	PREDICTED: photosystem II reaction center W protein, chloroplastic-like [Cicer

						arietinum]
42	c117322_g1	1770.168	2297.033	2049.851	2381.727	tonoplast intrinsic protein [Jatropha curcas]
43	c108740_g1	2238.069	495.962	1998.955	423.541	RecName: Full=Chlorophyll a-b binding protein, chloroplastic; AltName: Full=LHCI type II CAB; Flags: Precursor chlorophyll binding protein pr
44	c98029_g1	2036.617	239.894	1991.189	198.992	fructose-bisphosphate aldolase 3 [Camellia oleifera]
45	c86046_g1	1810.73	577.667	1981.633	250.391	ribulose-1,5-bisphosphate carboxylase/oxygenase small subunit [Lactuca sativa]
46	c112038_g1	3177.858	130.279	1953.265	75.154	hypothetical protein SORBIDRAFT_01g039240 [Sorghum bicolor]
47	c91772_g1	2100.679	366.476	1927.733	211.544	unnamed protein product [Coffea canephora]
48	c118638_g4	2294.586	336.614	1849.102	275.606	PREDICTED: chlorophyll a-b binding protein CP24 10A, chloroplastic-like [Cicer arietinum]
49	c86761_g1	2068.596	1639.203	1811.222	1241.584	putative 16kDa membrane protein [Nicotiana tabacum]
50	c102613_g1	1836.179	434.135	1803.857	336.767	unnamed protein product [Coffea canephora]
51	c2057_g1	1529.978	423.289	1802.378	399.628	Photosystem II core complex proteins psbY [Populus trichocarpa]
52	c94594_g1	1441.481	48.913	1772.897	96.425	germin-like protein 6 precursor [Vitis vinifera]
53	c74610_g1	2208.972	500.644	1757.633	527.438	hypothetical protein POPTR_0018s12630g [Populus trichocarpa]
54	c118061_g2	1397.717	1636.893	1751.826	2507.818	hypothetical protein CARUB_v10001095mg, partial [Capsella rubella]
55	c95167_g1	1469.655	111.333	1710.875	92.056	putative photosystem II protein [Gossypioides kirkii]
56	c99805_g1	1636.71	372.667	1681.973	361.104	photosystem I reaction center subunit X psaK [Nicotiana tabacum]
57	c118890_g1	2086.683	1254.853	1672.784	870.957	hypothetical protein JCGZ_20960 [Jatropha curcas]
58	c95586_g1	2169.778	314.546	1663.283	321.204	60s acidic ribosomal protein [Hyacinthus orientalis]
59	c91717_g2	1096.246	124.969	1658.833	95.585	chloroplast ribulose 1,5-bisphosphate carboxylase/oxygenase activase [Flaveria bidentis]
60	c118061_g3	2265.945	205.253	1649.266	202.585	PREDICTED: LOW QUALITY PROTEIN: ubiquitin-60S ribosomal protein L40 [Vitis vinifera]
61	c104506_g1	2248.44	695.757	1614.278	721.442	ubiquitin extension protein [Capsicum annuum]
62	c111275_g2	1506.216	631.734	1612.376	518.368	vacuolar-type H+-pyrophosphatase [Solanum lycopersicum]
63	c108802_g2	2691.335	682.644	1610.674	663.07	chlorophyll a/b binding protein [Solanum tuberosum]

64	c106303_g1	3348.561	282.757	1582.505	204.266	unnamed protein product [Coffea canephora]
65	c111296_g2	333.576	3.671	1573.717	43.622	cytochrome P450 mono-oxygenase [Artemisia annua]
66	c123358_g2	2053.963	1442.215	1564.205	1529.085	histone H1 [Solanum histone H1 [Solanum lycopersicum]
67	c106130_g1	2037.711	794.203	1557.975	815.78	unnamed protein product [Vitis vinifera]
68	c112193_g1	1552.316	898.273	1549.62	620.288	hypothetical protein EUGRSUZ_K023621 [Eucalyptus grandis]
69	c94914_g1	1485.804	583.404	1547.328	538.272	hypothetical protein JCGZ_17594 [Jatropha curcas]
70	c113501_g1	1221.179	397.036	1492.093	330.736	hypothetical protein JCGZ_14304 [Jatropha curcas]
71	c111775_g1	1971.347	985.367	1484.773	764.741	PREDICTED: 60S ribosomal protein L12 [Vitis vinifera]
72	c102627_g1	1788.495	891.995	1456.204	968.943	60S ribosomal protein L32-1 [Morus notabilis]
73	c115931_g1	2477.689	63.378	1412.706	48.6	light-harvesting chlorophyll a/b-binding protein (LHCP) precursor [Lactuca sativa]
74	c113461_g1	1547.256	1507.52	1410.437	2220.002	aquaporin PIP1 [Chrysanthemum x morifolium]
75	c112855_g1	1220.689	1013.267	1408.145	1389.158	cyclophilin 2 [Tagetes patula]
76	c107860_g1	2081.908	171.267	1375.159	128.373	PREDICTED: chlorophyll a-b binding protein 6A, chloroplastic-like [Fragaria vesca subsp. vesca]
77	c113198_g1	2464.958	229.64	1369.208	92.056	hypothetical protein CARUB_v10002094mg, partial [Capsella rubella]
78	c107860_g3	1969.557	114.393	1363.601	88.214	PREDICTED: chlorophyll a-b binding protein 6A, chloroplastic isoform X1 [Cucumis melo]
79	c34369_g1	1368.974	660.995	1359.573	809.342	hypothetical protein POPTR_0001s05690g [Populus trichocarpa]
80	c111775_g2	1752.856	481.907	1352.075	472.954	PREDICTED: 60S ribosomal protein L12 [Vitis vinifera]
81	c118638_g3	1375.858	285.73	1346.034	274.516	hypothetical protein MIMGU_mgv1a011622mg [Erythranthe guttata]
82	c124396_g5	1187.296	288.651	1336.256	226.627	chloroplast phosphoglycerate kinase 3 [Helianthus annuus]
83	c107156_g1	1456.32	568.913	1330.66	730.715	putative S-adenosylmethionine synthetase [Capsicum annuum]
84	c124396_g4	1143.965	274.395	1328.468	224.281	chloroplast phosphoglycerate kinase 3 [Helianthus annuus]
85	c103418_g7	416.899	6481.672	1312.092	7722.244	PREDICTED: glycine-rich protein-like [Citrus sinensis]
86	c105801_g2	1398.025	472.438	1295.216	442.558	PREDICTED: pollen-specific protein C13 [Prunus mume]
87	c95612_g1	1382.092	2409.351	1291.111	2059.968	RecName: Full=Oxygen-evolving enhancer protein 1, chloroplastic; Short=OEE1;

						AltName: Full=33 kDa subunit of oxygen evolving system of photosystem II; AltName: Ful
88	c100020_g1	879.671	46.786	1263.766	38.358	PREDICTED: glyceraldehyde-3-phosphate dehydrogenase B, chloroplastic-like [Solanum tuberosum]
89	c114728_g1	2761.255	215.969	1260.184	149.893	histone H1 [Lens culinaris]
90	c106991_g1	1805.499	2319.624	1257.336	2062.776	Histone H3 [Medicago truncatula]
91	c123126_g1	1270.938	232.779	1253.442	298.391	S-adenosyl-L-homocysteine hydrolase [Chrysanthemum x morifolium]
92	c117293_g1	1725.287	531.343	1245.488	473.979	PREDICTED: 60S ribosomal protein L5-like [Solanum tuberosum]
93	c111272_g2	179.194	50.727	1241.305	614.941	unnamed protein product [Coffea canephora]
94	c102558_g1	1694.31	419.958	1233.061	429.406	60S ribosomal protein L36 isoform 1 [Theobroma cacao]
95	c115234_g1	888.036	2111.693	1229.624	2186.688	hypothetical protein EUGRSUZ_D01322 [Eucalyptus grandis]
96	c55227_g1	1411.154	332.359	1221.425	187.937	photosystem I subunit XI [Nicotiana attenuata]
97	c9840_g1	1173.312	550.211	1220.29	563.662	hybrid proline-rich protein [Gossypium hirsutum]
98	c118425_g1	1125.422	140.838	1218.488	122.24	peroxiredoxin [Phaseolus vulgaris]
99	c107990_g2	909.633	412.242	1209.565	376.658	PREDICTED: cytochrome b6-f complex iron-sulfur subunit, chloroplastic isoform 1 [Vitis hypothetical protein VITISV_020089 [Viti
100	c108997_g2	1249.341	575.819	1201.533	578.209	aquaporin PIP2;2 [Vitis plasma membrane 2;2 aquaporin [Vitis vinifera]
101	c124101_g1	976.157	140.707	1200.41	100.11	RecName: Full=Glutamine synthetase, chloroplastic; AltName: Full=GS2; AltName: Full=Glutamate--ammonia ligase; Flags: Precursor glutamine
102	c115997_g2	283.806	2.816	1191.143	46.227	chalcone synthase [Saussurea medusa]
103	c114509_g1	1174.52	208.671	1174.055	159.729	Plastid-specific ribosomal protein 4 [Theobroma cacao]
104	c110537_g1	1505.486	534.587	1159.425	441.773	PREDICTED: 40S ribosomal protein S3-3-like [Cucumis sativus]
105	c110826_g1	1168.651	104.793	1144.852	111.969	unknown [Lotus japonicus]
106	c115931_g2	1895.443	36.82	1140.102	30.258	hypothetical protein PRUPE_ppa009987mg [Prunus persica]
107	c84514_g1	301.038	141.936	1128.198	133.065	RecName: Full=Raucaffricine-O-beta-D-glucosidase; Short=Raucaffricine beta-glucosidase; Short=RsRG; AltName: Full=Vomilenine glucosyltransferase;

						Short=RsVGT [Rauvol
108	c125375_g1	318.886	116.207	1122.736	328.307	unnamed protein product [Vitis vinifera]
109	c101494_g3	1705.821	438.469	1119.565	382.449	Os07g0139600 [Oryza sativa Japonica Group]
110	c122260_g1	1742.702	328.644	1117.273	234.533	PREDICTED: tubulin alpha-4 chain-like [Cucumis sativus]
111	c125418_g1	1041.609	242.51	1110.031	232.565	PREDICTED: elongation factor Tu, chloroplastic-like [Vitis vinifera]
112	c72019_g2	1309.129	227.687	1106.004	384.389	hypothetical protein MIMGU_mgv1a015002mg [Erythranthe guttata]
113	c116622_g3	1575.874	389.669	1103.634	359.544	beta-tubulin [Cichorium intybus]
114	c102551_g1	1244.098	276.339	1099.696	225.361	Photosystem I subunit E-2 [Theobroma cacao]
115	c108491_g1	572.512	16.854	1097.96	8.137	RecName: Full=Carbonic anhydrase 1; AltName: Full=Carbonate dehydratase 1 carbonic anhydrase 1 [Flaveria linearis]
116	c92293_g1	1123.074	352.099	1092.754	491.888	cyclophilin [Gerbera hybrid cultivar]
117	c113461_g3	977.923	136.095	1068.101	137.249	aquaporin PIP2 [Chrysanthemum x morifolium]
118	c124396_g3	788.336	205.681	1067.979	163.387	chloroplast phosphoglycerate kinase 3 [Helianthus annuus]
119	c90965_g1	766.021	248.84	1056.008	244.582	PREDICTED: calvin cycle protein CP12-like [Vitis vinifera]
120	c107488_g1	770.237	77.826	1031.667	38.662	putative chloroplast thiazole biosynthetic protein [Nicotiana tabacum]
121	c123131_g1	1783.161	383.164	1026.472	457.465	PREDICTED: histone H1-like [Solanum lycopersicum]
122	c72815_g1	861.002	2807.677	1020.209	2887.958	metallothionein 1 [Aster tripolium]
123	c105992_g2	670.674	400.036	1013.567	647.249	hypothetical protein [Plantago major]
124	c101225_g1	1643.56	413.698	1012.621	428.971	PREDICTED: 40S ribosomal protein SA-like isoform X1 [Solanum tuberosum]
125	c107125_g1	1446.838	553.681	1001.608	564.604	hypothetical protein JCGZ_05652 [Jatropha curcas]
