# Supplementary material for: Transcriptome Analysis of Genes Associated with the Artemisinin Biosynthesis by Jasmonic Acid Treatment under the Light in Artemisia annua
Source: Front Plant Sci. 2017 Jun 8;8:971. doi: 10.3389/fpls.2017.00971 (PMC5463050; doi:10.3389/fpls.2017.00971)
Supplement: Supplementary file 6 [file Table6.PDF]

Table S6 List of genes with more than 1000 FPKM in Dark-MJ-4h.

NO.	unigenes	Light	Dark	Light-MJ-4h	Dark-MJ-4h	NR_Description
1	c108280_g1	38144.96	21733.13	42627.84	13722.35	ribulose-1,5-bisphosphate carboxylase small subunit [Chrysanthemum x morifolium]
2	c103418_g7	416.899	6481.672	1312.092	7722.244	PREDICTED: glycine-rich protein-like [Citrus sinensis]
3	c105797_g1	192.779	5773.229	198.947	4413.303	hypothetical protein MIMGU_mgv1a016425mg [Erythranthe guttata]
4	c123158_g2	691.109	2321.917	2176.842	3804.061	phenylalanine ammonia lyase [Chrysanthemum x morifolium]
5	c122156_g1	33.621	1650.067	55.146	3628.778	tonoplast intrinsic protein [Gossypium hirsutum]
6	c100721_g1	61.065	3192.393	37.28	3174.509	PREDICTED: stem-specific protein TSJT1-like [Prunus mume]
7	c72815_g1	861.002	2807.677	1020.209	2887.958	metallothionein 1 [Aster tripolium]
8	c91739_g1	2159.612	2294.208	2264.728	2846.913	unnamed protein product [Coffea canephora]
9	c116293_g1	6.77	3109.781	12.026	2798.377	asparagine synthetase [Helianthus annuus]
10	c118061_g2	1397.717	1636.893	1751.826	2507.818	hypothetical protein CARUB_v10001095mg, partial [Capsella rubella]
11	c57266_g1	179.159	2767.352	187.187	2422.144	hypothetical protein CICLE_v10002843mg [Citrus clementina]
12	c117322_g1	1770.168	2297.033	2049.851	2381.727	tonoplast intrinsic protein [Jatropha curcas]
13	c118989_g3	512.884	1963.933	625.909	2347.507	DnaJ family protein [Populus unknown [Populus trichocarpa]
14	c115931_g3	22718.1	2068.002	16928.1	2332.637	light-harvesting chlorophyll a/b-binding protein (LHCP) precursor [Lactuca sativa]
15	c113461_g1	1547.256	1507.52	1410.437	2220.002	aquaporin PIP1 [Chrysanthemum x morifolium]
16	c115234_g1	888.036	2111.693	1229.624	2186.688	hypothetical protein EUGRSUZ_D01322 [Eucalyptus grandis]
17	c121106_g1	970.914	646.573	4899.46	2101.18	aurone synthase [Coreopsis grandiflora]
18	c118260_g1	441.106	4763.344	449.034	2079.937	UPA22 [Capsicum annuum]
19	c106991_g1	1805.499	2319.624	1257.336	2062.776	Histone H3 [Medicago truncatula]
20	c95612_g1	1382.092	2409.351	1291.111	2059.968	RecName: Full=Oxygen-evolving enhancer protein 1, chloroplastic; Short=OEE1; AltName: Full=33 kDa subunit of oxygen evolving system of photosystem II; AltName: Ful
21	c99752_g1	3819.344	1271.785	3695.657	1961.603	PREDICTED: uncharacterized protein LOC102601302 [Solanum tuberosum]

22	c72844_g1	2472.822	1905.786	3283.791	1862.759	unnamed protein product [Solanum tuberosum]
23	c15772_g1	311.945	1833.619	507.573	1855.628	serine-pyruvate aminotransferase, putative [Ricinus communis]
24	c105420_g1	2664.849	2249.706	2584.847	1811.978	RecName: Full=Oxygen-evolving enhancer protein 2, chloroplastic; Short=OEE2; AltName: Full=23 kDa subunit of oxygen evolving system of photosystem II; AltName: Ful
25	c117393_g1	1988.647	1528.925	2296.523	1778.147	heat shock protein 70 [Chrysanthemum indicum]
26	c108813_g2	1718.62	1082.174	2113.319	1740.943	Multidrug resistance protein ABC transporter family [Medicago truncatula]
27	c110401_g1	42.852	832.732	69.175	1666.897	wound-induced protein 1-like protein [Phaseolus vulgaris]
28	c109135_g2	9.687	914.508	13.183	1630.886	PREDICTED: inositol oxygenase 1-like [Malus domestica]
29	c116365_g2	307.876	138.501	848.307	1596.241	pathogenesis-related protein [Zinnia elegans]
30	c101562_g1	7.351	514.333	57.594	1592.057	unnamed protein product [Coffea canephora]
31	c108862_g1	176.401	1623.927	258.798	1549.276	hypothetical protein PRUPE_ppa013385mg [Prunus persica]
32	c123358_g2	2053.963	1442.215	1564.205	1529.085	histone H1 [Solanum histone H1 [Solanum lycopersicum]
33	c119518_g1	4530.945	1420.627	3608.76	1495.281	elongation factor 1-alpha [Chrysanthemum seticuspe f. boreale]
34	c99131_g1	417.047	1261.488	336.639	1490.783	PREDICTED: ubiquitin-conjugating enzyme E2 10-like [Solanum lycopersicum]
35	c119486_g3	22.6	544.369	18.545	1421.3	PREDICTED: arogenate dehydrogenase 1, chloroplastic [Vitis vinifera]
36	c112855_g1	1220.689	1013.267	1408.145	1389.158	cyclophilin 2 [Tagetes patula]
37	c78183_g1	481.975	791.317	888.913	1388.059	RecName: Full=Defensin SD2; AltName: Full=Flower-specific gamma-thionin; Flags: Precursor AF141131_1 defensin [Helianthus annuus]
38	c98279_g1	848.637	912.991	704.529	1372.838	hypothetical protein L484_011178 [Morus notabilis]
39	c118528_g2	9.938	920.567	62.967	1352.555	hypothetical protein CARUB_v10015079mg [Capsella rubella]
40	c120681_g1	11.762	800.245	18.879	1351.844	Methionine gamma-lyase [Theobroma cacao]
41	c118040_g4	36.869	790.82	39.226	1280.181	PREDICTED: aldehyde dehydrogenase family 7 member B4-like [Solanum lycopersicum]
42	c107458_g1	373.671	1247.416	362.872	1254.172	catalase [Homogyne alpina]
43	c123792_g1	20.435	1018.229	11.281	1252.454	PREDICTED: zinc finger CCCH domain-containing protein 20-like [Solanum tuberosum]
44	c86761_g1	2068.596	1639.203	1811.222	1241.584	putative 16kDa membrane protein [Nicotiana tabacum]

45	c123054_g1	8293.453	1254.836	5210.556	1209.987	Histone superfamily protein isoform 2, partial [Theobroma cacao]
46	c115255_g1	125.64	798.327	352.681	1178.418	D-mannose binding lectin protein with Apple-like carbohydrate-binding domain, putative [Theobroma cacao]
47	c101392_g1	6548.711	1099.437	775.116	1174.585	hypothetical protein MTR_5g051150 [Medicago truncatula]
48	c118199_g1	9.004	1002.055	7.42	1172.451	hypothetical protein POPTR_0011s03990g [Populus trichocarpa]
49	c105444_g1	17.506	421.31	18.2	1126.779	Periplasmic beta-glucosidase, putative [Theobroma cacao]
50	c55334_g1	3239.23	1191.013	3140.814	1117.422	PREDICTED: oxygen-evolving enhancer protein 3-2, chloroplastic-like [Fragaria vesca subsp. vesca]
51	c118040_g2	32.458	710.048	34.943	1079.988	hypothetical protein EUTSA_v10011405mg [Eutrema salsugineum]
52	c102705_g1	63.72	328.156	269.211	1070.374	hypothetical protein MIMGU_mgv1a008881mg [Erythranthe guttata]
53	c118034_g1	0.798	1202.434	0.656	1038.888	7S globulin [Sesamum indicum]
54	c118528_g1	7.978	757.767	47.548	1035.895	PREDICTED: isocitrate lyase-like [Fragaria vesca subsp. vesca]
55	c124652_g1	72.382	412.321	114.598	1025.458	PDH2 [Helianthus tuberosus]
56	c125933_g1	5.493	118.526	34.198	1006.931	Zinc finger protein [Chrysanthemum x morifolium]
57	c119202_g1	538.515	1587.229	523.449	1006.192	xyloglucan endotransglucosylase/hydrolase 4 [Actinidia deliciosa]
