# Supplementary material for: Transcriptome Analysis of Genes Associated with the Artemisinin Biosynthesis by Jasmonic Acid Treatment under the Light in Artemisia annua
Source: Front Plant Sci. 2017 Jun 8;8:971. doi: 10.3389/fpls.2017.00971 (PMC5463050; doi:10.3389/fpls.2017.00971)
Supplement: Supplementary file 7 [file Table7.PDF]

Table S7 DEGs between Light and Dark annotated with KEGG metabolic pathways.

NO.	Pathway	Pathway ID	Sample number	Background number	P-Value	Corrected P-Value	Percentage (%)
1	Ribosome	ko03010	187	752	7.10408E-13	2.18E-10	24.8670%
2	Biosynthesis of amino acids	ko01230	134	801	0.01227023	0.156956686	16.7291%
3	Carbon metabolism	ko01200	126	893	0.324870177	0.999991646	14.1097%
4	Plant hormone signal transduction	ko04075	92	597	0.12551541	0.789679355	15.4104%
5	Starch and sucrose metabolism	ko00500	90	594	0.161218515	0.913111407	15.1515%
6	Phenylpropanoid biosynthesis	ko00940	80	417	0.00297863	0.04572197	19.1847%
7	Purine metabolism	ko00230	78	390	0.001284474	0.028166686	20.0000%
8	Fatty acid metabolism	ko01212	74	363	0.001082877	0.025572551	20.3857%
9	Cell cycle	ko04110	71	305	5.20378E-05	0.003993898	23.2787%
10	Glycolysis / Gluconeogenesis	ko00010	71	442	0.095661073	0.667453393	16.0633%
11	Protein processing in endoplasmic reticulum	ko04141	65	769	0.999957102	0.999991646	8.4525%
12	Endocytosis	ko04144	60	523	0.896047701	0.999991646	11.4723%
13	Pyrimidine metabolism	ko00240	59	280	0.001706883	0.034448636	21.0714%
14	DNA replication	ko03030	58	256	0.000425905	0.014528083	22.6563%
15	Amino sugar and nucleotide sugar metabolism	ko00520	57	315	0.026564924	0.281221785	18.0952%
16	RNA transport	ko03013	56	507	0.933952287	0.999991646	11.0454%
17	Cell cycle - yeast	ko04111	54	237	0.00058596	0.016643107	22.7848%
18	RNA degradation	ko03018	51	341	0.262959601	0.999991646	14.9560%
19	Spliceosome	ko03040	50	622	0.999942097	0.999991646	8.0386%
20	Plant-pathogen interaction	ko04626	49	468	0.963958425	0.999991646	10.4701%
21	Oxidative phosphorylation	ko00190	48	427	0.899366586	0.999991646	11.2412%
22	Neurotrophin signaling pathway	ko04722	47	368	0.657148589	0.999991646	12.7717%

23	Cysteine and methionine metabolism	ko00270	45	285	0.180356035	0.945296866	15.7895%
24	Pyruvate metabolism	ko00620	43	340	0.67506405	0.999991646	12.6471%
25	Aminoacyl-tRNA biosynthesis	ko00970	42	316	0.556803757	0.999991646	13.2911%
26	Terpenoid backbone biosynthesis	ko00900	41	169	0.000916503	0.023447214	24.2604%
27	Fatty acid biosynthesis	ko00061	41	178	0.002097443	0.037877359	23.0337%
28	Glycine, serine and threonine metabolism	ko00260	41	263	0.213598522	0.957128513	15.5894%
29	Carbon fixation in photosynthetic organisms	ko00710	41	268	0.246259764	0.999991646	15.2985%
30	Meiosis - yeast	ko04113	40	179	0.003702817	0.054131665	22.3464%
31	alpha-Linolenic acid metabolism	ko00592	39	165	0.001795369	0.034448636	23.6364%
32	Arginine and proline metabolism	ko00330	38	219	0.090937592	0.649252111	17.3516%
33	Toll-like receptor signaling pathway	ko04620	38	273	0.450408237	0.999991646	13.9194%
34	Ubiquitin mediated proteolysis	ko04120	38	393	0.982632597	0.999991646	9.6692%
35	Ribosome biogenesis in eukaryotes	ko03008	37	239	0.238922331	0.991204804	15.4812%
36	Phenylalanine metabolism	ko00360	37	248	0.305777286	0.999991646	14.9194%
37	AMPK signaling pathway	ko04152	36	299	0.761903111	0.999991646	12.0401%
38	Antigen processing and presentation	ko04612	36	325	0.88701669	0.999991646	11.0769%
39	Photosynthesis	ko00195	35	125	0.000242244	0.010624124	28.0000%
40	Glutathione metabolism	ko00480	35	234	0.307226802	0.999991646	14.9573%
41	Methane metabolism	ko00680	35	241	0.364914234	0.999991646	14.5228%
42	Photosynthesis - antenna proteins	ko00196	34	89	1.61115E-06	0.000164874	38.2022%
43	Fanconi anemia pathway	ko03460	33	122	0.000596333	0.016643107	27.0492%
44	Biosynthesis of unsaturated fatty acids	ko01040	33	167	0.031684483	0.313778592	19.7605%
45	Glyoxylate and dicarboxylate metabolism	ko00630	33	229	0.385721806	0.999991646	14.4105%
46	Estrogen signaling pathway	ko04915	33	313	0.927066529	0.999991646	10.5431%
47	MAPK signaling pathway	ko04010	32	275	0.807699489	0.999991646	11.6364%
48	Mismatch repair	ko03430	31	97	7.65033E-05	0.004697301	31.9588%

49	Progesterone-mediated oocyte maturation	ko04914	31	135	0.007027309	0.093799297	22.9630%
50	2-Oxocarboxylic acid metabolism	ko01210	30	180	0.163586734	0.913111407	16.6667%
51	Glycerolipid metabolism	ko00561	30	215	0.457125288	0.999991646	13.9535%
52	Galactose metabolism	ko00052	30	218	0.485170761	0.999991646	13.7615%
53	NF-kappa B signaling pathway	ko04064	30	227	0.567793127	0.999991646	13.2159%
54	Apoptosis	ko04210	30	230	0.594419429	0.999991646	13.0435%
55	Insulin signaling pathway	ko04910	30	268	0.855047378	0.999991646	11.1940%
56	Oocyte meiosis	ko04114	30	297	0.949113967	0.999991646	10.1010%
57	PI3K-Akt signaling pathway	ko04151	30	341	0.992862964	0.999991646	8.7977%
58	HIF-1 signaling pathway	ko04066	29	200	0.384515538	0.999991646	14.5000%
59	Glycerophospholipid metabolism	ko00564	29	248	0.790476913	0.999991646	11.6935%
60	Phagosome	ko04145	29	257	0.841218235	0.999991646	11.2840%
61	Biotin metabolism	ko00780	28	87	0.000150495	0.007700343	32.1839%
62	Drug metabolism - cytochrome P450	ko00982	28	137	0.032771879	0.314405211	20.4380%
63	Nucleotide excision repair	ko03420	28	147	0.061982886	0.500756475	19.0476%
64	Central carbon metabolism in cancer	ko05230	28	194	0.396086226	0.999991646	14.4330%
65	Pentose and glucuronate interconversions	ko00040	28	215	0.596223806	0.999991646	13.0233%
66	Phenylalanine, tyrosine and tryptophan biosynthesis	ko00400	27	136	0.046135435	0.416575837	19.8529%
67	Flavonoid biosynthesis	ko00941	26	138	0.076319552	0.557859584	18.8406%
68	Circadian rhythm - plant	ko04712	26	145	0.111301944	0.742819499	17.9310%
69	Porphyrin and chlorophyll metabolism	ko00860	26	160	0.215120089	0.957128513	16.2500%
70	FoxO signaling pathway	ko04068	26	215	0.727204663	0.999991646	12.0930%
71	Homologous recombination	ko03440	25	118	0.030472275	0.311832944	21.1864%
72	Fatty acid elongation	ko00062	24	87	0.002503598	0.042700257	27.5862%
73	Gap junction	ko04540	23	114	0.054493331	0.470080895	20.1754%
74	Metabolism of xenobiotics by cytochrome P450	ko00980	23	128	0.126040027	0.789679355	17.9688%

75	Peroxisome	ko04146	23	235	0.946248925	0.999991646	9.7872%
76	Sphingolipid signaling pathway	ko04071	23	248	0.970334398	0.999991646	9.2742%
77	Alanine, aspartate and glutamate metabolism	ko00250	22	142	0.303400604	0.999991646	15.4930%
78	Pentose phosphate pathway	ko00030	22	165	0.551552044	0.999991646	13.3333%
79	Fructose and mannose metabolism	ko00051	22	182	0.715841391	0.999991646	12.0879%
80	Fatty acid degradation	ko00071	22	184	0.73260042	0.999991646	11.9565%
81	Fc gamma R-mediated phagocytosis	ko04666	22	210	0.893278437	0.999991646	10.4762%
82	cAMP signaling pathway	ko04024	22	211	0.897449453	0.999991646	10.4265%
83	mRNA surveillance pathway	ko03015	22	314	0.999594177	0.999991646	7.0064%
84	Tyrosine metabolism	ko00350	21	131	0.26211758	0.999991646	16.0305%
85	Valine, leucine and isoleucine degradation	ko00280	21	157	0.546505947	0.999991646	13.3758%
86	Proteasome	ko03050	21	189	0.827501027	0.999991646	11.1111%
87	Oxytocin signaling pathway	ko04921	21	192	0.845496073	0.999991646	10.9375%
88	Linoleic acid metabolism	ko00591	20	73	0.005823067	0.081258254	27.3973%
89	Sesquiterpenoid and triterpenoid biosynthesis	ko00909	20	99	0.068558655	0.539679668	20.2020%
90	Cyanoamino acid metabolism	ko00460	20	169	0.740461558	0.999991646	11.8343%
91	Choline metabolism in cancer	ko05231	20	218	0.965715577	0.999991646	9.1743%
92	p53 signaling pathway	ko04115	19	81	0.025384369	0.278321469	23.4568%
93	Base excision repair	ko03410	19	87	0.043329982	0.403100132	21.8391%
94	Stilbenoid, diarylheptanoid and gingerol biosynthesis	ko00945	19	90	0.055123493	0.470080895	21.1111%
95	cGMP-PKG signaling pathway	ko04022	19	163	0.757407938	0.999991646	11.6564%
96	Regulation of actin cytoskeleton	ko04810	19	205	0.958289693	0.999991646	9.2683%
97	PPAR signaling pathway	ko03320	18	170	0.863228021	0.999991646	10.5882%
98	Citrate cycle (TCA cycle)	ko00020	18	197	0.960368484	0.999991646	9.1371%
99	Lysosome	ko04142	18	203	0.970884584	0.999991646	8.8670%
100	Ras signaling pathway	ko04014	17	167	0.891923473	0.999991646	10.1796%

101	Valine, leucine and isoleucine biosynthesis	ko00290	16	76	0.07499219	0.557859584	21.0526%
102	Ubiquinone and other terpenoid-quinone biosynthesis	ko00130	16	99	0.287814828	0.999991646	16.1616%
103	RNA polymerase	ko03020	16	106	0.375288048	0.999991646	15.0943%
104	Tryptophan metabolism	ko00380	16	123	0.591262043	0.999991646	13.0081%
105	Ascorbate and aldarate metabolism	ko00053	16	127	0.63792496	0.999991646	12.5984%
106	Focal adhesion	ko04510	15	125	0.7021358	0.999991646	12.0000%
107	beta-Alanine metabolism	ko00410	15	127	0.722677937	0.999991646	11.8110%
108	Retinol metabolism	ko00830	14	95	0.419553454	0.999991646	14.7368%
109	Nitrogen metabolism	ko00910	14	101	0.502601511	0.999991646	13.8614%
110	GnRH signaling pathway	ko04912	14	131	0.829576402	0.999991646	10.6870%
111	Protein export	ko03060	14	132	0.836847263	0.999991646	10.6061%
112	Carbon fixation pathways in prokaryotes	ko00720	14	155	0.946975148	0.999991646	9.0323%
113	Wnt signaling pathway	ko04310	14	181	0.988313602	0.999991646	7.7348%
114	One carbon pool by folate	ko00670	13	69	0.170525051	0.934842688	18.8406%
115	Carotenoid biosynthesis	ko00906	13	72	0.204783708	0.952554519	18.0556%
116	Steroid biosynthesis	ko00100	13	74	0.229186333	0.969752348	17.5676%
117	VEGF signaling pathway	ko04370	13	88	0.422483088	0.999991646	14.7727%
118	Lysine degradation	ko00310	13	96	0.53679167	0.999991646	13.5417%
119	Calcium signaling pathway	ko04020	13	115	0.764526902	0.999991646	11.3043%
120	Axon guidance	ko04360	13	117	0.783256514	0.999991646	11.1111%
121	Glutamatergic synapse	ko04724	13	150	0.958013458	0.999991646	8.6667%
122	Other glycan degradation	ko00511	12	59	0.131253665	0.805897502	20.3390%
123	Histidine metabolism	ko00340	12	67	0.223764373	0.969752348	17.9104%
124	Butanoate metabolism	ko00650	12	83	0.455704625	0.999991646	14.4578%
125	Chemokine signaling pathway	ko04062	12	91	0.572903546	0.999991646	13.1868%
126	B cell receptor signaling pathway	ko04662	12	104	0.735841711	0.999991646	11.5385%

127	Rap1 signaling pathway	ko04015	12	107	0.766904985	0.999991646	11.2150%
128	Synaptic vesicle cycle	ko04721	12	186	0.997920541	0.999991646	6.4516%
129	Carbohydrate digestion and absorption	ko04973	11	35	0.016760659	0.204701113	31.4286%
130	Tropane, piperidine and pyridine alkaloid biosynthesis	ko00960	11	64	0.273862033	0.999991646	17.1875%
131	Vasopressin-regulated water reabsorption	ko04962	11	64	0.273862033	0.999991646	17.1875%
132	Fc epsilon RI signaling pathway	ko04664	11	73	0.412879301	0.999991646	15.0685%
133	Selenocompound metabolism	ko00450	11	75	0.444524489	0.999991646	14.6667%
134	Natural killer cell mediated cytotoxicity	ko04650	11	86	0.610899157	0.999991646	12.7907%
135	Osteoclast differentiation	ko04380	11	87	0.624851844	0.999991646	12.6437%
136	Sulfur metabolism	ko00920	11	88	0.638545499	0.999991646	12.5000%
137	Adherens junction	ko04520	11	109	0.85650407	0.999991646	10.0917%
138	mTOR signaling pathway	ko04150	11	132	0.960049762	0.999991646	8.3333%
139	Long-term potentiation	ko04720	11	134	0.964647315	0.999991646	8.2090%
140	Adrenergic signaling in cardiomyocytes	ko04261	11	153	0.989734449	0.999991646	7.1895%
141	Inositol phosphate metabolism	ko00562	11	156	0.991651049	0.999991646	7.0513%
142	Thyroid hormone signaling pathway	ko04919	11	183	0.998845589	0.999991646	6.0109%
143	Drug metabolism - other enzymes	ko00983	10	48	0.144430774	0.865221307	20.8333%
144	Monoterpenoid biosynthesis	ko00902	10	52	0.195660855	0.945296866	19.2308%
145	Pancreatic secretion	ko04972	10	62	0.348966006	0.999991646	16.1290%
146	Thyroid hormone synthesis	ko04918	10	72	0.514316142	0.999991646	13.8889%
147	GABAergic synapse	ko04727	10	85	0.703843068	0.999991646	11.7647%
148	Ether lipid metabolism	ko00565	10	89	0.751605879	0.999991646	11.2360%
149	Vascular smooth muscle contraction	ko04270	10	90	0.762676589	0.999991646	11.1111%
150	Streptomycin biosynthesis	ko00521	9	38	0.098733191	0.673579769	23.6842%
151	Zeatin biosynthesis	ko00908	9	43	0.15796714	0.913111407	20.9302%
152	Arachidonic acid metabolism	ko00590	9	45	0.185588619	0.945296866	20.0000%

153	Diterpenoid biosynthesis	ko00904	9	46	0.200144288	0.945296866	19.5652%
154	Cardiac muscle contraction	ko04260	9	52	0.295869576	0.999991646	17.3077%
155	Chloroalkane and chloroalkene degradation	ko00625	9	58	0.400241054	0.999991646	15.5172%
156	Pantothenate and CoA biosynthesis	ko00770	9	70	0.604079263	0.999991646	12.8571%
157	Retrograde endocannabinoid signaling	ko04723	9	74	0.664130903	0.999991646	12.1622%
158	Signaling pathways regulating pluripotency of stem cells	ko04550	9	75	0.678275671	0.999991646	12.0000%
159	Endocrine and other factor-regulated calcium reabsorption	ko04961	9	80	0.743373321	0.999991646	11.2500%
160	T cell receptor signaling pathway	ko04660	9	92	0.860739436	0.999991646	9.7826%
161	NOD-like receptor signaling pathway	ko04621	9	103	0.926226721	0.999991646	8.7379%
162	Melanogenesis	ko04916	9	107	0.942357424	0.999991646	8.4112%
163	Hippo signaling pathway	ko04390	9	118	0.971855929	0.999991646	7.6271%
164	Phosphatidylinositol signaling system	ko04070	9	155	0.99819246	0.999991646	5.8065%
165	ABC transporters	ko02010	9	217	0.999991646	0.999991646	4.1475%
166	Isoquinoline alkaloid biosynthesis	ko00950	8	63	0.617044409	0.999991646	12.6984%
167	Long-term depression	ko04730	8	75	0.783259961	0.999991646	10.6667%
168	Regulation of autophagy	ko04140	8	88	0.895755451	0.999991646	9.0909%
169	Adipocytokine signaling pathway	ko04920	8	99	0.948081456	0.999991646	8.0808%
170	Dopaminergic synapse	ko04728	8	135	0.996349531	0.999991646	5.9259%
171	TGF-beta signaling pathway	ko04350	8	147	0.998633103	0.999991646	5.4422%
172	C5-Branched dibasic acid metabolism	ko00660	7	33	0.1904918	0.945296866	21.2121%
173	Lysine biosynthesis	ko00300	7	43	0.382340935	0.999991646	16.2791%
174	Degradation of aromatic compounds	ko01220	7	47	0.463277386	0.999991646	14.8936%
175	Cell cycle - Caulobacter	ko04112	7	58	0.665056025	0.999991646	12.0690%
176	TNF signaling pathway	ko04668	7	59	0.680928865	0.999991646	11.8644%
177	Prolactin signaling pathway	ko04917	7	70	0.822896107	0.999991646	10.0000%
178	ErbB signaling pathway	ko04012	7	82	0.915615714	0.999991646	8.5366%

179	Hippo signaling pathway - fly	ko04391	7	88	0.943623481	0.999991646	7.9545%
180	Propanoate metabolism	ko00640	7	99	0.974346858	0.999991646	7.0707%
181	Two-component system	ko02020	7	116	0.99314018	0.999991646	6.0345%
182	Bile secretion	ko04976	7	124	0.996442105	0.999991646	5.6452%
183	Butirosin and neomycin biosynthesis	ko00524	6	18	0.057070439	0.473530401	33.3333%
184	Steroid hormone biosynthesis	ko00140	6	28	0.210303304	0.957128513	21.4286%
185	Naphthalene degradation	ko00626	6	30	0.250753038	0.999991646	20.0000%
186	Riboflavin metabolism	ko00740	6	34	0.336776518	0.999991646	17.6471%
187	Limonene and pinene degradation	ko00903	6	38	0.425387109	0.999991646	15.7895%
188	Thiamine metabolism	ko00730	6	45	0.573657455	0.999991646	13.3333%
189	Glycosaminoglycan degradation	ko00531	6	47	0.612479659	0.999991646	12.7660%
190	MAPK signaling pathway - fly	ko04013	6	49	0.649243483	0.999991646	12.2449%
191	Dorso-ventral axis formation	ko04320	6	52	0.700210294	0.999991646	11.5385%
192	Serotonergic synapse	ko04726	6	54	0.731284585	0.999991646	11.1111%
193	Cutin, suberine and wax biosynthesis	ko00073	6	54	0.731284585	0.999991646	11.1111%
194	Cholinergic synapse	ko04725	6	56	0.760007585	0.999991646	10.7143%
195	Platelet activation	ko04611	6	67	0.878411249	0.999991646	8.9552%
196	Circadian entrainment	ko04713	6	75	0.92971337	0.999991646	8.0000%
197	Aminobenzoate degradation	ko00627	5	21	0.188845162	0.945296866	23.8095%
198	Leukocyte transendothelial migration	ko04670	5	25	0.280351389	0.999991646	20.0000%
199	Aldosterone-regulated sodium reabsorption	ko04960	5	48	0.769568972	0.999991646	10.4167%
200	Cytosolic DNA-sensing pathway	ko04623	5	50	0.797140596	0.999991646	10.0000%
201	Basal transcription factors	ko03022	5	81	0.980458593	0.999991646	6.1728%
202	Vitamin B6 metabolism	ko00750	4	20	0.316404074	0.999991646	20.0000%
203	Protein digestion and absorption	ko04974	4	30	0.586290866	0.999991646	13.3333%
204	Inflammatory mediator regulation of TRP channels	ko04750	4	42	0.811556092	0.999991646	9.5238%

205	Mineral absorption	ko04978	4	43	0.824721945	0.999991646	9.3023%
206	SNARE interactions in vesicular transport	ko04130	4	78	0.990906654	0.999991646	5.1282%
207	N-Glycan biosynthesis	ko00510	4	93	0.997833771	0.999991646	4.3011%
208	Sphingolipid metabolism	ko00600	4	93	0.997833771	0.999991646	4.3011%
209	Circadian rhythm	ko04710	4	96	0.998386976	0.999991646	4.1667%
210	Non-homologous end-joining	ko03450	3	10	0.194009103	0.945296866	30.0000%
211	Bisphenol degradation	ko00363	3	11	0.226505292	0.969752348	27.2727%
212	Ovarian steroidogenesis	ko04913	3	12	0.259918324	0.999991646	25.0000%
213	Glycosphingolipid biosynthesis - ganglio series	ko00604	3	13	0.293896442	0.999991646	23.0769%
214	Glucosinolate biosynthesis	ko00966	3	16	0.39619588	0.999991646	18.7500%
215	Polycyclic aromatic hydrocarbon degradation	ko00624	3	16	0.39619588	0.999991646	18.7500%
216	Synthesis and degradation of ketone bodies	ko00072	3	23	0.610871471	0.999991646	13.0435%
217	Glycosphingolipid biosynthesis - globo series	ko00603	3	24	0.637072033	0.999991646	12.5000%
218	Brassinosteroid biosynthesis	ko00905	3	25	0.662009739	0.999991646	12.0000%
219	Novobiocin biosynthesis	ko00401	3	27	0.708081299	0.999991646	11.1111%
220	Salivary secretion	ko04970	3	27	0.708081299	0.999991646	11.1111%
221	RIG-I-like receptor signaling pathway	ko04622	3	30	0.767898078	0.999991646	10.0000%
222	Bacterial secretion system	ko03070	3	68	0.992960378	0.999991646	4.4118%
223	Tight junction	ko04530	3	74	0.996182905	0.999991646	4.0541%
224	Anthocyanin biosynthesis	ko00942	2	9	0.381865498	0.999991646	22.2222%
225	D-Glutamine and D-glutamate metabolism	ko00471	2	11	0.467797993	0.999991646	18.1818%
226	Lipopolysaccharide biosynthesis	ko00540	2	12	0.508013443	0.999991646	16.6667%
227	Tetracycline biosynthesis	ko00253	2	14	0.582222706	0.999991646	14.2857%
228	Styrene degradation	ko00643	2	17	0.677431778	0.999991646	11.7647%
229	Proximal tubule bicarbonate reclamation	ko04964	2	17	0.677431778	0.999991646	11.7647%
230	Olfactory transduction	ko04740	2	22	0.796094317	0.999991646	9.0909%

231	Gastric acid secretion	ko04971	2	22	0.796094317	0.999991646	9.0909%
232	MAPK signaling pathway - yeast	ko04011	2	22	0.796094317	0.999991646	9.0909%
233	Phototransduction	ko04744	2	26	0.861362001	0.999991646	7.6923%
234	Phototransduction - fly	ko04745	2	28	0.886253815	0.999991646	7.1429%
235	Taurine and hypotaurine metabolism	ko00430	2	30	0.906943881	0.999991646	6.6667%
236	Benzoate degradation	ko00362	2	33	0.931469467	0.999991646	6.0606%
237	Glycosylphosphatidylinositol(GPI)-anchor biosynthesis	ko00563	2	34	0.938184667	0.999991646	5.8824%
238	Hedgehog signaling pathway	ko04340	2	35	0.944271566	0.999991646	5.7143%
239	Various types of N-glycan biosynthesis	ko00513	2	57	0.99484123	0.999991646	3.5088%
240	Vitamin digestion and absorption	ko04977	1	7	0.636117445	0.999991646	14.2857%
241	Insulin secretion	ko04911	1	8	0.679316686	0.999991646	12.5000%
242	Flavone and flavonol biosynthesis	ko00944	1	8	0.679316686	0.999991646	12.5000%
243	Fat digestion and absorption	ko04975	1	9	0.717388146	0.999991646	11.1111%
244	Polyketide sugar unit biosynthesis	ko00523	1	10	0.750940411	0.999991646	10.0000%
245	Geraniol degradation	ko00281	1	12	0.806569167	0.999991646	8.3333%
246	Lipoic acid metabolism	ko00785	1	12	0.806569167	0.999991646	8.3333%
247	Circadian rhythm - fly	ko04711	1	19	0.920151084	0.999991646	5.2632%
248	Sulfur relay system	ko04122	1	30	0.980124116	0.999991646	3.3333%
249	Folate biosynthesis	ko00790	1	41	0.995054063	0.999991646	2.4390%
250	Notch signaling pathway	ko04330	1	52	0.998769631	0.999991646	1.9231%
251	Collecting duct acid secretion	ko04966	1	63	0.999694024	0.999991646	1.5873%
