# Supplementary material for: Transcriptome Analysis of Genes Associated with the Artemisinin Biosynthesis by Jasmonic Acid Treatment under the Light in Artemisia annua
Source: Front Plant Sci. 2017 Jun 8;8:971. doi: 10.3389/fpls.2017.00971 (PMC5463050; doi:10.3389/fpls.2017.00971)
Supplement: Supplementary file 8 [file Table8.PDF]

Table S8 DEGs between Light and Light-MeJA-4h annotated with KEGG metabolic pathways.

NO.	Pathway	Pathway ID	Sample number	Background number	P-Value	Corrected P-Value	Percentage (%)
1	Oxidative phosphorylation	ko00190	20	427	0.0001833	0.0098965	4.6838%
2	Carbon metabolism	ko01200	19	893	0.2735863	0.988627	2.1277%
3	Phenylpropanoid biosynthesis	ko00940	18	417	0.0009297	0.0334695	4.3165%
4	RNA transport	ko03013	15	507	0.048914	0.3766746	2.9586%
5	Phenylalanine metabolism	ko00360	14	248	0.000288	0.012442	5.6452%
6	Aminoacyl-tRNA biosynthesis	ko00970	14	316	0.0026255	0.0810162	4.4304%
7	Neurotrophin signaling pathway	ko04722	13	368	0.0201004	0.2170841	3.5326%
8	Toll-like receptor signaling pathway	ko04620	12	273	0.0054381	0.1067851	4.3956%
9	NF-kappa B signaling pathway	ko04064	11	227	0.0038411	0.091102	4.8458%
10	Apoptosis	ko04210	11	230	0.0042177	0.091102	4.7826%
11	Starch and sucrose metabolism	ko00500	11	594	0.5128896	0.988627	1.8519%
12	Protein processing in endoplasmic reticulum	ko04141	11	769	0.8205225	0.988627	1.4304%
13	Biosynthesis of amino acids	ko01230	11	801	0.8565032	0.988627	1.3733%
14	Ribosome	ko03010	9	752	0.9258667	0.988627	1.1968%
15	Glutathione metabolism	ko00480	8	234	0.0691133	0.4815638	3.4188%
16	Ribosome biogenesis in eukaryotes	ko03008	8	239	0.0757638	0.4959088	3.3473%
17	DNA replication	ko03030	8	256	0.1011492	0.5904924	3.1250%
18	Endocytosis	ko04144	8	523	0.7294687	0.988627	1.5296%
19	Diterpenoid biosynthesis	ko00904	7	46	4.16E-05	0.0060458	15.2174%
20	Cardiac muscle contraction	ko04260	7	52	8.40E-05	0.0060458	13.4615%
21	AMPK signaling pathway	ko04152	7	299	0.3040214	0.988627	2.3411%
22	Fatty acid metabolism	ko01212	7	363	0.4875122	0.988627	1.9284%

23	Purine metabolism	ko00230	7	390	0.5615971	0.988627	1.7949%
24	Plant hormone signal transduction	ko04075	7	597	0.9136089	0.988627	1.1725%
25	Spliceosome	ko03040	7	622	0.9318755	0.988627	1.1254%
26	Metabolism of xenobiotics by cytochrome P450	ko00980	6	128	0.0331039	0.2860176	4.6875%
27	Drug metabolism - cytochrome P450	ko00982	6	137	0.0432235	0.345788	4.3796%
28	alpha-Linolenic acid metabolism	ko00592	6	165	0.0860099	0.5464161	3.6364%
29	Biosynthesis of unsaturated fatty acids	ko01040	6	167	0.0897245	0.5537281	3.5928%
30	Cyanoamino acid metabolism	ko00460	6	169	0.093525	0.5611498	3.5503%
31	Synaptic vesicle cycle	ko04721	6	186	0.1291895	0.7071156	3.2258%
32	Glycine, serine and threonine metabolism	ko00260	6	263	0.3459601	0.988627	2.2814%
33	Carbon fixation in photosynthetic organisms	ko00710	6	268	0.3617027	0.988627	2.2388%
34	MAPK signaling pathway	ko04010	6	275	0.3838017	0.988627	2.1818%
35	Estrogen signaling pathway	ko04915	6	313	0.5021684	0.988627	1.9169%
36	Antigen processing and presentation	ko04612	6	325	0.5380119	0.988627	1.8462%
37	Pyruvate metabolism	ko00620	6	340	0.5811751	0.988627	1.7647%
38	Glycolysis / Gluconeogenesis	ko00010	6	442	0.8097335	0.988627	1.3575%
39	Pentose phosphate pathway	ko00030	5	165	0.1870102	0.8793777	3.0303%
40	PPAR signaling pathway	ko03320	5	170	0.2023936	0.9298339	2.9412%
41	Central carbon metabolism in cancer	ko05230	5	194	0.2811481	0.988627	2.5773%
42	Galactose metabolism	ko00052	5	218	0.3646238	0.988627	2.2936%
43	Plant-pathogen interaction	ko04626	5	468	0.9245938	0.988627	1.0684%
44	Monoterpenoid biosynthesis	ko00902	4	52	0.0175982	0.2111782	7.6923%
45	Retrograde endocannabinoid signaling	ko04723	4	74	0.0505721	0.3766746	5.4054%
46	RNA polymerase	ko03020	4	106	0.133284	0.7071156	3.7736%
47	Fanconi anemia pathway	ko03460	4	122	0.1872749	0.8793777	3.2787%
48	Carbon fixation pathways in prokaryotes	ko00720	4	155	0.3139796	0.988627	2.5806%

49	Meiosis - yeast	ko04113	4	179	0.4104009	0.988627	2.2346%
50	Oxytocin signaling pathway	ko04921	4	192	0.4616448	0.988627	2.0833%
51	HIF-1 signaling pathway	ko04066	4	200	0.4924036	0.988627	2.0000%
52	Arginine and proline metabolism	ko00330	4	219	0.5622108	0.988627	1.8265%
53	Glyoxylate and dicarboxylate metabolism	ko00630	4	229	0.596773	0.988627	1.7467%
54	Methane metabolism	ko00680	4	241	0.63602	0.988627	1.6598%
55	Insulin signaling pathway	ko04910	4	268	0.7148504	0.988627	1.4925%
56	Pyrimidine metabolism	ko00240	4	280	0.7455944	0.988627	1.4286%
57	Amino sugar and nucleotide sugar metabolism	ko00520	4	315	0.8207758	0.988627	1.2698%
58	Linoleic acid metabolism	ko00591	3	73	0.1535016	0.7710776	4.1096%
59	GABAergic synapse	ko04727	3	85	0.2066298	0.9298339	3.5294%
60	Sesquiterpenoid and triterpenoid biosynthesis	ko00909	3	99	0.272746	0.988627	3.0303%
61	mTOR signaling pathway	ko04150	3	132	0.4317762	0.988627	2.2727%
62	Phenylalanine, tyrosine and tryptophan biosynthesis	ko00400	3	136	0.4504302	0.988627	2.2059%
63	Terpenoid backbone biosynthesis	ko00900	3	169	0.5925699	0.988627	1.7751%
64	Thyroid hormone signaling pathway	ko04919	3	183	0.6452008	0.988627	1.6393%
65	Proteasome	ko03050	3	189	0.6662301	0.988627	1.5873%
66	Pentose and glucuronate interconversions	ko00040	3	215	0.7467702	0.988627	1.3953%
67	FoxO signaling pathway	ko04068	3	215	0.7467702	0.988627	1.3953%
68	Cysteine and methionine metabolism	ko00270	3	285	0.8882758	0.988627	1.0526%
69	Oocyte meiosis	ko04114	3	297	0.9037551	0.988627	1.0101%
70	Cell cycle	ko04110	3	305	0.9129701	0.988627	0.9836%
71	PI3K-Akt signaling pathway	ko04151	3	341	0.9452745	0.988627	0.8798%
72	Ubiquitin mediated proteolysis	ko04120	3	393	0.972759	0.988627	0.7634%
73	Glucosinolate biosynthesis	ko00966	2	16	0.0400609	0.3328133	12.5000%
74	Vitamin B6 metabolism	ko00750	2	20	0.057745	0.415764	10.0000%

75	Riboflavin metabolism	ko00740	2	34	0.134221	0.7071156	5.8824%
76	Aldosterone-regulated sodium reabsorption	ko04960	2	48	0.2232614	0.9644894	4.1667%
77	Fc epsilon RI signaling pathway	ko04664	2	73	0.3863796	0.988627	2.7397%
78	Endocrine and other factor-regulated calcium reabsorption	ko04961	2	80	0.4297876	0.988627	2.5000%
79	T cell receptor signaling pathway	ko04660	2	92	0.5001674	0.988627	2.1739%
80	Retinol metabolism	ko00830	2	95	0.516874	0.988627	2.1053%
81	Gap junction	ko04540	2	114	0.6137619	0.988627	1.7544%
82	Homologous recombination	ko03440	2	118	0.6321604	0.988627	1.6949%
83	Tryptophan metabolism	ko00380	2	123	0.6541864	0.988627	1.6260%
84	Photosynthesis	ko00195	2	125	0.6626974	0.988627	1.6000%
85	Focal adhesion	ko04510	2	125	0.6626974	0.988627	1.6000%
86	beta-Alanine metabolism	ko00410	2	127	0.671039	0.988627	1.5748%
87	Protein export	ko03060	2	132	0.6911612	0.988627	1.5152%
88	Flavonoid biosynthesis	ko00941	2	138	0.7139564	0.988627	1.4493%
89	Circadian rhythm - plant	ko04712	2	145	0.7387486	0.988627	1.3793%
90	TGF-beta signaling pathway	ko04350	2	147	0.7454868	0.988627	1.3605%
91	cGMP-PKG signaling pathway	ko04022	2	163	0.7941904	0.988627	1.2270%
92	2-Oxocarboxylic acid metabolism	ko01210	2	180	0.836733	0.988627	1.1111%
93	Fructose and mannose metabolism	ko00051	2	182	0.841178	0.988627	1.0989%
94	Choline metabolism in cancer	ko05231	2	218	0.9044414	0.988627	0.9174%
95	Cell cycle - yeast	ko04111	2	237	0.9274573	0.988627	0.8439%
96	Sphingolipid signaling pathway	ko04071	2	248	0.9382786	0.988627	0.8065%
97	RNA degradation	ko03018	2	341	0.9849613	0.9895425	0.5865%
98	Insulin secretion	ko04911	1	8	0.1491821	0.7672223	12.5000%
99	Biosynthesis of ansamycins	ko01051	1	10	0.1791895	0.8793777	10.0000%
100	Other types of O-glycan biosynthesis	ko00514	1	13	0.2222302	0.9644894	7.6923%

101	Tetracycline biosynthesis	ko00253	1	14	0.2360701	0.988627	7.1429%
102	Butirosin and neomycin biosynthesis	ko00524	1	18	0.2890134	0.988627	5.5556%
103	Naphthalene degradation	ko00626	1	30	0.4268491	0.988627	3.3333%
104	Taurine and hypotaurine metabolism	ko00430	1	30	0.4268491	0.988627	3.3333%
105	RIG-I-like receptor signaling pathway	ko04622	1	30	0.4268491	0.988627	3.3333%
106	Glycosylphosphatidylinositol(GPI)-anchor biosynthesis	ko00563	1	34	0.4665838	0.988627	2.9412%
107	Carbohydrate digestion and absorption	ko04973	1	35	0.4760799	0.988627	2.8571%
108	Streptomycin biosynthesis	ko00521	1	38	0.503567	0.988627	2.6316%
109	Arachidonic acid metabolism	ko00590	1	45	0.5622314	0.988627	2.2222%
110	Degradation of aromatic compounds	ko01220	1	47	0.5776831	0.988627	2.1277%
111	Drug metabolism - other enzymes	ko00983	1	48	0.5852034	0.988627	2.0833%
112	MAPK signaling pathway - fly	ko04013	1	49	0.59259	0.988627	2.0408%
113	Dorso-ventral axis formation	ko04320	1	52	0.6139707	0.988627	1.9231%
114	Serotonergic synapse	ko04726	1	54	0.6275982	0.988627	1.8519%
115	Cholinergic synapse	ko04725	1	56	0.6407451	0.988627	1.7857%
116	Chloroalkane and chloroalkene degradation	ko00625	1	58	0.6534285	0.988627	1.7241%
117	TNF signaling pathway	ko04668	1	59	0.6596014	0.988627	1.6949%
118	Platelet activation	ko04611	1	67	0.7051911	0.988627	1.4925%
119	One carbon pool by folate	ko00670	1	69	0.7156017	0.988627	1.4493%
120	Prolactin signaling pathway	ko04917	1	70	0.7206684	0.988627	1.4286%
121	Pantothenate and CoA biosynthesis	ko00770	1	70	0.7206684	0.988627	1.4286%
122	Carotenoid biosynthesis	ko00906	1	72	0.730533	0.988627	1.3889%
123	Thyroid hormone synthesis	ko04918	1	72	0.730533	0.988627	1.3889%
124	Long-term depression	ko04730	1	75	0.7446814	0.988627	1.3333%
125	Signaling pathways regulating pluripotency of stem cells	ko04550	1	75	0.7446814	0.988627	1.3333%
126	Selenocompound metabolism	ko00450	1	75	0.7446814	0.988627	1.3333%

127	Circadian entrainment	ko04713	1	75	0.7446814	0.988627	1.3333%
128	ErbB signaling pathway	ko04012	1	82	0.7748754	0.988627	1.2195%
129	Natural killer cell mediated cytotoxicity	ko04650	1	86	0.7904994	0.988627	1.1628%
130	Osteoclast differentiation	ko04380	1	87	0.7942331	0.988627	1.1494%
131	Sulfur metabolism	ko00920	1	88	0.7979004	0.988627	1.1364%
132	VEGF signaling pathway	ko04370	1	88	0.7979004	0.988627	1.1364%
133	Photosynthesis - antenna proteins	ko00196	1	89	0.8015024	0.988627	1.1236%
134	Vascular smooth muscle contraction	ko04270	1	90	0.8050403	0.988627	1.1111%
135	Stilbenoid, diarylheptanoid and gingerol biosynthesis	ko00945	1	90	0.8050403	0.988627	1.1111%
136	Chemokine signaling pathway	ko04062	1	91	0.8085152	0.988627	1.0989%
137	Circadian rhythm	ko04710	1	96	0.8249834	0.988627	1.0417%
138	Lysine degradation	ko00310	1	96	0.8249834	0.988627	1.0417%
139	Mismatch repair	ko03430	1	97	0.8281033	0.988627	1.0309%
140	Propanoate metabolism	ko00640	1	99	0.8341773	0.988627	1.0101%
141	Adipocytokine signaling pathway	ko04920	1	99	0.8341773	0.988627	1.0101%
142	NOD-like receptor signaling pathway	ko04621	1	103	0.8456897	0.988627	0.9709%
143	B cell receptor signaling pathway	ko04662	1	104	0.8484408	0.988627	0.9615%
144	Rap1 signaling pathway	ko04015	1	107	0.8564037	0.988627	0.9346%
145	Melanogenesis	ko04916	1	107	0.8564037	0.988627	0.9346%
146	Adherens junction	ko04520	1	109	0.8614788	0.988627	0.9174%
147	Calcium signaling pathway	ko04020	1	115	0.8756541	0.988627	0.8696%
148	Two-component system	ko02020	1	116	0.8778716	0.988627	0.8621%
149	Axon guidance	ko04360	1	117	0.8800496	0.988627	0.8547%
150	Ascorbate and aldarate metabolism	ko00053	1	127	0.8998056	0.988627	0.7874%
151	GnRH signaling pathway	ko04912	1	131	0.9067657	0.988627	0.7634%
152	Tyrosine metabolism	ko00350	1	131	0.9067657	0.988627	0.7634%

153	Long-term potentiation	ko04720	1	134	0.911667	0.988627	0.7463%
154	Progesterone-mediated oocyte maturation	ko04914	1	135	0.9132429	0.988627	0.7407%
155	Alanine, aspartate and glutamate metabolism	ko00250	1	142	0.9235153	0.988627	0.7042%
156	Nucleotide excision repair	ko03420	1	147	0.9301001	0.988627	0.6803%
157	Glutamatergic synapse	ko04724	1	150	0.9337759	0.988627	0.6667%
158	Adrenergic signaling in cardiomyocytes	ko04261	1	153	0.9372587	0.988627	0.6536%
159	Valine, leucine and isoleucine degradation	ko00280	1	157	0.9416195	0.988627	0.6369%
160	Porphyrin and chlorophyll metabolism	ko00860	1	160	0.9446902	0.988627	0.6250%
161	Ras signaling pathway	ko04014	1	167	0.9512424	0.988627	0.5988%
162	Fatty acid biosynthesis	ko00061	1	178	0.9600078	0.988627	0.5618%
163	Wnt signaling pathway	ko04310	1	181	0.9621123	0.988627	0.5525%
164	Fatty acid degradation	ko00071	1	184	0.9641061	0.988627	0.5435%
165	Citrate cycle (TCA cycle)	ko00020	1	197	0.9716035	0.988627	0.5076%
166	Regulation of actin cytoskeleton	ko04810	1	205	0.9754172	0.988627	0.4878%
167	Fc gamma R-mediated phagocytosis	ko04666	1	210	0.9775364	0.988627	0.4762%
168	cAMP signaling pathway	ko04024	1	211	0.9779378	0.988627	0.4739%
169	Glycerolipid metabolism	ko00561	1	215	0.979473	0.988627	0.4651%
170	Phagosome	ko04145	1	257	0.9903783	0.9903783	0.3891%
