# Supplementary material for: Transcriptome Analysis of Genes Associated with the Artemisinin Biosynthesis by Jasmonic Acid Treatment under the Light in Artemisia annua
Source: Front Plant Sci. 2017 Jun 8;8:971. doi: 10.3389/fpls.2017.00971 (PMC5463050; doi:10.3389/fpls.2017.00971)
Supplement: Supplementary file 9 [file Table9.PDF]

Table S9 DEGs between Dark and Dark-MeJA-4h annotated with KEGG metabolic pathways.

NO.	Pathway	Pathway ID	Sample number	Background number	P-Value	Corrected P-Value	Percentage (%)
1	Plant-pathogen interaction	ko04626	36	468	8.31E-06	0.002095	7.6923%
2	Phenylpropanoid biosynthesis	ko00940	32	417	2.69E-05	0.003385	7.6739%
3	Plant hormone signal transduction	ko04075	28	597	0.049127	0.353714	4.6901%
4	Starch and sucrose metabolism	ko00500	24	594	0.194698	0.766623	4.0404%
5	Protein processing in endoplasmic reticulum	ko04141	23	769	0.713958	0.999993	2.9909%
6	Phenylalanine metabolism	ko00360	22	248	6.86E-05	0.005414	8.8710%
7	Spliceosome	ko03040	22	622	0.409953	0.999993	3.5370%
8	Neurotrophin signaling pathway	ko04722	21	368	0.014914	0.190947	5.7065%
9	Estrogen signaling pathway	ko04915	20	313	0.00581	0.121985	6.3898%
10	Carbon metabolism	ko01200	19	893	0.984056	0.999993	2.1277%
11	Endocytosis	ko04144	18	523	0.468872	0.999993	3.4417%
12	Biosynthesis of amino acids	ko01230	18	801	0.966036	0.999993	2.2472%
13	alpha-Linolenic acid metabolism	ko00592	17	165	8.59E-05	0.005414	10.3030%
14	DNA replication	ko03030	16	256	0.015155	0.190947	6.2500%
15	Insulin signaling pathway	ko04910	16	268	0.021745	0.235281	5.9701%
16	Aminoacyl-tRNA biosynthesis	ko00970	16	316	0.070578	0.423468	5.0633%
17	Fatty acid metabolism	ko01212	16	363	0.161825	0.656781	4.4077%
18	Biosynthesis of unsaturated fatty acids	ko01040	15	167	0.000825	0.034661	8.9820%
19	Toll-like receptor signaling pathway	ko04620	15	273	0.046	0.353714	5.4945%
20	MAPK signaling pathway	ko04010	14	275	0.083666	0.468529	5.0909%
21	Antigen processing and presentation	ko04612	14	325	0.202254	0.767255	4.3077%
22	Drug metabolism - cytochrome P450	ko00982	13	137	0.001132	0.039361	9.4891%

23	NF-kappa B signaling pathway	ko04064	13	227	0.046749	0.353714	5.7269%
24	Apoptosis	ko04210	13	230	0.05067	0.354689	5.6522%
25	RNA degradation	ko03018	13	341	0.347837	0.999993	3.8123%
26	Metabolism of xenobiotics by cytochrome P450	ko00980	11	128	0.005279	0.120933	8.5938%
27	Flavonoid biosynthesis	ko00941	11	138	0.00876	0.132526	7.9710%
28	Oxytocin signaling pathway	ko04921	11	192	0.063513	0.390372	5.7292%
29	Oocyte meiosis	ko04114	11	297	0.400231	0.999993	3.7037%
30	Amino sugar and nucleotide sugar metabolism	ko00520	11	315	0.473627	0.999993	3.4921%
31	Pentose phosphate pathway	ko00030	10	165	0.056151	0.372373	6.0606%
32	Linoleic acid metabolism	ko00591	9	73	0.00125	0.039361	12.3288%
33	Retinol metabolism	ko00830	9	95	0.006293	0.121985	9.4737%
34	cGMP-PKG signaling pathway	ko04022	9	163	0.102749	0.517857	5.5215%
35	PPAR signaling pathway	ko03320	9	170	0.122815	0.552666	5.2941%
36	Pyrimidine metabolism	ko00240	9	280	0.581214	0.999993	3.2143%
37	AMPK signaling pathway	ko04152	9	299	0.656462	0.999993	3.0100%
38	Glycolysis / Gluconeogenesis	ko00010	9	442	0.953732	0.999993	2.0362%
39	Calcium signaling pathway	ko04020	8	115	0.044673	0.353714	6.9565%
40	GnRH signaling pathway	ko04912	8	131	0.079209	0.463375	6.1069%
41	Ras signaling pathway	ko04014	8	167	0.200718	0.767255	4.7904%
42	cAMP signaling pathway	ko04024	8	211	0.403881	0.999993	3.7915%
43	Cell cycle	ko04110	8	305	0.787685	0.999993	2.6230%
44	Purine metabolism	ko00230	8	390	0.942252	0.999993	2.0513%
45	Stilbenoid, diarylheptanoid and gingerol biosynthesis	ko00945	7	90	0.036483	0.317022	7.7778%
46	Sesquiterpenoid and triterpenoid biosynthesis	ko00909	7	99	0.054379	0.370364	7.0707%
47	Cyanoamino acid metabolism	ko00460	7	169	0.334926	0.999993	4.1420%
48	Thyroid hormone signaling pathway	ko04919	7	183	0.40807	0.999993	3.8251%

49	Synaptic vesicle cycle	ko04721	7	186	0.423771	0.999993	3.7634%
50	PI3K-Akt signaling pathway	ko04151	7	341	0.931251	0.999993	2.0528%
51	RNA transport	ko03013	7	507	0.997477	0.999993	1.3807%
52	Ribosome	ko03010	7	752	0.999993	0.999993	0.9309%
53	Circadian entrainment	ko04713	6	75	0.045698	0.353714	8.0000%
54	Vascular smooth muscle contraction	ko04270	6	90	0.088241	0.483409	6.6667%
55	Mismatch repair	ko03430	6	97	0.113589	0.540081	6.1856%
56	Rap1 signaling pathway	ko04015	6	107	0.155372	0.641864	5.6075%
57	Melanogenesis	ko04916	6	107	0.155372	0.641864	5.6075%
58	Long-term potentiation	ko04720	6	134	0.293071	0.959141	4.4776%
59	Circadian rhythm - plant	ko04712	6	145	0.355171	0.999993	4.1379%
60	Adrenergic signaling in cardiomyocytes	ko04261	6	153	0.400931	0.999993	3.9216%
61	Phosphatidylinositol signaling system	ko04070	6	155	0.412361	0.999993	3.8710%
62	Terpenoid backbone biosynthesis	ko00900	6	169	0.491253	0.999993	3.5503%
63	Fc gamma R-mediated phagocytosis	ko04666	6	210	0.693762	0.999993	2.8571%
64	FoxO signaling pathway	ko04068	6	215	0.71439	0.999993	2.7907%
65	Choline metabolism in cancer	ko05231	6	218	0.726298	0.999993	2.7523%
66	Glutathione metabolism	ko00480	6	234	0.783878	0.999993	2.5641%
67	Cell cycle - yeast	ko04111	6	237	0.793578	0.999993	2.5316%
68	Methane metabolism	ko00680	6	241	0.805986	0.999993	2.4896%
69	Glycine, serine and threonine metabolism	ko00260	6	263	0.864082	0.999993	2.2814%
70	Carbon fixation in photosynthetic organisms	ko00710	6	268	0.875059	0.999993	2.2388%
71	Cysteine and methionine metabolism	ko00270	6	285	0.906965	0.999993	2.1053%
72	Ubiquitin mediated proteolysis	ko04120	6	393	0.988881	0.999993	1.5267%
73	Oxidative phosphorylation	ko00190	6	427	0.994692	0.999993	1.4052%
74	Degradation of aromatic compounds	ko01220	5	47	0.025157	0.242299	10.6383%

75	Retrograde endocannabinoid signaling	ko04723	5	74	0.109598	0.540081	6.7568%
76	RNA polymerase	ko03020	5	106	0.283791	0.94099	4.7170%
77	Tryptophan metabolism	ko00380	5	123	0.391293	0.999993	4.0650%
78	Ascorbate and aldarate metabolism	ko00053	5	127	0.416689	0.999993	3.9370%
79	Tyrosine metabolism	ko00350	5	131	0.441905	0.999993	3.8168%
80	mTOR signaling pathway	ko04150	5	132	0.44817	0.999993	3.7879%
81	Porphyrin and chlorophyll metabolism	ko00860	5	160	0.612161	0.999993	3.1250%
82	Fructose and mannose metabolism	ko00051	5	182	0.718734	0.999993	2.7473%
83	Fatty acid degradation	ko00071	5	184	0.727298	0.999993	2.7174%
84	Central carbon metabolism in cancer	ko05230	5	194	0.767284	0.999993	2.5773%
85	Lysosome	ko04142	5	203	0.799318	0.999993	2.4631%
86	Pentose and glucuronate interconversions	ko00040	5	215	0.836495	0.999993	2.3256%
87	Galactose metabolism	ko00052	5	218	0.844854	0.999993	2.2936%
88	Sphingolipid signaling pathway	ko04071	5	248	0.910486	0.999993	2.0161%
89	Glycerophospholipid metabolism	ko00564	5	248	0.910486	0.999993	2.0161%
90	Olfactory transduction	ko04740	4	22	0.00894	0.132526	18.1818%
91	Gastric acid secretion	ko04971	4	22	0.00894	0.132526	18.1818%
92	Phototransduction	ko04744	4	26	0.014817	0.190947	15.3846%
93	Salivary secretion	ko04970	4	27	0.016591	0.199088	14.8148%
94	Phototransduction - fly	ko04745	4	28	0.018491	0.211807	14.2857%
95	Naphthalene degradation	ko00626	4	30	0.022683	0.235281	13.3333%
96	Inflammatory mediator regulation of TRP channels	ko04750	4	42	0.059273	0.382993	9.5238%
97	Zeatin biosynthesis	ko00908	4	43	0.063214	0.390372	9.3023%
98	Chloroalkane and chloroalkene degradation	ko00625	4	58	0.137351	0.596768	6.8966%
99	Circadian rhythm	ko04710	4	96	0.39919	0.999993	4.1667%
100	Adipocytokine signaling pathway	ko04920	4	99	0.420876	0.999993	4.0404%

101	Homologous recombination	ko03440	4	118	0.551772	0.999993	3.3898%
102	Bile secretion	ko04976	4	124	0.589783	0.999993	3.2258%
103	Dopaminergic synapse	ko04728	4	135	0.654282	0.999993	2.9630%
104	Phenylalanine, tyrosine and tryptophan biosynthesis	ko00400	4	136	0.659795	0.999993	2.9412%
105	Nucleotide excision repair	ko03420	4	147	0.716457	0.999993	2.7211%
106	TGF-beta signaling pathway	ko04350	4	147	0.716457	0.999993	2.7211%
107	Glutamatergic synapse	ko04724	4	150	0.730642	0.999993	2.6667%
108	Meiosis - yeast	ko04113	4	179	0.841162	0.999993	2.2346%
109	2-Oxocarboxylic acid metabolism	ko01210	4	180	0.844176	0.999993	2.2222%
110	Proteasome	ko03050	4	189	0.869189	0.999993	2.1164%
111	HIF-1 signaling pathway	ko04066	4	200	0.894997	0.999993	2.0000%
112	Regulation of actin cytoskeleton	ko04810	4	205	0.90517	0.999993	1.9512%
113	ABC transporters	ko02010	4	217	0.926102	0.999993	1.8433%
114	mRNA surveillance pathway	ko03015	4	314	0.991904	0.999993	1.2739%
115	Biosynthesis of ansamycins	ko01051	3	10	0.00738	0.132526	30.0000%
116	Carbohydrate digestion and absorption	ko04973	3	35	0.121439	0.552666	8.5714%
117	Diterpenoid biosynthesis	ko00904	3	46	0.207037	0.767255	6.5217%
118	Glycosaminoglycan degradation	ko00531	3	47	0.215412	0.786721	6.3830%
119	Aldosterone-regulated sodium reabsorption	ko04960	3	48	0.223856	0.805881	6.2500%
120	Monoterpenoid biosynthesis	ko00902	3	52	0.258204	0.891333	5.7692%
121	Carotenoid biosynthesis	ko00906	3	72	0.432905	0.999993	4.1667%
122	Thyroid hormone synthesis	ko04918	3	72	0.432905	0.999993	4.1667%
123	Fc epsilon RI signaling pathway	ko04664	3	73	0.441383	0.999993	4.1096%
124	Selenocompound metabolism	ko00450	3	75	0.458189	0.999993	4.0000%
125	GABAergic synapse	ko04727	3	85	0.538575	0.999993	3.5294%
126	Regulation of autophagy	ko04140	3	88	0.561329	0.999993	3.4091%

127	Ether lipid metabolism	ko00565	3	89	0.56876	0.999993	3.3708%
128	T cell receptor signaling pathway	ko04660	3	92	0.590581	0.999993	3.2609%
129	NOD-like receptor signaling pathway	ko04621	3	103	0.664271	0.999993	2.9126%
130	Axon guidance	ko04360	3	117	0.743411	0.999993	2.5641%
131	Fanconi anemia pathway	ko03460	3	122	0.76781	0.999993	2.4590%
132	Focal adhesion	ko04510	3	125	0.781519	0.999993	2.4000%
133	Protein export	ko03060	3	132	0.810916	0.999993	2.2727%
134	Progesterone-mediated oocyte maturation	ko04914	3	135	0.822452	0.999993	2.2222%
135	Alanine, aspartate and glutamate metabolism	ko00250	3	142	0.847051	0.999993	2.1127%
136	Inositol phosphate metabolism	ko00562	3	156	0.887477	0.999993	1.9231%
137	Arginine and proline metabolism	ko00330	3	219	0.974707	0.999993	1.3699%
138	Ribosome biogenesis in eukaryotes	ko03008	3	239	0.984703	0.999993	1.2552%
139	Glucosinolate biosynthesis	ko00966	2	16	0.111982	0.540081	12.5000%
140	Butirosin and neomycin biosynthesis	ko00524	2	18	0.133461	0.590037	11.1111%
141	RIG-I-like receptor signaling pathway	ko04622	2	30	0.273687	0.91959	6.6667%
142	Riboflavin metabolism	ko00740	2	34	0.321419	0.999993	5.8824%
143	Streptomycin biosynthesis	ko00521	2	38	0.368334	0.999993	5.2632%
144	MAPK signaling pathway - fly	ko04013	2	49	0.489428	0.999993	4.0816%
145	Dorso-ventral axis formation	ko04320	2	52	0.519871	0.999993	3.8462%
146	Serotonergic synapse	ko04726	2	54	0.539481	0.999993	3.7037%
147	Cholinergic synapse	ko04725	2	56	0.558528	0.999993	3.5714%
148	TNF signaling pathway	ko04668	2	59	0.58603	0.999993	3.3898%
149	Platelet activation	ko04611	2	67	0.653067	0.999993	2.9851%
150	Prolactin signaling pathway	ko04917	2	70	0.675873	0.999993	2.8571%
151	Steroid biosynthesis	ko00100	2	74	0.704363	0.999993	2.7027%
152	Long-term depression	ko04730	2	75	0.711151	0.999993	2.6667%

153	Signaling pathways regulating pluripotency of stem cells	ko04550	2	75	0.711151	0.999993	2.6667%
154	SNARE interactions in vesicular transport	ko04130	2	78	0.730735	0.999993	2.5641%
155	p53 signaling pathway	ko04115	2	81	0.749177	0.999993	2.4691%
156	ErbB signaling pathway	ko04012	2	82	0.755079	0.999993	2.4390%
157	Natural killer cell mediated cytotoxicity	ko04650	2	86	0.777498	0.999993	2.3256%
158	Base excision repair	ko03410	2	87	0.782815	0.999993	2.2989%
159	Osteoclast differentiation	ko04380	2	87	0.782815	0.999993	2.2989%
160	VEGF signaling pathway	ko04370	2	88	0.788019	0.999993	2.2727%
161	Chemokine signaling pathway	ko04062	2	91	0.802978	0.999993	2.1978%
162	Lysine degradation	ko00310	2	96	0.82583	0.999993	2.0833%
163	Nitrogen metabolism	ko00910	2	101	0.846268	0.999993	1.9802%
164	B cell receptor signaling pathway	ko04662	2	104	0.857459	0.999993	1.9231%
165	Adherens junction	ko04520	2	109	0.874467	0.999993	1.8349%
166	Gap junction	ko04540	2	114	0.889585	0.999993	1.7544%
167	Wnt signaling pathway	ko04310	2	181	0.981935	0.999993	1.1050%
168	Citrate cycle (TCA cycle)	ko00020	2	197	0.988487	0.999993	1.0152%
169	Glycerolipid metabolism	ko00561	2	215	0.993106	0.999993	0.9302%
170	Glyoxylate and dicarboxylate metabolism	ko00630	2	229	0.995391	0.999993	0.8734%
171	Peroxisome	ko04146	2	235	0.996126	0.999993	0.8511%
172	Fluorobenzoate degradation	ko00364	1	7	0.229376	0.814122	14.2857%
173	Insulin secretion	ko04911	1	8	0.254072	0.889254	12.5000%
174	Non-homologous end-joining	ko03450	1	10	0.301118	0.960529	10.0000%
175	Toluene degradation	ko00623	1	12	0.345199	0.999993	8.3333%
176	Ovarian steroidogenesis	ko04913	1	12	0.345199	0.999993	8.3333%
177	Chlorocyclohexane and chlorobenzene degradation	ko00361	1	16	0.425199	0.999993	6.2500%
178	Styrene degradation	ko00643	1	17	0.443623	0.999993	5.8824%

179	Vitamin B6 metabolism	ko00750	1	20	0.495431	0.999993	5.0000%
180	Brassinosteroid biosynthesis	ko00905	1	25	0.571286	0.999993	4.0000%
181	Steroid hormone biosynthesis	ko00140	1	28	0.611212	0.999993	3.5714%
182	Mineral absorption	ko04978	1	43	0.761551	0.999993	2.3256%
183	Lysine biosynthesis	ko00300	1	43	0.761551	0.999993	2.3256%
184	Drug metabolism - other enzymes	ko00983	1	48	0.797415	0.999993	2.0833%
185	Cytosolic DNA-sensing pathway	ko04623	1	50	0.810202	0.999993	2.0000%
186	Notch signaling pathway	ko04330	1	52	0.822183	0.999993	1.9231%
187	Collecting duct acid secretion	ko04966	1	63	0.875777	0.999993	1.5873%
188	Tropane, piperidine and pyridine alkaloid biosynthesis	ko00960	1	64	0.879763	0.999993	1.5625%
189	Vasopressin-regulated water reabsorption	ko04962	1	64	0.879763	0.999993	1.5625%
190	Bacterial secretion system	ko03070	1	68	0.894468	0.999993	1.4706%
191	One carbon pool by folate	ko00670	1	69	0.897855	0.999993	1.4493%
192	Valine, leucine and isoleucine biosynthesis	ko00290	1	76	0.918706	0.999993	1.3158%
193	Butanoate metabolism	ko00650	1	83	0.935303	0.999993	1.2048%
194	Hippo signaling pathway - fly	ko04391	1	88	0.945041	0.999993	1.1364%
195	N-Glycan biosynthesis	ko00510	1	93	0.953315	0.999993	1.0753%
196	Ubiquinone and other terpenoid-quinone biosynthesis	ko00130	1	99	0.961617	0.999993	1.0101%
197	Hippo signaling pathway	ko04390	1	118	0.979356	0.999993	0.8475%
198	Fatty acid biosynthesis	ko00061	1	178	0.997093	0.999993	0.5618%
199	Phagosome	ko04145	1	257	0.999781	0.999993	0.3891%
200	Pyruvate metabolism	ko00620	1	340	0.999986	0.999993	0.2941%
