# Supplementary material for: Transcriptome Analysis of Genes Associated with the Artemisinin Biosynthesis by Jasmonic Acid Treatment under the Light in Artemisia annua
Source: Front Plant Sci. 2017 Jun 8;8:971. doi: 10.3389/fpls.2017.00971 (PMC5463050; doi:10.3389/fpls.2017.00971)
Supplement: Supplementary file 10 [file Table10.PDF]

Table S10 DEGs between Light-MeJA-4h and Dark-MeJA-4h annotated with KEGG metabolic pathways.

NO.	Pathway	Pathway ID	Sample number	Background number	P-Value	Corrected P-Value	Percentage (%)
1	Biosynthesis of amino acids	ko01230	120	801	0.0008768	0.0209758	14.9813%
2	Carbon metabolism	ko01200	116	893	0.0417636	0.360791	12.9899%
3	Ribosome	ko03010	90	752	0.2107075	0.8622372	11.9681%
4	Phenylpropanoid biosynthesis	ko00940	86	417	2.79E-07	2.89E-05	20.6235%
5	Starch and sucrose metabolism	ko00500	86	594	0.0091846	0.1043268	14.4781%
6	Plant hormone signal transduction	ko04075	72	597	0.2210755	0.8910116	12.0603%
7	Cell cycle	ko04110	61	305	3.03E-05	0.0018865	20.0000%
8	Glycolysis / Gluconeogenesis	ko00010	60	442	0.0647806	0.4715327	13.5747%
9	Protein processing in endoplasmic reticulum	ko04141	58	769	0.9982039	1	7.5423%
10	Fatty acid metabolism	ko01212	57	363	0.0078604	0.0977832	15.7025%
11	Plant-pathogen interaction	ko04626	57	468	0.2296326	0.8926966	12.1795%
12	Purine metabolism	ko00230	56	390	0.0339248	0.3403427	14.3590%
13	Phenylalanine metabolism	ko00360	49	248	0.0002206	0.0080023	19.7581%
14	Neurotrophin signaling pathway	ko04722	47	368	0.1694816	0.7866982	12.7717%
15	Carbon fixation in photosynthetic organisms	ko00710	44	268	0.0093928	0.1043268	16.4179%
16	Amino sugar and nucleotide sugar metabolism	ko00520	44	315	0.0741277	0.5239482	13.9683%
17	Cell cycle - yeast	ko04111	43	237	0.0021988	0.0427388	18.1435%
18	DNA replication	ko03030	43	256	0.007277	0.0967441	16.7969%
19	Pyrimidine metabolism	ko00240	42	280	0.0355128	0.3451398	15.0000%
20	Fatty acid biosynthesis	ko00061	38	178	0.0002944	0.0083247	21.3483%
21	Toll-like receptor signaling pathway	ko04620	38	273	0.0942224	0.5932656	13.9194%
22	Glycine, serine and threonine metabolism	ko00260	37	263	0.0881701	0.5712689	14.0684%

23	Pyruvate metabolism	ko00620	37	340	0.5263371	1	10.8824%
24	RNA transport	ko03013	36	507	0.9966461	1	7.1006%
25	Endocytosis	ko04144	36	523	0.9982538	1	6.8834%
26	RNA degradation	ko03018	35	341	0.6556827	1	10.2639%
27	Photosynthesis	ko00195	34	125	1.08E-05	0.0008426	27.2000%
28	Photosynthesis - antenna proteins	ko00196	33	89	5.04E-08	7.84E-06	37.0787%
29	Meiosis - yeast	ko04113	33	179	0.0054099	0.0801176	18.4358%
30	Insulin signaling pathway	ko04910	33	268	0.277532	0.9794959	12.3134%
31	Flavonoid biosynthesis	ko00941	32	138	0.0002481	0.0080023	23.1884%
32	NF-kappa B signaling pathway	ko04064	32	227	0.1043449	0.624063	14.0969%
33	Apoptosis	ko04210	32	230	0.1164706	0.6707841	13.9130%
34	Porphyrin and chlorophyll metabolism	ko00860	31	160	0.0036812	0.0636036	19.3750%
35	Terpenoid backbone biosynthesis	ko00900	31	169	0.0072804	0.0967441	18.3432%
36	Phenylalanine, tyrosine and tryptophan biosynthesis	ko00400	30	136	0.0007679	0.0199014	22.0588%
37	alpha-Linolenic acid metabolism	ko00592	30	165	0.0090715	0.1043268	18.1818%
38	Arginine and proline metabolism	ko00330	30	219	0.1409466	0.7397993	13.6986%
39	Cysteine and methionine metabolism	ko00270	30	285	0.5975826	1	10.5263%
40	Aminoacyl-tRNA biosynthesis	ko00970	30	316	0.7887328	1	9.4937%
41	Ubiquitin mediated proteolysis	ko04120	30	393	0.9796827	1	7.6336%
42	Progesterone-mediated oocyte maturation	ko04914	29	135	0.0013219	0.0274075	21.4815%
43	Peroxisome	ko04146	29	235	0.2894853	0.9794959	12.3404%
44	Antigen processing and presentation	ko04612	29	325	0.8704576	1	8.9231%
45	Glutathione metabolism	ko00480	28	234	0.3468677	1	11.9658%
46	Methane metabolism	ko00680	27	241	0.4735928	1	11.2033%
47	AMPK signaling pathway	ko04152	27	299	0.8485963	1	9.0301%
48	Estrogen signaling pathway	ko04915	27	313	0.8987995	1	8.6262%

49	Pentose phosphate pathway	ko00030	26	165	0.0549768	0.438405	15.7576%
50	2-Oxocarboxylic acid metabolism	ko01210	26	180	0.1103636	0.6476053	14.4444%
51	Glyoxylate and dicarboxylate metabolism	ko00630	26	229	0.4498984	1	11.3537%
52	Glycerophospholipid metabolism	ko00564	26	248	0.6025041	1	10.4839%
53	Oocyte meiosis	ko04114	26	297	0.8801762	1	8.7542%
54	PI3K-Akt signaling pathway	ko04151	26	341	0.972634	1	7.6246%
55	Valine, leucine and isoleucine degradation	ko00280	25	157	0.0539787	0.438405	15.9236%
56	Biotin metabolism	ko00780	24	87	0.0001679	0.007461	27.5862%
57	Stilbenoid, diarylheptanoid and gingerol biosynthesis	ko00945	24	90	0.0002573	0.0080023	26.6667%
58	Biosynthesis of unsaturated fatty acids	ko01040	24	167	0.1253397	0.6898471	14.3713%
59	FoxO signaling pathway	ko04068	24	215	0.4852698	1	11.1628%
60	PPAR signaling pathway	ko03320	23	170	0.1921423	0.8329415	13.5294%
61	Fructose and mannose metabolism	ko00051	23	182	0.2810308	0.9794959	12.6374%
62	Central carbon metabolism in cancer	ko05230	23	194	0.3815545	1	11.8557%
63	Galactose metabolism	ko00052	23	218	0.5887645	1	10.5505%
64	Phagosome	ko04145	23	257	0.8428199	1	8.9494%
65	Spliceosome	ko03040	23	622	1	1	3.6977%
66	Sesquiterpenoid and triterpenoid biosynthesis	ko00909	22	99	0.0033427	0.0611518	22.2222%
67	Circadian rhythm - plant	ko04712	22	145	0.0953803	0.5932656	15.1724%
68	Alanine, aspartate and glutamate metabolism	ko00250	21	142	0.1199842	0.6784561	14.7887%
69	Pentose and glucuronate interconversions	ko00040	21	215	0.71423	1	9.7674%
70	Oxidative phosphorylation	ko00190	21	427	0.9999806	1	4.9180%
71	Nucleotide excision repair	ko03420	20	147	0.2057221	0.8530608	13.6054%
72	Glycerolipid metabolism	ko00561	20	215	0.7804193	1	9.3023%
73	MAPK signaling pathway	ko04010	20	275	0.9734199	1	7.2727%
74	Tyrosine metabolism	ko00350	19	131	0.1499705	0.7397993	14.5038%

75	Drug metabolism - cytochrome P450	ko00982	19	137	0.1925337	0.8329415	13.8686%
76	Cyanoamino acid metabolism	ko00460	19	169	0.4826796	1	11.2426%
77	Fatty acid degradation	ko00071	19	184	0.6215138	1	10.3261%
78	p53 signaling pathway	ko04115	18	81	0.0074658	0.0967441	22.2222%
79	cAMP signaling pathway	ko04024	18	211	0.8682061	1	8.5308%
80	Fatty acid elongation	ko00062	17	87	0.0248136	0.257234	19.5402%
81	Ubiquinone and other terpenoid-quinone biosynthesis	ko00130	17	99	0.0610704	0.4715327	17.1717%
82	Fanconi anemia pathway	ko03460	17	122	0.2037976	0.8530608	13.9344%
83	Citrate cycle (TCA cycle)	ko00020	17	197	0.8513525	1	8.6294%
84	HIF-1 signaling pathway	ko04066	17	200	0.8658192	1	8.5000%
85	Linoleic acid metabolism	ko00591	16	73	0.0124754	0.1337881	21.9178%
86	Base excision repair	ko03410	16	87	0.0435703	0.3662263	18.3908%
87	Mismatch repair	ko03430	16	97	0.0863502	0.5712689	16.4948%
88	Nitrogen metabolism	ko00910	15	101	0.164386	0.7746067	14.8515%
89	Gap junction	ko04540	15	114	0.2838444	0.9794959	13.1579%
90	Metabolism of xenobiotics by cytochrome P450	ko00980	15	128	0.4338188	1	11.7188%
91	Protein export	ko03060	15	132	0.4775967	1	11.3636%
92	cGMP-PKG signaling pathway	ko04022	15	163	0.7691464	1	9.2025%
93	Synaptic vesicle cycle	ko04721	15	186	0.8969687	1	8.0645%
94	Calcium signaling pathway	ko04020	14	115	0.3880259	1	12.1739%
95	Ras signaling pathway	ko04014	14	167	0.8589343	1	8.3832%
96	Oxytocin signaling pathway	ko04921	14	192	0.9489726	1	7.2917%
97	Lysosome	ko04142	14	203	0.9690352	1	6.8966%
98	Ribosome biogenesis in eukaryotes	ko03008	14	239	0.9949939	1	5.8577%
99	Diterpenoid biosynthesis	ko00904	13	46	0.004159	0.0651764	28.2609%
100	Homologous recombination	ko03440	13	118	0.5266112	1	11.0169%

101	Ascorbate and aldarate metabolism	ko00053	13	127	0.6254711	1	10.2362%
102	beta-Alanine metabolism	ko00410	13	127	0.6254711	1	10.2362%
103	Inositol phosphate metabolism	ko00562	13	156	0.8568215	1	8.3333%
104	Wnt signaling pathway	ko04310	13	181	0.9497673	1	7.1823%
105	Fc gamma R-mediated phagocytosis	ko04666	13	210	0.9878555	1	6.1905%
106	Sphingolipid signaling pathway	ko04071	13	248	0.9985492	1	5.2419%
107	mRNA surveillance pathway	ko03015	13	314	0.9999786	1	4.1401%
108	Valine, leucine and isoleucine biosynthesis	ko00290	12	76	0.1522417	0.7397993	15.7895%
109	Rap1 signaling pathway	ko04015	12	107	0.5059102	1	11.2150%
110	Carbon fixation pathways in prokaryotes	ko00720	12	155	0.9036615	1	7.7419%
111	Regulation of actin cytoskeleton	ko04810	12	205	0.991826	1	5.8537%
112	Carbohydrate digestion and absorption	ko04973	11	35	0.0041914	0.0651764	31.4286%
113	Other glycan degradation	ko00511	11	59	0.0787281	0.5347002	18.6441%
114	Thyroid hormone synthesis	ko04918	11	72	0.1888475	0.8329415	15.2778%
115	GABAergic synapse	ko04727	11	85	0.341459	1	12.9412%
116	Lysine degradation	ko00310	11	96	0.4831036	1	11.4583%
117	Adipocytokine signaling pathway	ko04920	11	99	0.521072	1	11.1111%
118	Long-term potentiation	ko04720	11	134	0.8531323	1	8.2090%
119	Thyroid hormone signaling pathway	ko04919	11	183	0.9861938	1	6.0109%
120	Choline metabolism in cancer	ko05231	11	218	0.9982128	1	5.0459%
121	Arachidonic acid metabolism	ko00590	10	45	0.0398411	0.360791	22.2222%
122	Monoterpenoid biosynthesis	ko00902	10	52	0.0790875	0.5347002	19.2308%
123	Steroid biosynthesis	ko00100	10	74	0.309119	1	13.5135%
124	NOD-like receptor signaling pathway	ko04621	10	103	0.6814482	1	9.7087%
125	Tryptophan metabolism	ko00380	10	123	0.8517	1	8.1301%
126	GnRH signaling pathway	ko04912	10	131	0.8954851	1	7.6336%

127	Glutamatergic synapse	ko04724	10	150	0.9582328	1	6.6667%
128	Zeatin biosynthesis	ko00908	9	43	0.0647381	0.4715327	20.9302%
129	Isoquinoline alkaloid biosynthesis	ko00950	9	63	0.2731794	0.9794959	14.2857%
130	Histidine metabolism	ko00340	9	67	0.328618	1	13.4328%
131	One carbon pool by folate	ko00670	9	69	0.3570938	1	13.0435%
132	Carotenoid biosynthesis	ko00906	9	72	0.4002838	1	12.5000%
133	SNARE interactions in vesicular transport	ko04130	9	78	0.4864305	1	11.5385%
134	Ether lipid metabolism	ko00565	9	89	0.6333403	1	10.1124%
135	Propanoate metabolism	ko00640	9	99	0.7443742	1	9.0909%
136	B cell receptor signaling pathway	ko04662	9	104	0.7903134	1	8.6538%
137	Axon guidance	ko04360	9	117	0.8808203	1	7.6923%
138	Focal adhesion	ko04510	9	125	0.9185134	1	7.2000%
139	ABC transporters	ko02010	9	217	0.9996971	1	4.1475%
140	Retrograde endocannabinoid signaling	ko04723	8	74	0.56174	1	10.8108%
141	Circadian entrainment	ko04713	8	75	0.5758818	1	10.6667%
142	Selenocompound metabolism	ko00450	8	75	0.5758818	1	10.6667%
143	Natural killer cell mediated cytotoxicity	ko04650	8	86	0.7152357	1	9.3023%
144	Osteoclast differentiation	ko04380	8	87	0.726252	1	9.1954%
145	Regulation of autophagy	ko04140	8	88	0.7369767	1	9.0909%
146	Sulfur metabolism	ko00920	8	88	0.7369767	1	9.0909%
147	VEGF signaling pathway	ko04370	8	88	0.7369767	1	9.0909%
148	Vascular smooth muscle contraction	ko04270	8	90	0.7575483	1	8.8889%
149	Chemokine signaling pathway	ko04062	8	91	0.7673954	1	8.7912%
150	Circadian rhythm	ko04710	8	96	0.812298	1	8.3333%
151	Melanogenesis	ko04916	8	107	0.8874035	1	7.4766%
152	Phosphatidylinositol signaling system	ko04070	8	155	0.9925761	1	5.1613%

153	Tropane, piperidine and pyridine alkaloid biosynthesis	ko00960	7	64	0.5535193	1	10.9375%
154	Vasopressin-regulated water reabsorption	ko04962	7	64	0.5535193	1	10.9375%
155	Fc epsilon RI signaling pathway	ko04664	7	73	0.6808401	1	9.5890%
156	Retinol metabolism	ko00830	7	95	0.8836849	1	7.3684%
157	Adherens junction	ko04520	7	109	0.9453072	1	6.4220%
158	Bile secretion	ko04976	7	124	0.9774167	1	5.6452%
159	mTOR signaling pathway	ko04150	7	132	0.9862893	1	5.3030%
160	Adrenergic signaling in cardiomyocytes	ko04261	7	153	0.9965692	1	4.5752%
161	Proteasome	ko03050	7	189	0.9997388	1	3.7037%
162	Streptomycin biosynthesis	ko00521	6	38	0.2603821	0.9526922	15.7895%
163	Degradation of aromatic compounds	ko01220	6	47	0.421405	1	12.7660%
164	Chloroalkane and chloroalkene degradation	ko00625	6	58	0.6089652	1	10.3448%
165	Pancreatic secretion	ko04972	6	62	0.668283	1	9.6774%
166	Bacterial secretion system	ko03070	6	68	0.7458347	1	8.8235%
167	Signaling pathways regulating pluripotency of stem cells	ko04550	6	75	0.8186411	1	8.0000%
168	Butanoate metabolism	ko00650	6	83	0.8804875	1	7.2289%
169	T cell receptor signaling pathway	ko04660	6	92	0.9278078	1	6.5217%
170	Hippo signaling pathway	ko04390	6	118	0.9857454	1	5.0847%
171	Dopaminergic synapse	ko04728	6	135	0.9955507	1	4.4444%
172	Naphthalene degradation	ko00626	5	30	0.2567302	0.9508998	16.6667%
173	Riboflavin metabolism	ko00740	5	34	0.3357952	1	14.7059%
174	Thiamine metabolism	ko00730	5	45	0.5518532	1	11.1111%
175	Glycosaminoglycan degradation	ko00531	5	47	0.5877034	1	10.6383%
176	Drug metabolism - other enzymes	ko00983	5	48	0.6050262	1	10.4167%
177	Cell cycle - Caulobacter	ko04112	5	58	0.7532579	1	8.6207%
178	TNF signaling pathway	ko04668	5	59	0.7654861	1	8.4746%

179	Prolactin signaling pathway	ko04917	5	70	0.8710311	1	7.1429%
180	ErbB signaling pathway	ko04012	5	82	0.9373188	1	6.0976%
181	Hippo signaling pathway - fly	ko04391	5	88	0.957271	1	5.6818%
182	RNA polymerase	ko03020	5	106	0.987412	1	4.7170%
183	Two-component system	ko02020	5	116	0.993861	1	4.3103%
184	Biosynthesis of ansamycins	ko01051	4	10	0.0416065	0.360791	40.0000%
185	Glucosinolate biosynthesis	ko00966	4	16	0.126435	0.6898471	25.0000%
186	Butirosin and neomycin biosynthesis	ko00524	4	18	0.1639484	0.7746067	22.2222%
187	Aminobenzoate degradation	ko00627	4	21	0.2263341	0.8910116	19.0476%
188	C5-Branched dibasic acid metabolism	ko00660	4	33	0.4986109	1	12.1212%
189	Limonene and pinene degradation	ko00903	4	38	0.6013646	1	10.5263%
190	Inflammatory mediator regulation of TRP channels	ko04750	4	42	0.6736909	1	9.5238%
191	Lysine biosynthesis	ko00300	4	43	0.6902749	1	9.3023%
192	MAPK signaling pathway - fly	ko04013	4	49	0.7770985	1	8.1633%
193	Dorso-ventral axis formation	ko04320	4	52	0.812672	1	7.6923%
194	Cutin, suberine and wax biosynthesis	ko00073	4	54	0.8336996	1	7.4074%
195	Serotonergic synapse	ko04726	4	54	0.8336996	1	7.4074%
196	Cholinergic synapse	ko04725	4	56	0.852717	1	7.1429%
197	Long-term depression	ko04730	4	75	0.9579112	1	5.3333%
198	Endocrine and other factor-regulated calcium reabsorption	ko04961	4	80	0.9704612	1	5.0000%
199	Sphingolipid metabolism	ko00600	4	93	0.9886602	1	4.3011%
200	TGF-beta signaling pathway	ko04350	4	147	0.9998555	1	2.7211%
201	Bisphenol degradation	ko00363	3	11	0.1521278	0.7397993	27.2727%
202	Polycyclic aromatic hydrocarbon degradation	ko00624	3	16	0.2847345	0.9794959	18.7500%
203	Olfactory transduction	ko04740	3	22	0.4504817	1	13.6364%
204	Gastric acid secretion	ko04971	3	22	0.4504817	1	13.6364%

205	MAPK signaling pathway - yeast	ko04011	3	22	0.4504817	1	13.6364%
206	Leukocyte transendothelial migration	ko04670	3	25	0.5276976	1	12.0000%
207	Phototransduction	ko04744	3	26	0.5520646	1	11.5385%
208	Salivary secretion	ko04970	3	27	0.5756672	1	11.1111%
209	Phototransduction - fly	ko04745	3	28	0.598473	1	10.7143%
210	Steroid hormone biosynthesis	ko00140	3	28	0.598473	1	10.7143%
211	Mineral absorption	ko04978	3	43	0.8425627	1	6.9767%
212	Platelet activation	ko04611	3	67	0.9731838	1	4.4776%
213	Pantothenate and CoA biosynthesis	ko00770	3	70	0.9788331	1	4.2857%
214	Fat digestion and absorption	ko04975	2	9	0.2948663	0.9794959	22.2222%
215	Anthocyanin biosynthesis	ko00942	2	9	0.2948663	0.9794959	22.2222%
216	D-Glutamine and D-glutamate metabolism	ko00471	2	11	0.3697128	1	18.1818%
217	Lipopolysaccharide biosynthesis	ko00540	2	12	0.405977	1	16.6667%
218	Glycosphingolipid biosynthesis - ganglio series	ko00604	2	13	0.4411971	1	15.3846%
219	Proximal tubule bicarbonate reclamation	ko04964	2	17	0.5692864	1	11.7647%
220	Neuroactive ligand-receptor interaction	ko04080	2	17	0.5692864	1	11.7647%
221	Circadian rhythm - fly	ko04711	2	19	0.6248665	1	10.5263%
222	Vitamin B6 metabolism	ko00750	2	20	0.6504967	1	10.0000%
223	Synthesis and degradation of ketone bodies	ko00072	2	23	0.7190245	1	8.6957%
224	Glycosphingolipid biosynthesis - globo series	ko00603	2	24	0.7392058	1	8.3333%
225	Novobiocin biosynthesis	ko00401	2	27	0.7924369	1	7.4074%
226	Taurine and hypotaurine metabolism	ko00430	2	30	0.8358255	1	6.6667%
227	Protein digestion and absorption	ko04974	2	30	0.8358255	1	6.6667%
228	Benzoate degradation	ko00362	2	33	0.8708357	1	6.0606%
229	Aldosterone-regulated sodium reabsorption	ko04960	2	48	0.9633171	1	4.1667%
230	Cytosolic DNA-sensing pathway	ko04623	2	50	0.9691634	1	4.0000%

231	Cardiac muscle contraction	ko04260	2	52	0.9741059	1	3.8462%
232	Notch signaling pathway	ko04330	2	52	0.9741059	1	3.8462%
233	Basal transcription factors	ko03022	2	81	0.9981197	1	2.4691%
234	N-Glycan biosynthesis	ko00510	2	93	0.9993863	1	2.1505%
235	Aflatoxin biosynthesis	ko00254	1	6	0.514895	1	16.6667%
236	Vitamin digestion and absorption	ko04977	1	7	0.5625268	1	14.2857%
237	Fluorobenzoate degradation	ko00364	1	7	0.5625268	1	14.2857%
238	Insulin secretion	ko04911	1	8	0.6054825	1	12.5000%
239	Flavone and flavonol biosynthesis	ko00944	1	8	0.6054825	1	12.5000%
240	Glycosaminoglycan biosynthesis - chondroitin sulfate / dermatan sulfate	ko00532	1	9	0.6442212	1	11.1111%
241	Non-homologous end-joining	ko03450	1	10	0.6791566	1	10.0000%
242	Polyketide sugar unit biosynthesis	ko00523	1	10	0.6791566	1	10.0000%
243	Glycosaminoglycan biosynthesis - heparan sulfate / heparin	ko00534	1	10	0.6791566	1	10.0000%
244	Ovarian steroidogenesis	ko04913	1	12	0.7390747	1	8.3333%
245	Toluene degradation	ko00623	1	12	0.7390747	1	8.3333%
246	Other types of O-glycan biosynthesis	ko00514	1	13	0.7646976	1	7.6923%
247	Chlorocyclohexane and chlorobenzene degradation	ko00361	1	16	0.8274356	1	6.2500%
248	Styrene degradation	ko00643	1	17	0.8443828	1	5.8824%
249	Hedgehog signaling pathway	ko04340	1	35	0.9758	1	2.8571%
250	Folate biosynthesis	ko00790	1	41	0.9869883	1	2.4390%
251	Nicotinate and nicotinamide metabolism	ko00760	1	52	0.9958294	1	1.9231%
252	Various types of N-glycan biosynthesis	ko00513	1	57	0.9975137	1	1.7544%
253	Collecting duct acid secretion	ko04966	1	63	0.9986635	1	1.5873%
254	Tight junction	ko04530	1	74	0.9995718	1	1.3514%
