# Supplementary material for: Transcriptome Analysis of Genes Associated with the Artemisinin Biosynthesis by Jasmonic Acid Treatment under the Light in Artemisia annua
Source: Front Plant Sci. 2017 Jun 8;8:971. doi: 10.3389/fpls.2017.00971 (PMC5463050; doi:10.3389/fpls.2017.00971)

Figure S1 The summary of NR database annotation. (A) Species distribution of the closest matches for each sequence. (B) E-value distribution of matches for sequence. (C) Similar distribution of matches for sequence.

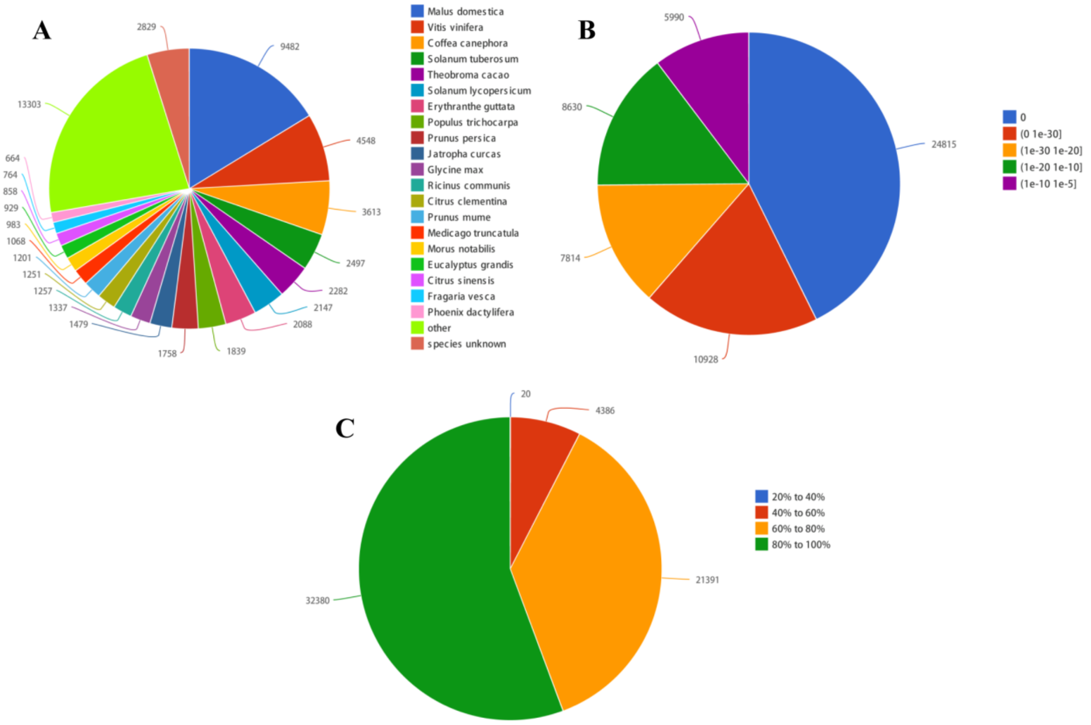

Supplement: Supplementary file 11 [file Image1.PDF]
