# Supplementary material for: Transcriptome Analysis of Genes Associated with the Artemisinin Biosynthesis by Jasmonic Acid Treatment under the Light in Artemisia annua
Source: Front Plant Sci. 2017 Jun 8;8:971. doi: 10.3389/fpls.2017.00971 (PMC5463050; doi:10.3389/fpls.2017.00971)

Figure S2 The volcano plot of the differentially expressed genes (DEGs) between Light and Dark (A), Light and Light-MeJA-4h (B), Dark and Dark-MeJA-4h (C), Light-MeJA-4h and Dark-MeJA-4h (D).

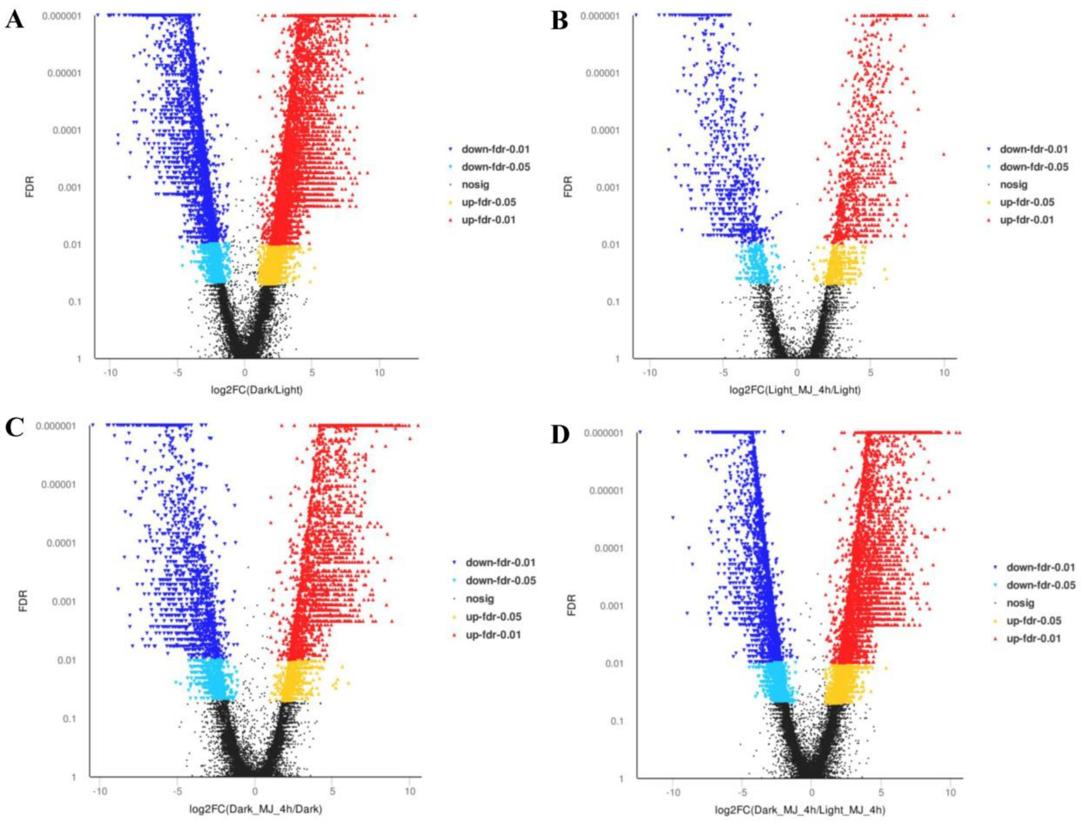

Supplement: Supplementary file 12 [file Image2.PDF]
