# Supplementary figures and images for: Transcriptome Analysis of Genes Associated with the Artemisinin Biosynthesis by Jasmonic Acid Treatment under the Light in Artemisia annua
Source: Front Plant Sci. 2017 Jun 8;8:971. doi: 10.3389/fpls.2017.00971 (PMC5463050; doi:10.3389/fpls.2017.00971)

**Figure S3** DEGs between the Dark and Light annotated with GO classification.

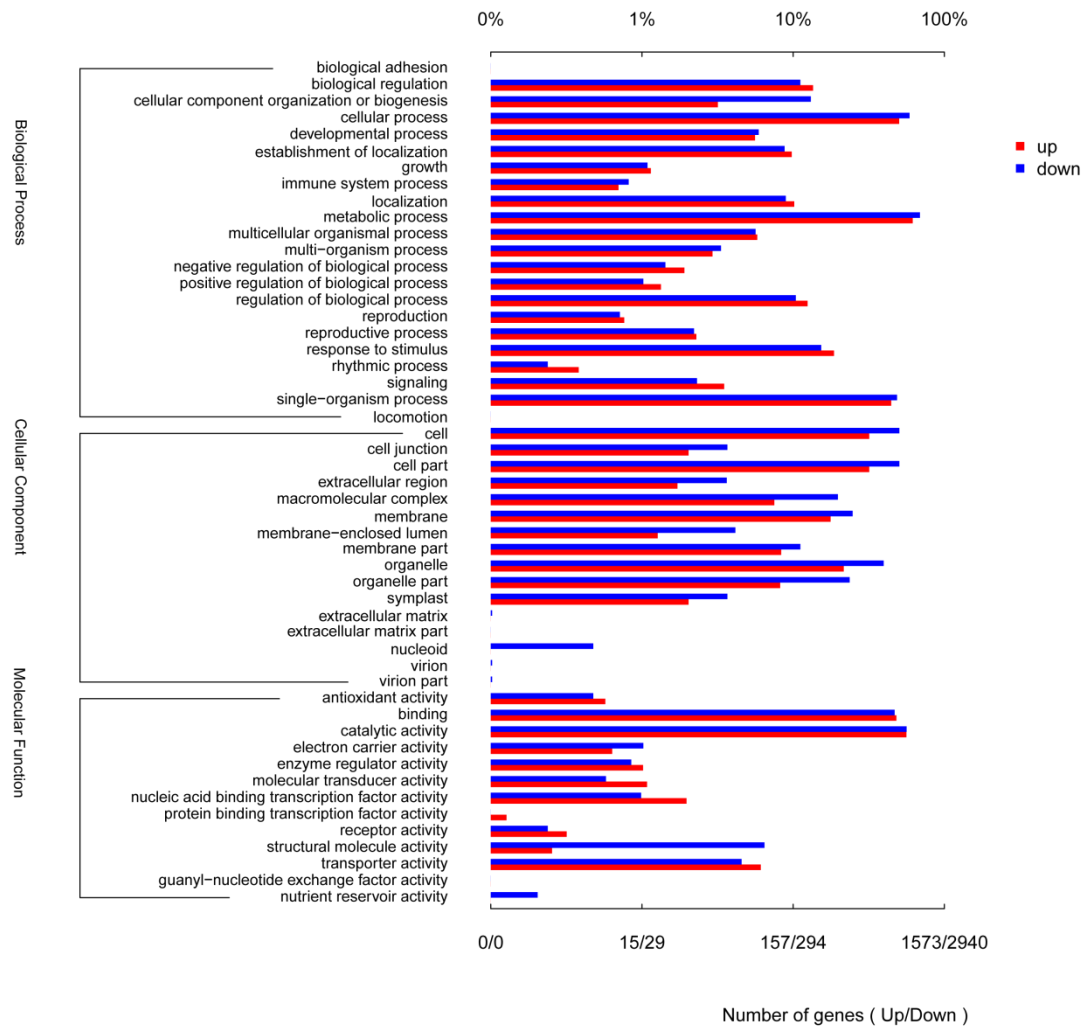

Supplement: Supplementary file 13 [file Image3.PDF]

**Figure S4** DEGs between the Light-MeJA-4h and Light annotated with GO classification.

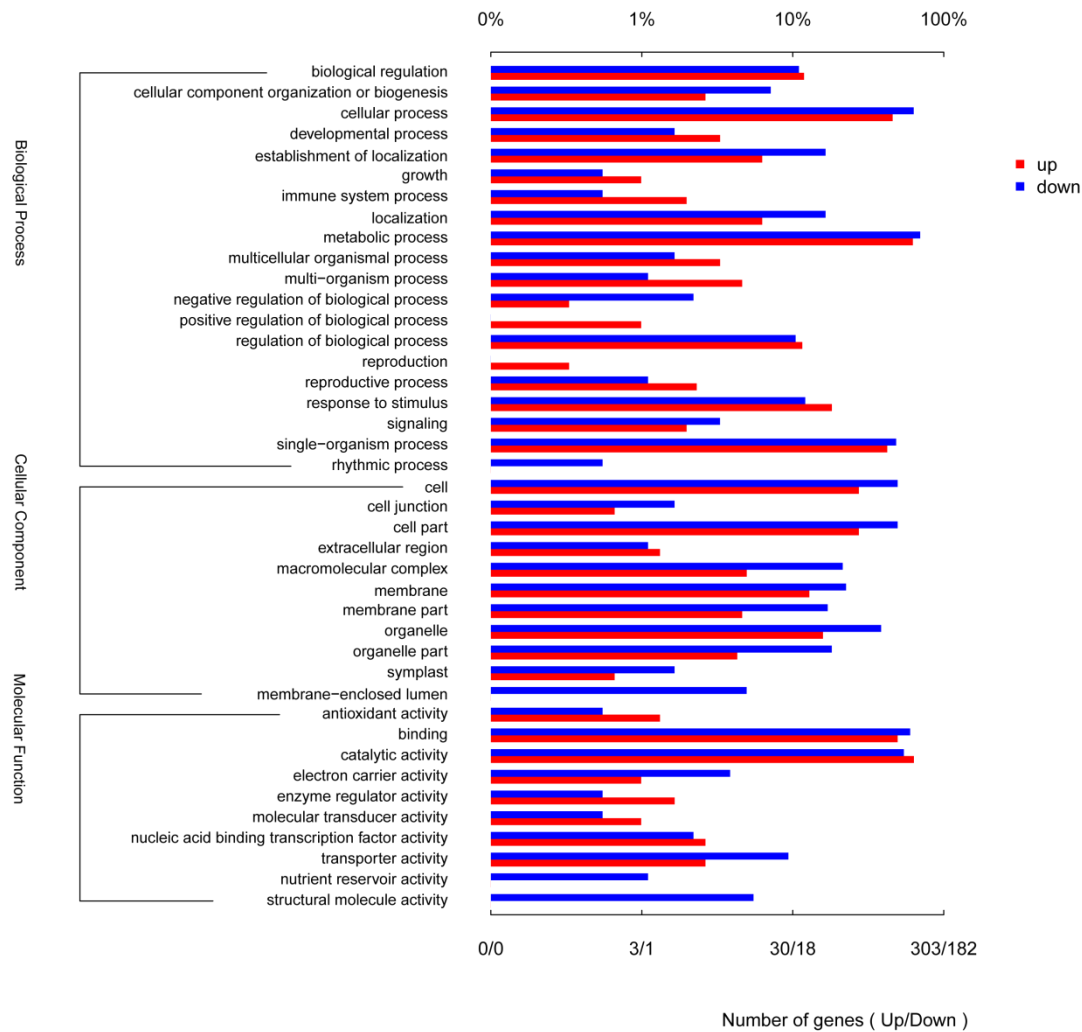

Supplement: Supplementary file 14 [file Image4.PDF]

**Figure S5** DEGs between the Dark and Dark-MeJA-4h annotated with GO classification.

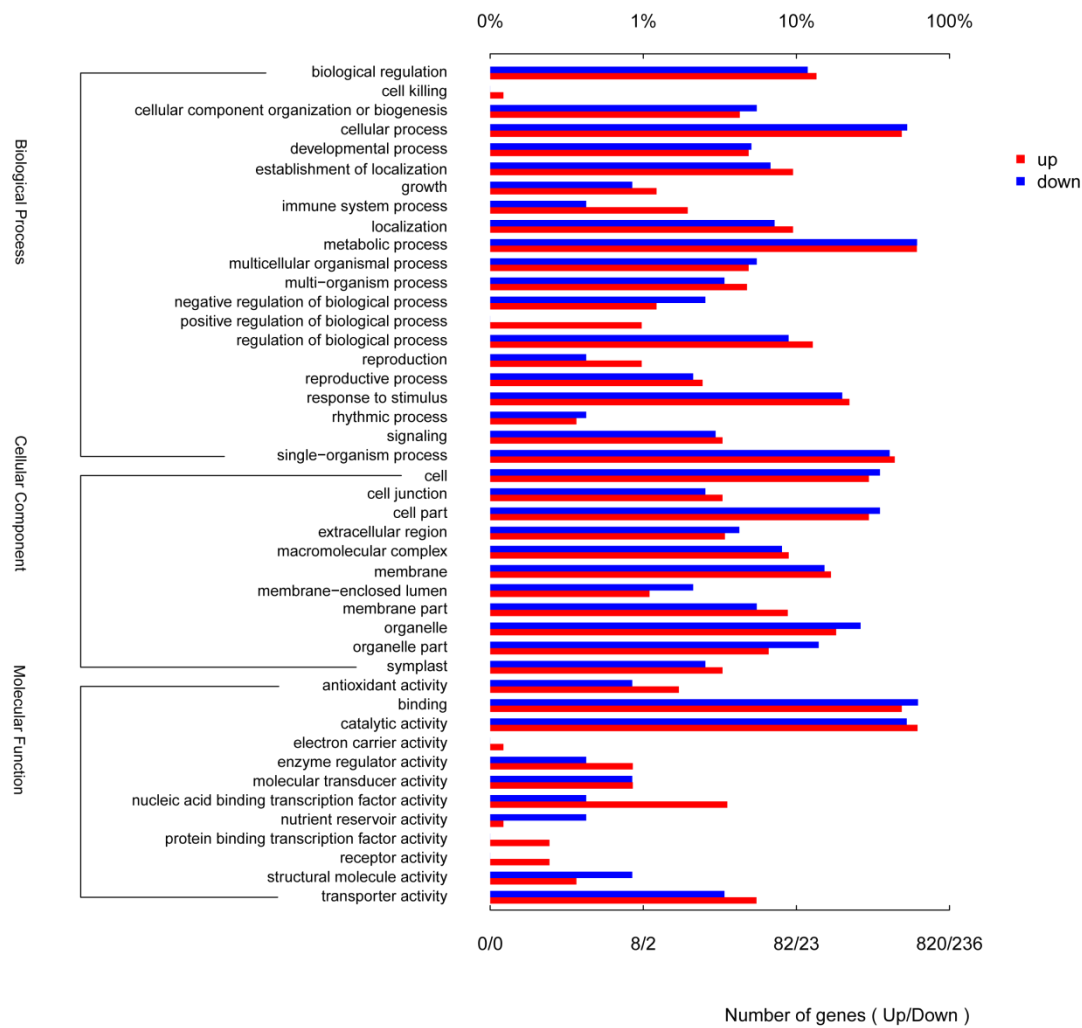

Supplement: Supplementary file 15 [file Image5.PDF]

**Figure S6** DEGs between the Light-MeJA-4h and Dark-MeJA-4h annotated with GO classification.

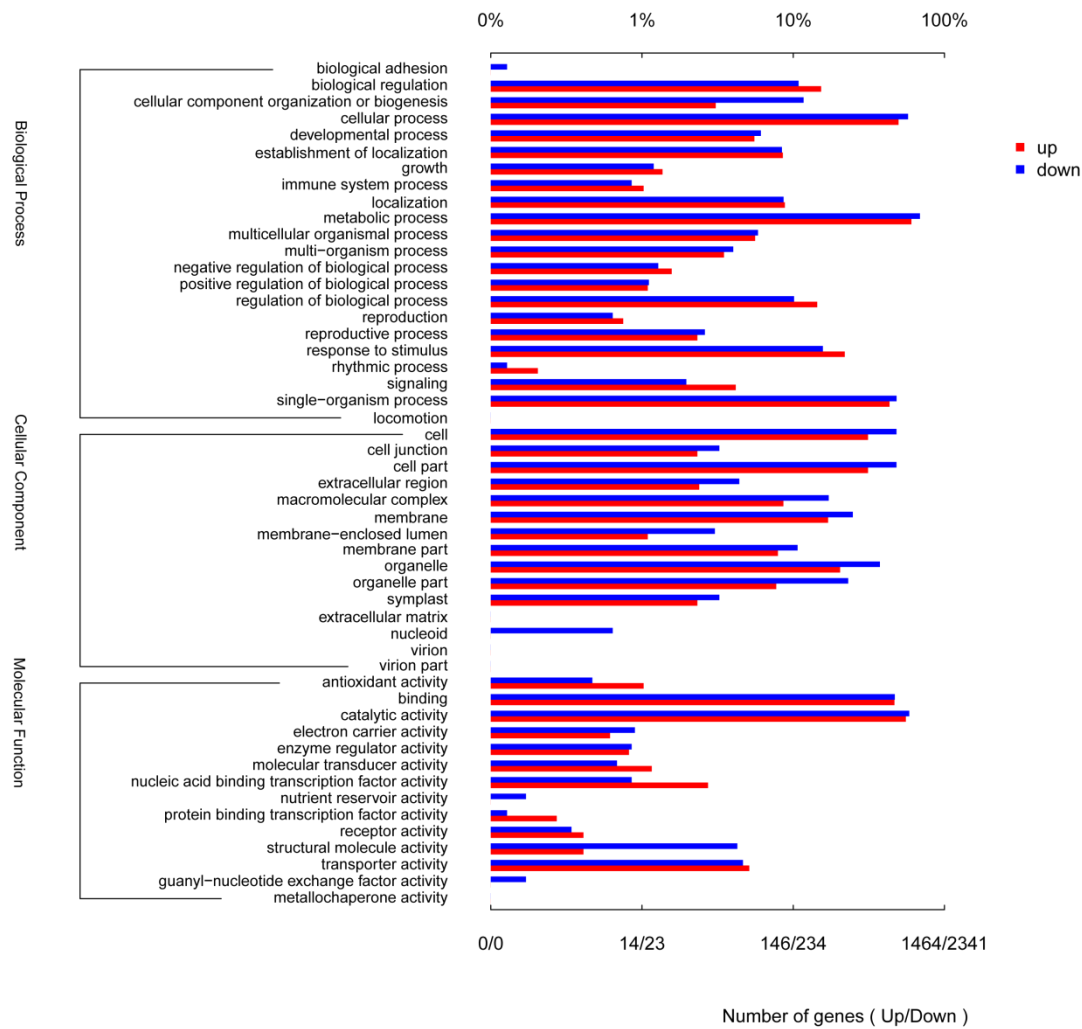

Supplement: Supplementary file 16 [file Image6.pdf]

**Figure S7** The enrichment analyses based on KEGG pathways of DEGs between the Dark and Light.

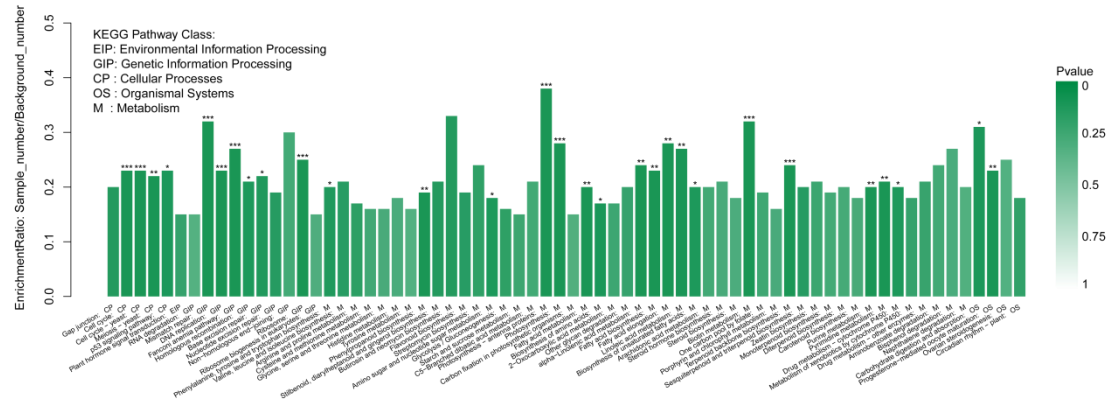

Supplement: Supplementary file 17 [file Image7.PDF]

**Figure S9** The enrichment analyses based on KEGG pathways of DEGs between the Dark and Dark-MeJA-4h.

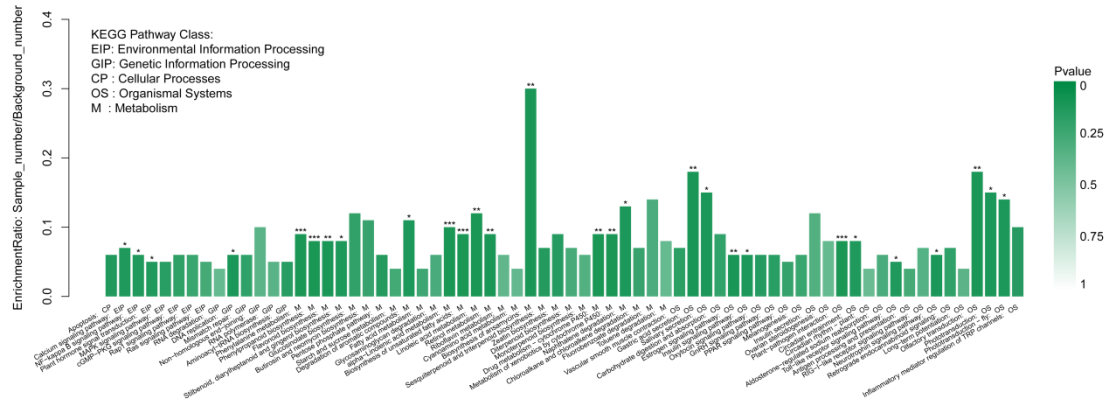

Supplement: Supplementary file 19 [file Image9.PDF]

**Figure S11** Up-regulated and down-regulated TFs between the Dark relative to Dark-MeJA-4h.

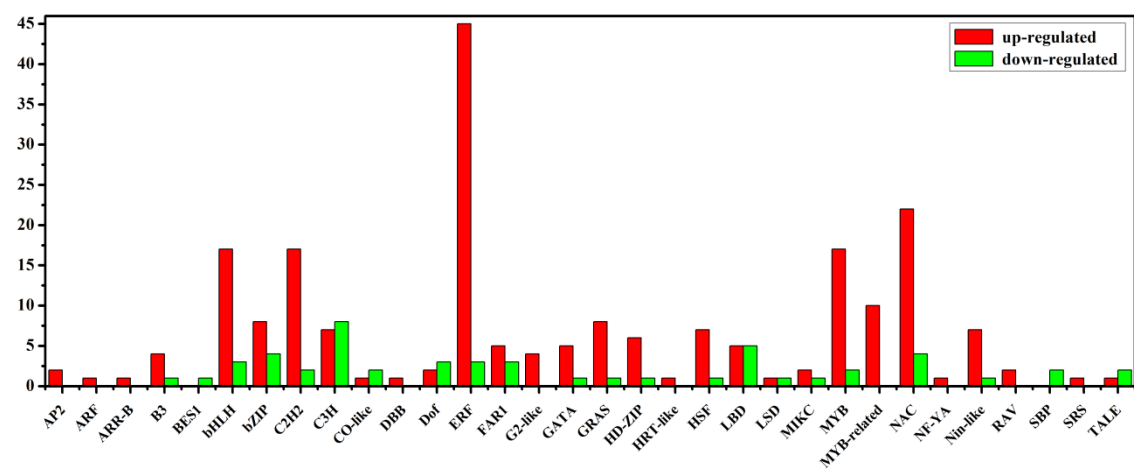

Supplement: Supplementary file 21 [file Image11.PDF]

**Figure S12** Up-regulated and down-regulated TFs between the Light-MeJA-4h relative to Dark-MeJA-4h.

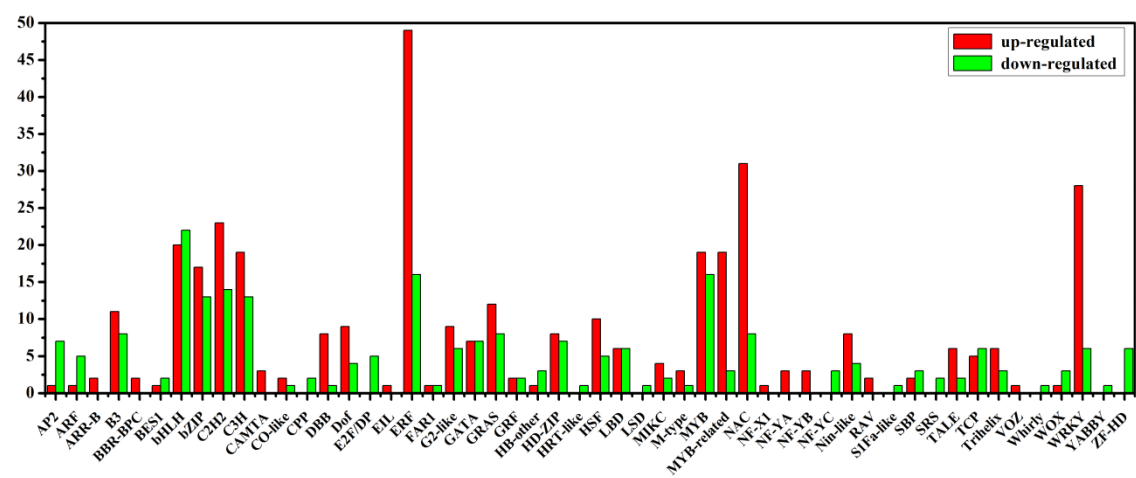

Supplement: Supplementary file 22 [file Image12.PDF]
