# Supplementary material for: Transcriptome Analysis of Genes Associated with the Artemisinin Biosynthesis by Jasmonic Acid Treatment under the Light in Artemisia annua
Source: Front Plant Sci. 2017 Jun 8;8:971. doi: 10.3389/fpls.2017.00971 (PMC5463050; doi:10.3389/fpls.2017.00971)

Figure S10 The enrichment analyses based on KEGG pathways of DEGs between the Light-MeJA-4h and Dark-MeJA-4h.

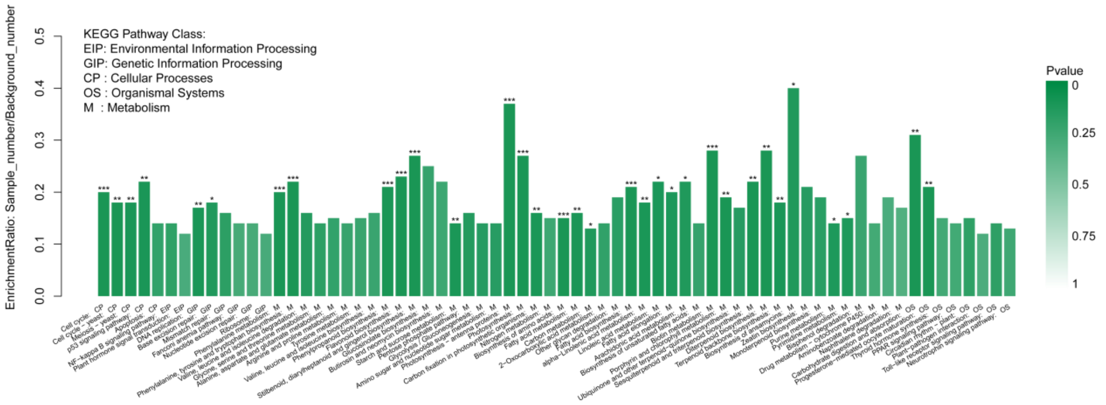

Supplement: Supplementary file 20 [file Image10.PDF]
